# The epidemiology of drug-related hospital admissions in paediatrics – a systematic review

**DOI:** 10.1186/s13690-024-01295-4

**Published:** 2024-06-04

**Authors:** Sonja Eberl, Pauline Heus, Irmgard Toni, Igor Bachmat, Antje Neubert

**Affiliations:** 1 Department of Pediatrics and Adolescent Medicine, Faculty of Medicine, Universitätsklinikum Erlangen, Friedrich-Alexander-Universität Erlangen-Nürnberg, Erlangen, Germany; 2 Cochrane Netherlands, University Medical Center Utrecht, Utrecht University, Utrecht, The Netherlands; 3 Julius Center for Health Sciences and Primary Care, University Medical Center Utrecht, Utrecht University, Utrecht, The Netherlands

**Keywords:** Drug-related hospitalisations, Drug-related problems, Adverse drug events, Adverse drug reactions, Hospital admissions, Hospitalisation, Paediatric, Children, Medication safety

## Abstract

**Background:**

Despite previous efforts, medication safety in paediatrics remains a major concern. To inform improvement strategies and further research especially in outpatient care, we systematically reviewed the literature on the frequency and nature of drug-related hospital admissions in children.

**Methods:**

Searches covered Embase, Medline, Web of Science, grey literature sources and relevant article citations. Studies reporting epidemiological data on paediatric drug-related hospital admissions published between 01/2000 and 01/2024 were eligible. Study identification, data extraction, and critical appraisal were conducted independently in duplicate using templates based on the ’Joanna Briggs Institute’ recommendations.

**Results:**

The review included data from 45 studies reporting > 24,000 hospitalisations for adverse drug events (ADEs) or adverse drug reactions (ADRs). Due to different reference groups, a total of 52 relative frequency values were provided. We stratified these results by study characteristics. As a percentage of inpatients, the highest frequency of drug-related hospitalisation was found with ‘intensive ADE monitoring’, ranging from 3.1% to 5.8% (5 values), whereas with ‘routine ADE monitoring’, it ranged from 0.2% to 1.0% (3 values). The relative frequencies of ‘ADR-related hospitalisations’ ranged from 0.2% to 6.9% for ‘intensive monitoring’ (23 values) and from 0.04% to 3.8% for ‘routine monitoring’ (8 values). Per emergency department visits, five relative frequency values ranged from 0.1% to 3.8% in studies with ‘intensive ADE monitoring’, while all other eight values were ≤ 0.1%. Heterogeneity prevented pooled estimates. Studies rarely reported on the nature of the problems, or studies with broader objectives lacked disaggregated data. Limited data indicated that one in three (median) drug-related admissions could have been prevented, especially by more attentive prescribing. Besides polypharmacy and oncological therapy, no other risk factors could be clearly identified. Insufficient information and a high risk of bias, especially in retrospective and routine observational studies, hampered the assessment.

**Conclusion:**

Given the high frequency of drug-related hospitalisations, medication safety in paediatrics needs to be further improved. As routine identification appears unreliable, clinical awareness needs to be raised. To gain more profound insights especially for generating improvement strategies, we have to address under-reporting and methodological issues in future research.

**Trial registration:**

PROSPERO (CRD42021296986)

**Supplementary Information:**

The online version contains supplementary material available at 10.1186/s13690-024-01295-4.

**Table:** 

Text box 1. Contributions to the literature
• Investigating drug-related hospitalisations offers a basis for improving medication safety. This systematic review provides an up-to-date evaluation of the available evidence in paediatrics
• High frequencies of paediatric patients with drug-related hospitalisations have been reported. It was not possible to pool frequencies from individual studies because of large variations in study design
• Few data have been published on the nature of the underlying problems and preventability could only be cautiously estimated
• Primary studies should be standardised and report more detailed information. Studies using routine data indicate that routine monitoring needs to be improved

## Background

Patient safety has been recognised as a global health priority for 20 years [1, 2]. However, the WHO still ranks adverse events resulting from unsafe care among the top ten causes of death and disability in the world. It therefore calls for continued commitment to optimise patient safety [3, 4]. A major focus is the medication process with its avoidable and unavoidable risks in prescribing and using medication. In this context, children constitute a particularly vulnerable high-risk group [5]. Compared to the medication process in adults, there are some differences associated with higher risks, such as a high rate of off-label medication use, a lack of appropriate dosage forms, or complex dose calculations, and there may be an altered response to drug therapies due to individual stages of the patient’s ontogeny [6–11]. A prerequisite for improving patient safety is to understand the current data on the burden of medication-related problems in children. This concerns data from the inpatient sector, the outpatient sector as well as the transition between the two sectors.

For the group of paediatric inpatients, systematic reviews have already provided valuable insights into the current evidence [6, 12–14] showing that drug-related problems are common during hospitalisation, are often preventable, and tend to be minor or moderate. Polypharmacy has been identified as a risk factor [6, 12, 14]. Important types of problems during hospitalisation include prescribing errors [13, 14], e.g. in drug selection and dosage, and problems with administration. The reviews emphasised the role of adverse drug reactions [13, 14] or were limited to examining this type of drug-related problem [6, 12]. Analyses of the incidence of adverse drug reactions suggest that they may have affected one in six hospitalised children [6, 12, 13].

Beyond this, information from studies on drug-related problems causing hospital admissions is of particular interest in order to gain insights into serious problems occurring in the outpatient setting or among patients discharged from hospital. Their clear and informative endpoint ‘hospitalisation’ is relevant from both the patient’s and society’s perspective. Hospitalisation is a particular distress for children, and the primary problems are usually of considerable concern. For the society, hospital admissions imply high costs, as has already been shown for adults [15–17].

So far, the topic ‘drug-related hospitalisation in paediatrics’ has only partially been addressed in systematic reviews, e.g. because it was predominantly limited to the problem of ‘adverse drug reactions’ [6, 18]. Nevertheless, with pooled estimates of 2.1% [18] and 2.9% [6] of hospital admissions attributed to adverse drug reactions, these reviews showed that there are considerable safety issues within outpatient paediatric drug therapy. The authors therefore recommended further research. Additionally, a comprehensive update is needed as these older reviews [6, 18, 19] may have lost their applicability e.g. due to changes in the drugs most commonly used or in the healthcare system. Some improvements in the medication process may have already been introduced, e.g. personnel support and IT infrastructure [11, 20], and their impact could have become apparent. Although we were aware of a number of recent studies, these have not yet been systematically evaluated. To our knowledge, there has currently been no systematic, up-to-date review on drug-related hospital admissions for paediatric patients.

Consequently, our aim was to systematically review the data regarding the frequency and nature of drug-related hospital admissions in paediatrics. We investigated the nature of drug-related hospitalisations by exploring the drugs involved, the different types of problems and their preventability. In addition, we sought to collate information on important influencing factors that lead to drug-related hospital admissions in paediatrics in order to inform future prevention strategies.

## Methods

For this review, we considered the recommendations for “Systematic reviews of prevalence and incidence” [21] and the “Preferred Reporting Items for Systematic Reviews and Meta-Analysis” (PRISMA) [22] reporting guideline. The protocol is available in PROSPERO (CRD42021296986) [23].

### Identification of relevant literature

#### Definitions

The classification of the Pharmaceutical Care Network Europe (PCNE) elaborates on what constitutes drug-related problems. A drug-related problem is defined as “an event or circumstance involving drug therapy that actually or potentially interferes with desired health outcomes” [24]. Problems may regard treatment effectiveness (no or not optimal effect, untreated symptoms or indication), treatment safety (adverse drug events) or other aspects (e.g. unnecessary drug-treatment). Their causes include an inappropriate selection of the drug, drug form, dose or duration of treatment. In addition, problems can also occur in dispensing and use of medicines or may be patient-related or health system-related [24]. However, previously, various definitions have been used in the literature. There has been no consensus on preference or structure of classification systems of drug-related problems and terms have occasionally been used interchangeably in published studies [13, 19, 25]. Commonly, studies on drug-related hospital admissions address the aspect of treatment safety, usually distinguishing whether they focus on adverse drug reactions (ADRs) or use the broader concept of adverse drug events (ADEs). The former is defined as a harmful and unintended response to a drug, the latter additionally comprises events related to drug use, e.g. due to medication errors [13, 26–28]. This review provides a comprehensive evaluation and searched for studies using any of these different definitions.

#### Information sources

We conducted a comprehensive literature search using the electronic bibliographic databases EMBASE (via Elsevier), MEDLINE (via Ovid) and Web of Science (via Clarivate Analytics), covering current records since 2000 (last update search: January 2024). This allowed us to assess the existing literature for the period in which public attention to patient safety has increased significantly and to capture possible trends through process improvements. To avoid publication bias, available grey literature through websites of relevant organisations [29–32] and study registries [33–37] were also searched. Secondly, the reference lists of eligible studies and related review articles were scanned.

#### Search strategy

For the search strategy, we combined search terms for each of ‘children’ (as patients), ‘drug-related problems’ (as exposure), and ‘hospital admissions’ (as outcome). Moreover, we narrowed the results by terms related to quantitative research. Generally, we applied indexing terms, free text terms and synonyms and did not restrict on language. (For the documentation of the search strategy, see Additional file 1).

#### Eligible studies

Eligible studies had to include paediatric data and had to report on the condition ‘drug-related hospital admissions’, providing data on its frequency, distribution, pattern, or determinants. To be comprehensive, we conceded heterogeneity: Regardless of their geographic location, we accepted studies with different definitions of ‘drug-related problems’ [24] and settings resulting from multiple clinical pathways for patients with drug-related problems, such as direct hospitalisations or subsequent to initial contacts with emergency departments and other health care providers. The different types of studies - retrospective or prospective, observational or experimental, longitudinal or cross-sectional - provide valuable data and were eligible. For experimental studies, only the results of the comparison group were considered. However, we excluded case series or spontaneous reports as we sought systematic and complete evidence for our evaluation. Abstracts were accepted if sufficient data were available, i.e., if it was possible to assess the inclusion and exclusion criteria. Additional file 2 shows the list of the predefined criteria.

#### Screening

After duplicates removal, the results of the database searches were first screened for relevance by one reviewer based on titles and abstracts. Independent screening of 10% of records confirmed the validity of single person screening for this step. The eligible full texts were independently evaluated for inclusion by two reviewers. Reasons for in- or exclusion were documented using the Eppi-Reviewer software [38]. In case of disagreement, a consensus was reached through discussion, involving a third reviewer if necessary.

### Data collection process

Data collection was performed independently and in duplicate by completing an Excel form. It covered study design (including organisational data, context, study population, definition of ‘drug-related problems’, and methodology), outcome data (including frequency of drug-related hospitalisations, involved drugs, data on the nature and preventability of drug-related problems), and quality characteristics of studies. If data were not available, this was also documented. Discrepancies were resolved through discussion.

### Critical appraisal

Critical appraisal was conducted independently by two reviewers using a tailor-made checklist, followed by a discussion round to reach consensus (a third reviewer was involved, if necessary). Our checklist (see Additional file 3) was based on the “Joanna Briggs Institute Critical Appraisal Checklist for prevalence studies” [21], which was modified according to other publications [6, 7, 39–41] for general applicability criteria, clarification and systematic grouping. Targeted questions and criteria were used to assess the studies in general for applicability, reporting problems and precision issues associated with the study designs. Moreover, we applied risk of bias criteria to assess selection bias, information bias regarding attrition, information bias regarding methods and measurements, and information bias regarding data analysis. Further, for the outcome ‘influencing factors’ we also considered questions about potential confounding factors. No studies or results were excluded, but applicability, quality and reliability were considered when interpreting the results.

### Analysis and synthesis of results

#### Study characteristics

Differences in the definition of ‘drug-related problems’ and in the methods of data collection were expected to have a major impact on the results of the study. Especially, studies using the concept of ‘adverse drug events’ [13, 24, 26] could capture more drug-related problems than those using the narrower concept of ‘adverse drug reactions’ [13, 27]. Furthermore, we expected that it is more likely to detect events with intensive monitoring (i.e., intensive review of records or assessment by study staff) than with routine data analysis (i.e., data from the routine care process without assessment by study staff, e.g., patient charts, discharge diagnoses, e-codes). Therefore, these characteristics were examined and studies were stratified according to them. Further attributes regarding design, population and methods of data collection were compiled in a table.

#### Frequency of drug-related hospitalisations

For the effect measure ‘frequency of drug-related hospitalisations’, the data on the number of patients with this event and the number of reference patients were taken from the studies. To obtain the results, the proportions were formed and the corresponding confidence intervals were calculated according to Clopper-Pearson [42]. In some publications the exact number of drug-related hospital admissions or the corresponding total number were not given, however, these data could be obtained by recalculations (e.g., recalculation of ‘all inpatients’ by the number and frequency of ‘cases with adverse drug reactions *during* hospitalisation’). For these results, we did not report a confidence interval, but marked them because the values may be less reliable e.g., due to rounding errors. In addition to stratification according to the concept of ‘drug-related problem’ used and the method of data collection, results were grouped: firstly, according to the type of reference population (‘inpatient stays’ or ‘emergency department visits’); and secondly, according to whether subsets were used as reference (e.g. only admissions with drugs or only acute admissions; if studies reported on both the entire reference group and a subset, both calculated results were presented as outcomes). We graphically displayed the results per group in forest plots ordered by frequency. Due to the clinical and methodological heterogeneity of the studies (evident in the visual inspection and statistical tests of the results (I^2^ > 80%)), and due to the high risk of bias, especially in routine studies, we refrained from reporting a pooled estimate (also for each of the subgroups). A narrative synthesis (qualitative and descriptive synthesis) of the frequency data was performed presenting results as median and interquartile range where applicable.

#### Nature of drug-related hospitalisations

The information on the drugs most commonly involved in drug-related hospital admissions was first summarised descriptively. Some studies provided a complete distribution pattern of the implicated drugs. We grouped these drugs according to their ATC (Anatomical Therapeutic Chemical Classification) codes [43] Level 1 (and 2) and rated the frequency of involvement.

To this end, we calculated the frequency of involvement of each drug class in relation to the total number of drugs involved in a study. We did not pool the percentages and subsequently test the group differences for statistical significance. Due to the heterogeneity of the studies, we would have had to control for numerous influencing factors which were, however, not documented.

All other findings, i.e., preventability, underlying factors, clinical manifestations, impact, and other influencing factors of drug-related hospitalisations, were summarised narratively.

#### General aspects

Funnel plot analysis to address the issue of publication bias was not performed. They would not be informative because asymmetric funnel plots could also be caused by the large methodological and clinical heterogeneity. Too few studies would be available for funnel plot analyses per subgroups.

For the analyses we used the software MicrosoftExcel**®**, meta in R (RStudio, version 2021.09.0) and Robvis [44].

## Results

### Literature search and study selection

**Fig. 1:**
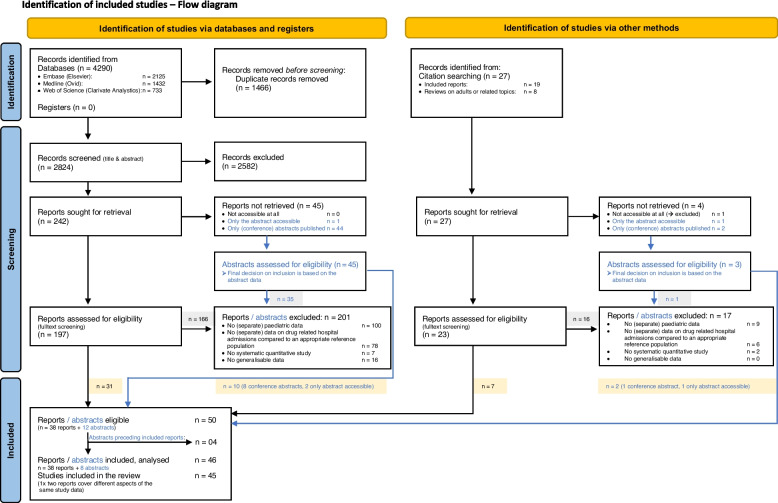
Flow diagram based on “PRISMA 2020 flow diagram for new systematic reviews which included searches of databases, registers and other sources” [22]. Abstracts for which no reports could be retrieved were also assessed for eligibility. Therefore, in addition to reports, eligible abstracts were also included. This additional information is shown in blue

Our database search returned a total of 4290 records. After duplicates were removed, 2824 records were screened on titles and abstracts. This resulted in 242 records for full text screening or in-depth abstract assessment (if only an abstract was available). Of these, 41 records were included in the review. In addition, citation search yielded a further 9 included records. In total, we thus obtained 50 reports or abstracts. As four conference abstracts preceded full-text publications, these abstracts were not required for our analyses. Consequently, our review comprised 46 publications containing data from 45 studies, as different aspects of one study were published in two reports (see flow diagram, Fig. 1) [45–90]. Reasons for exclusion of records were primarily due to missing (separate) data on children or on drug-related hospitalisations (drug-related problems during hospitalisation were often included instead). All records of the eligibility assessment together with the corresponding selection decisions are documented in the Additional file 4.

### Study characteristics

Categorisation according to the definition of ‘drug-related problems’ revealed that sixteen studies [45–60] were based on the broader concept of ‘adverse drug events’ [13, 24, 26], while 29 studies [61–90] focused on ‘adverse drug reactions’ [13, 27]. Although the studies could clearly be assigned to one of the two categories, the exact definitions still varied between the studies or were not given. Other concepts of drug-related problems were not used.

**Fig. 2:**
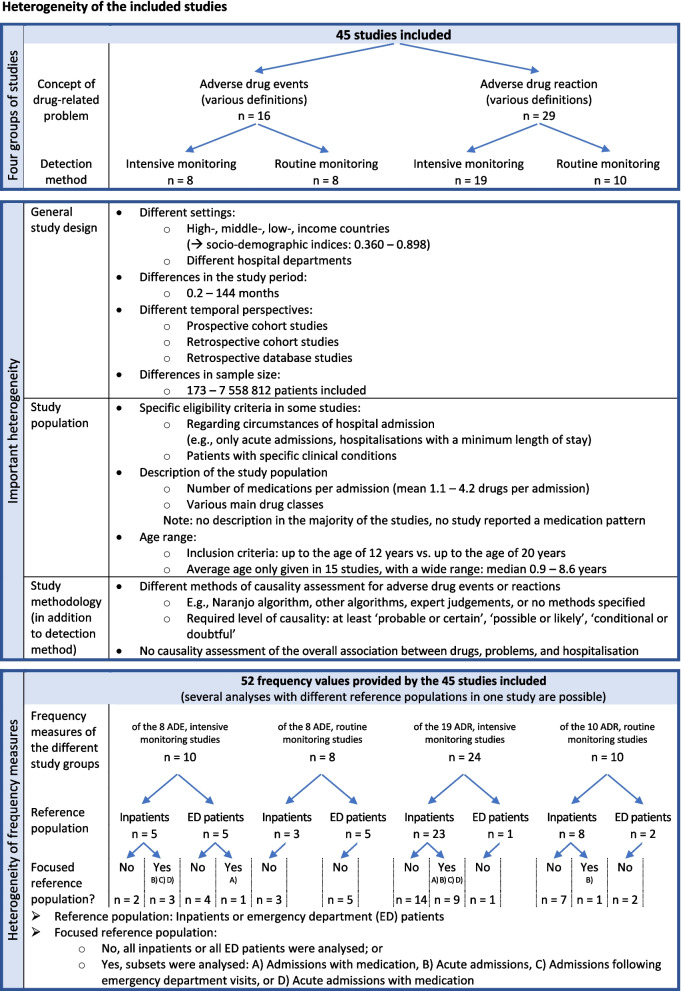
Overview of the heterogeneity found in the 45 studies included

Subsequent accounting for the second fundamental criterion, the ‘intensive’ or ‘routine’ monitoring methodology, resulted in the four groups of studies: a) Studies on ‘adverse drug events’, ‘with intensive monitoring’, b) Studies on ‘adverse drug events’, ‘based on routine monitoring’, c) Studies on ‘adverse drug reactions’, ‘with intensive monitoring’, and d) Studies on ‘adverse drug reactions’, ‘based on routine monitoring’ (see Fig. 2 and Additional file 5). Within each of these groups, there was further considerable heterogeneity. This concerned general study parameters, study populations as well as methods. Figure 2 provides an overview of the observed aspects of heterogeneity, Additional file 5 contains the detailed study characteristics and descriptive results.

### Critical appraisal

**Fig. 3:**
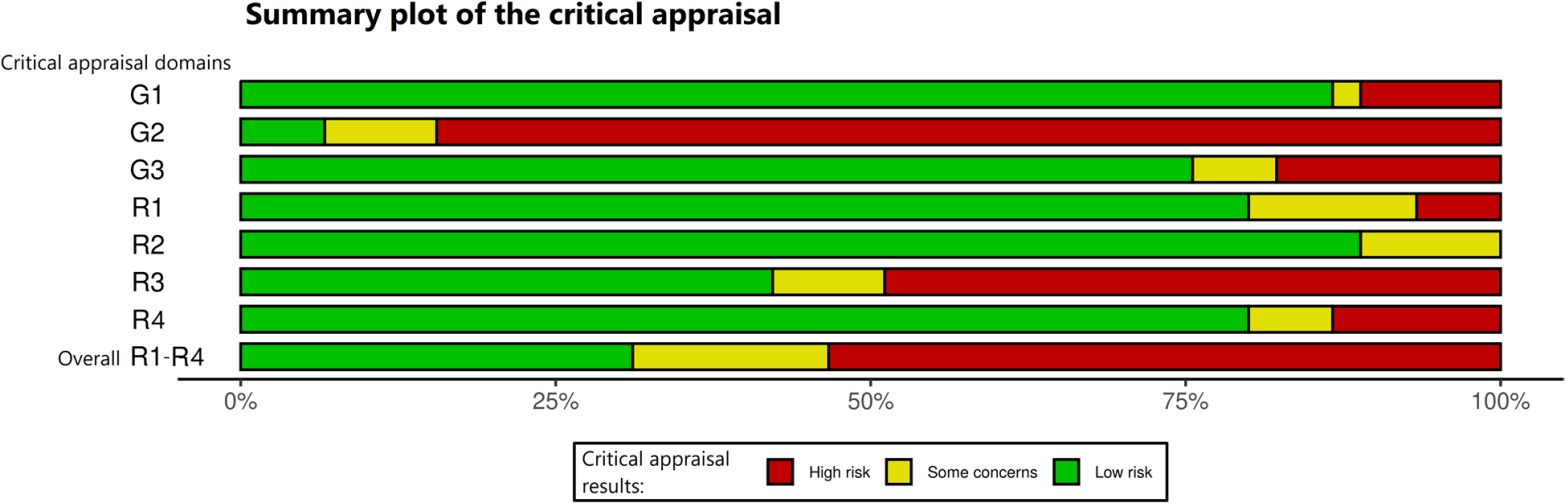
Summary of the critical appraisal. Review authors’ judgements about each critical appraisal domain presented as percentages across all included studies. Critical appraisal was conducted on 45 included studies using a checklist that covered domains on 3 general criteria and 4 risk of bias criteria. The results, ‘low concerns’, ‘some concerns’, and ‘high concerns about issues or risks of bias’, have been pooled based on equal weighting of each study for each domain. Critical appraisal domains: G1: General applicability, G2: General reporting issues, G3: General issues in precision, R1: Risk of selection bias, R2: Risk of information bias due to attrition, R3: Risk of information bias due to issues with methods and measurements, R4: Risk of information bias due to issues with data analysis, Overall: R1-4: Overall risk of bias appraisal

**Fig. 4:**
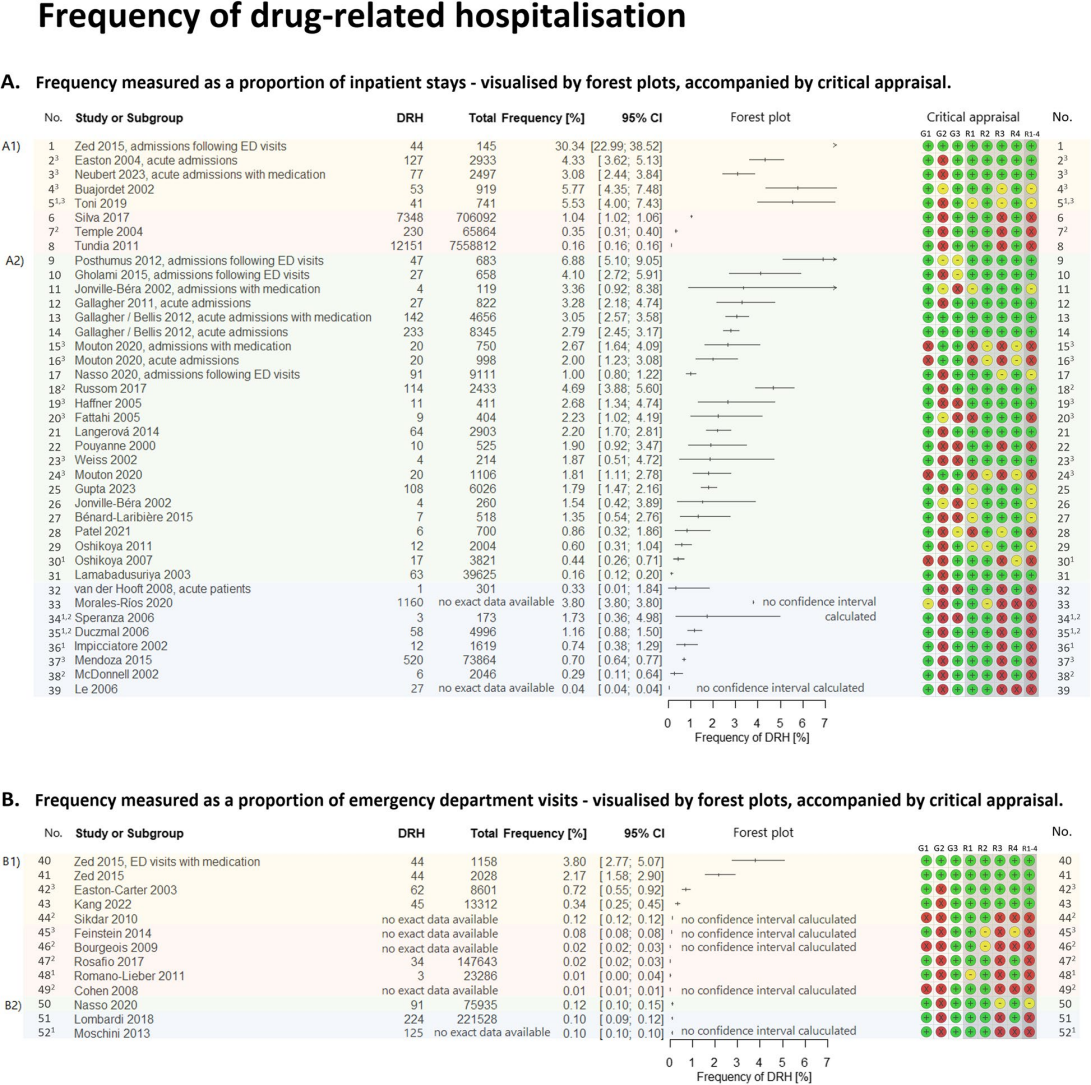
Frequency outcomes of drug-related hospitalisations, visualised by forest plots, accompanied by critical appraisal. Frequencies of DRHs as a proportion of inpatient stays in studies investigating ‘adverse drug events’ (A1) or ‘adverse drug reactions’ (A2). Frequencies of DRHs as a proportion of emergency department visits in studies investigating ‘adverse drug events’ (B1) or ‘adverse drug reactions’ (B2). Frequencies and 95% confidence intervals (Clopper-Pearson) were calculated from the data presented in the studies. It was refrained from reporting pooled estimates due to heterogeneity (based on visual inspection, statistical investigation of heterogeneity (I2 > 80%), too obviously different study designs and settings), and a high risk of bias, especially in routine studies. *Abbreviations: No. *Number of the frequency value, *DRH *Drug-related hospitalisation, *CI* Confidence interval, *ED *Emergency department. Stratification of the frequency values: No. 1–5, 40–44: frequency values of studies with a broader concept of ‘adverse drug events’, ‘with intensive monitoring’ (yellow background). No. 6–8, 45–49: frequency values of studies with a broader concept of ‘adverse drug events’, ‘based on routine monitoring’ (orange background). No. 9–31, 50: frequency values of studies with a narrower concept of ‘adverse drug reactions’, ‘with intensive monitoring’ (green background). No. 32–39, 51–52: frequency values of studies with a narrower concept of ‘adverse drug reactions’, ‘based on routine monitoring’ (blue background). Footnotes: Number^1^: just abstract available, Number^2^: study identified via citation search, Number^3^: study without oncology patients. Critical appraisal: Critical appraisal domains: G1: General applicability, G2: General reporting issues, G3: General issues in precision, R1: Risk of selection bias, R2: Risk of information bias due to attrition, R3: Risk of information bias due to issues with methods and measurements, R4: Risk of information bias due to issues with data analysis, Overall: R1-4: Overall risk of bias appraisal

We compiled the results of the critical appraisal in the summary plot (Fig. 3) and in ‘traffic light’ plots (see Fig. 4). The bar chart of the summary plot shows the distribution of critical appraisals based on equal weighting of the 45 studies and thus informs about the overall assessment of the studies. It highlights two major issues: Firstly, all 18 studies with ‘routine monitoring’ [53–60, 81–90] showed a lack of information on the characteristics of the study design, especially in the description of the study population. Thus, there are serious issues in the reporting. This problem was found also in 20 [46–48, 50–52, 61, 63–65, 67–70, 74–76, 78–80] of the remaining 27 studies with ‘intensive monitoring’ as the detection method. Secondly, in all studies with ‘routine monitoring’, we had to assume that there was a high risk of under-recording for the outcome ‘drug-related hospitalisations’. In these studies, the method and execution of the measurement led to a high risk of information bias. In the 27 studies with ‘intensive monitoring’, such a problem occurred in four studies [48, 61, 67, 77]. A detailed description of the results of the critical appraisal with a general overview of the traffic light plot can be found in the Additional file 6.

Publication bias could not be explored through formal tests such as funnel plots. Literature searches for grey literature on the websites of relevant organisations and study registries did not reveal any missing studies. However, the high proportion of included abstracts or of publications found only via citation search could mean that related studies were not published (as full texts) or not published in the usual journals. Therefore, there is a risk that studies have been missed. Nevertheless, no noticeable patterns in the frequencies of drug-related problems regarding effect sizes or confidence intervals were evident (see Fig. 4, abstracts are marked with footnote^‘1’^, citation-search publications with footnote^‘2’^). Therefore, we did not assume publication bias.

### Frequency of drug-related hospital admissions

**Fig. 5:**
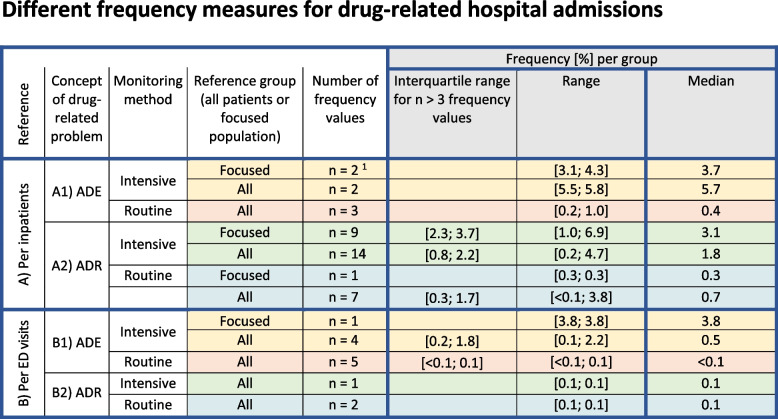
Frequency of drug-related hospitalisations for the different groups, i.e., considering the different concepts of drug-relating problems (ADE vs. ADR), the monitoring method (intensive vs. routine monitoring), and the reference populations (inpatients vs. emergency department (ED) visits, and if applicable focused populations (only admissions with medication, acute admissions, admissions following emergency department visits, or acute admissions with medication). The frequency [%] of drug-related hospital admissions per group is displayed as median, range and interquartile range if there are more than 3 frequency values in the group. The background colours indicate: yellow – studies with a broader concept of ‘adverse drug events’, ‘with intensive monitoring’; orange – studies with a broader concept of ‘adverse drug events’, ‘based on routine monitoring’; green – studies a narrower concept of ‘adverse drug reactions’, ‘with intensive monitoring’; blue – studies with a narrower concept of ‘adverse drug reactions’, ‘based on routine monitoring’. ^1^ Here, one outlier was excluded

The different frequency measures of drug-related hospital admissions are given in detail in Fig. 4 and as an overview in Fig. 5.

#### Reference population: inpatients

The forest plot, Fig. 4a, shows the relevant frequency data of drug-related hospital admissions from the perspective of inpatient care.

##### Studies with a broader concept of ‘adverse drug events’

Hospital admissions caused by adverse drug events detected by intensive monitoring were found in five studies [45, 47, 49, 51, 52] with frequencies ranging from 3.1% to 5.8% (median 4.9%, IQR [3.4; 5.7%], (excluding one outlier [49])). Focusing the analysis on admissions with medication and/or acute admissions did not show a large effect. In three studies [53, 56, 60] with routine monitoring, however, a significantly smaller frequency was found with 1.0%, 0.4% and 0.2%.

##### Studies with a narrower concept of ‘adverse drug reactions’

Similar findings with a bit lower frequencies were found for adverse drug reactions leading to hospitalisation: In studies [61–71, 73–80] with intensive monitoring, frequencies of 0.2% to 6.9% were reported; focusing to subsets (only admissions with medication or only acute admissions) increased the frequencies (median [62, 70, 71, 73, 76–78] 3.1%, IQR [2.3%; 3.7%] vs. median [61–69, 74, 75, 77, 79, 80] 1.8%, IQR [0.8%; 2.2%]). Again, studies with routine monitoring [81–86, 88, 90] indicated fewer hospital admissions related to adverse drug reactions, ranging from 0.04–3.8% (median 0.7%, IQR [0.3%; 1.6%]).

#### Reference population: emergency department patients

Figure 4b shows the perspective of emergency departments.

##### Studies with a broader concept of ‘adverse drug events’

In four studies [46, 48–50] with intensive monitoring, 2.2%, 0.7%, 0.3% and 0.1% of all patients in emergency departments required hospitalisation for adverse drug events. Again, five studies [54, 55, 57–59] that only analysed routine data reported significantly lower frequencies (median 0.02%, IQR [< 0.1%; 0.1%]).

##### Studies with a narrower concept of ‘adverse drug reactions’

In all three adverse drug reaction studies [78, 87, 89], regardless of monitoring mode, we found an admission rate of 0.1%.

The clinical, methodological and statistical heterogeneity found in the analysis of the studies argued against a pooled evaluation. Most of the assessed study characteristics could influence the number of drug-related hospitalisations identified either for logical reasons or as indicated in previous studies [6, 91, 92]. However, due to the multiple concurrent influences, we did not find any decisive patterns in the frequencies (e.g., depending on study year, study period, location, clinical conditions such as included oncology patients (see Fig. 4, studies without oncology patients are marked with footnote 4)). Not enough studies were available for meta-regression analyses.

### Nature of drug-related hospital admissions

Few studies provided data on the nature of drug-related hospital admissions in children or in the subgroup of children, respectively.

#### Drugs implicated in drug-related hospital admissions

**Fig. 6:**
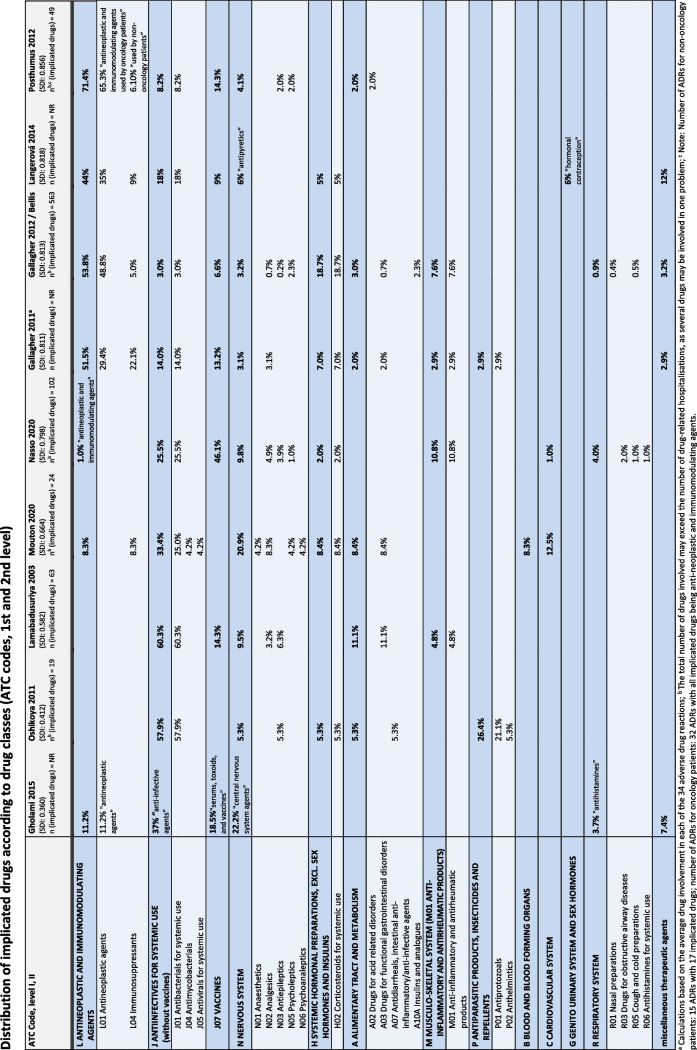
Detailed pattern of drugs implicated in drug-related hospital admissions. ATC codes (1st or 2nd level) were assigned to the drugs (drug classes) identified in nine studies and their relative frequencies were listed. Abbreviations: ATC code Anatomical Therapeutic Chemical (ATC) Classification, https://www.whocc.no/atc_ddd_index/, NR Not reported, Socio-Demographic Index, considered year = End of data collection, “Global Burden of Disease Study 2019” [93]

The 17 studies [45, 47, 51, 62, 65, 69–71, 73, 74, 76–78, 82, 85, 88, 90] investigating drugs involved identified anticancer drugs, anti-infectives, antiepileptics and analgesics as the most frequently implicated drug groups (see Additional file 7). The data from nine [65, 69–71, 73, 74, 76–78] of these studies could be used to examine the distribution among the individual drug classes in more detail, as they provided a detailed pattern on the involved drugs. The result of the in-depth analysis confirmed the high frequency of anti-cancer drugs, see Fig 6. This applies in particular to studies with a higher socio-demographic index [93]. In general, the utilisation of oncology drugs can also be expected to correlate with the socio-demographic index. However, as the proportion of patients receiving this class of drugs is known to be comparatively low in the usual study populations, this indicates a particular risk potential. Anti-infectives, vaccines, central nervous system drugs (especially antiepileptics and analgesics) and corticosteroids (systemic glucocorticoids) also ranked high. It should be noted that we had no data to adjust the calculated frequencies of involvement for drug utilisation. Therefore, it is not always possible to identify high-risk drugs from this analysis. Rather, from a public health point of view, these results show which drugs cause the most problems and could have a large impact on the population as a whole.

#### Clinical manifestations and impact

From the clinical point of view, the manifestation of the problems and the long-term sequelae are particularly relevant. However, there have been limited data on this. Haematological, skin, nervous system, respiratory and gastrointestinal reactions appeared to be prevalent, see Additional file 7. Some studies [46, 47, 49, 50, 59, 65, 66, 70, 71, 74, 76, 81, 86, 89] reported mild cases or no deaths. Others did not mention this outcome at all or reported on fatalities in combined evaluations (problems leading to hospitalisation and problems during hospitalisation) or in cross-age analyses. Thus, due to the risk of reporting bias, we could not indicate an overall result on long-term sequelae. In addition to the burden on individual patients, the impact on society is also important. In this context, four study authors addressed the direct costs incurred (Gallagher [73] (UK): mean cost per ADR admission: £4753 (95%CI £3439, £6066); Tundia [56] (US): mean cost per ADE admission USD3510; Oshikoya [69] (Nigeria): mean cost per moderate ADR admission: USD309.86 ± 31.09 or mean cost per serious ADR admission: USD1988.19 ± 414.76; Easton [47] (Australia): £100,707 per 127 drug-related hospital admissions (mean cost: £793)).

#### Underlying problems and preventability

Only the Easton 2004 study [47] contained information on the underlying causes of the drug problems. These included most frequently accidental or intentional poisoning, non-adherence and adverse drug reactions. Easton excluded problems due to accidental or deliberate poisoning and then determined a preventability of 46.9% using the methodology of Schumock and Thornton [94]. This was a comparatively high percentage. In total, preventability was investigated in eight studies [47, 51, 70, 71, 73, 76–78] using the method of Schumock and Thornton [94] or Hallas [95]. The median was 34.7% (IQR 23.4%; 42.0%), whereby problems of oncological patients were not evaluated, see Table 1. Only Gallagher et al. discussed the reasons for the results of the preventability assessment and concluded that more attentiveness to prescribing practices would be recommended [73].

**Table 1:** Available data on the preventability of drug-related hospitalisations

	**[%] Preventable DRHs **	**Method of measuring preventability**
Posthumus 2012 [71]	13.3%^a^	Schumock and Thornton^2^
Mouton 2020 [77]	22.5%	Schumock and Thornton^2^
Nasso 2020 [78]	24.2%	Schumock and Thornton^2^
Gallagher 2011 [70]	33.3%^c^	Hallas et al.^6^
Gallagher 2012 [73]	36.4%^a c d^	Hallas et al.^6^
Gholami 2015 [76]	37.1%	Schumock and Thornton^2^
Easton 2004 [47]	46.9%^b^	Schumock and Thornton^2^
Toni 2019 [51]	56.1%	NR

*Abbreviations: NR* Not reported, *DRH* Drug-related hospitalisation

^a^Preventable DRHs for non-oncology therapy-related DRHs

^b^Preventable DRHs other than accidental or intentional poisoning

^c^Classification: possibly or definitely preventable

^d^Additional information: preventable DRHs for oncology therapy-related DRHs: 6.7%, overall preventable DRHs: 22.%

#### Important influencing factors

Overall, we could not identify a clear pattern of influencing factors. Informative subgroup analyses or regression analyses were rarely conducted [69, 73, 74, 77, 81]. Significant results for an increased risk of drug-related hospital admissions were only reported for oncology patients and for patients with polypharmacy. Details and information on other influencing factors that were considered but are not conclusive are summarised in the Additional file 8.

## Discussion

Our systematic review confirmed that drug-related problems in paediatrics are a considerable public health issue of high interest. Overall, we identified 45 studies reporting data on drug-related hospitalisations in children between 2000 and 2024. These studies showed that, depending on the methodology (intensive or routine monitoring), between 3.1% to 5.8% and 0.2% to 1.0% of hospital admissions were due to adverse drug events. Studies focusing on adverse drug reactions found rates between 0.2% and 6.9% or between 0.04% and 3.8% of admissions (based on intensive monitoring or routine data, respectively). Lower rates were found among emergency department visits (0.01% to 3.8%).

These frequencies found are not substantially different from those found in reviews published 10 to 20 years ago, which reported drug-related hospital admissions ranging from 0.2 to 10.3% [6, 18, 19, 96]. Within the 24 years of our review, we have seen no trend. Overall, this suggests that the situation has not improved significantly in recent years. However, improvements in some aspects may also have been offset by an increase in problems in others, such as more complex drug therapies.

Findings on the nature of drug-related hospital admissions, although limited, were also consistent with previous reviews [6, 18, 19, 96]. For example, skin, nervous system, respiratory and gastrointestinal reactions were common clinical manifestations. Drugs implicated in drug-related hospital admissions often belonged to commonly used drug groups in paediatric outpatients: anti-infectives and analgesics. In addition, anti-epileptic drugs were often involved [97, 98]. There is a special situation for oncology drugs as cancer patients were not included in all studies. However, if oncological patients were part of a study population these drugs were often involved in drug-related hospitalisations [6].

### Clinical implications

Some of the studies included [47, 51, 70, 71, 73, 76–78] suggest, that a third of drug-related problems are preventable. Preventability was generally determined using established methods [94, 95] in studies with intensive monitoring (see Table 1). The result of the critical appraisal of these studies tended to be classified as low risk of bias, which underpins the reliability of this outcome. However, specific recommendations have rarely been given. Gallagher et al. [73] recommended more attentiveness when prescribing and Easton et al. [47] advised promoting patient adherence. Additionally, the disclosure of the frequency of drug-related hospitalisations could increase the motivation of all parties involved in the medication process to follow the known principles of medication safety. These include accounting for age-based dosing recommendations, considering all available evidence for off-label use and recognising clinically relevant drug interactions in the case of polypharmacy [12, 57, 99–101]. Furthermore, laboratory monitoring and prophylactic regimens for foreseeable adverse drug reactions should be used where reasonable and possible [102].

Studies reporting drug-related hospitalisations based on routine data reported significantly lower numbers than intensive monitoring studies. This points to the problem of under-identification during routine care and the need to raise awareness of drug-related problems. Therefore, methods similar to those used in intensive monitoring studies should be introduced into routine care, for example by involving hospital pharmacists or other specially trained personnel. Training should include the procedure of medication review and knowledge of the most relevant drugs and clinical manifestations. Additionally, algorithms to identify drug-related hospital admissions could enable faster identification [48, 64, 69, 71, 77, 103]. This would improve the efficiency of routine monitoring and reduce the cost of the procedure which is particularly important as financial and organisational requirements may otherwise discourage implementation.

### Insights on public health

As a measure of impact, it is useful to compare the ascertained frequency of drug-related hospitalisations among inpatients (0.04–6.9%) with the general leading causes of hospitalisation. According to data from a German study [104], about 7% of all children were hospitalised at least once in 2016. In this study, the most common reason for admission was tonsillitis (5.4%), followed by concussion (5.1%), gastroenteritis or colitis (4.4%) and acute bronchitis (3.3%). Drug problems would not be expected to appear in such an analysis. When they are routinely diagnosed, they are often not recorded as a principal diagnosis. Nevertheless, our review indicates that drug-related hospitalisations are among the most common reasons for hospitalisation in paediatrics.

The economic burden reported in some studies [47, 56, 69, 73] could not be used for a general quantification [105]. However, it is clear that additional hospitalisations result in additional costs that should not be underestimated.

### Implication for future research

In evaluating the studies, we were confronted with three main problems: the (clinical, methodological, and statistical) heterogeneity of the studies, the limited depth of information, and the high risk of information bias. For future research, it would be important to optimise study designs. Although clinical heterogeneity cannot be overcome, it could be better managed through enhanced reporting. More descriptive patient data, as recommended in general reporting guidelines for observational studies such as STROBE [106], could improve the assessment of outcomes. Regarding methodological heterogeneity, Wallerstedt et al. [91], reviewed in depth the methodological issues in assessing causality of drug-related admissions. We support their recommendation to explicitly assess the relationships between drug, problems and admissions. Common definitions of drug problems should be more strictly adhered to. A good classification of drug-related problems allows for clear conclusions about the underlying factors and how to avoid them. For example, the classification of the Pharmaceutical Care Network Europe (PCNE) offers a such a framework [24].

Despite the methodological challenges, we further acknowledge the benefits of routine data assessments as a complementary approach to intensive monitoring studies, as they provide insights into how drug-related problems are recognised in daily clinical practice. However, for an accurate determination of the extent of drug-related hospital admissions, the identification of drug-related problems in routine care needs to be improved to reduce the risk of information bias.

### Strengths and limitations

For our systematic review, we applied a rigorous approach to collect and analyse all available evidence since the year 2000. We considered a wide range of concepts that explored drug-related hospital admissions in different ways. We have evaluated them thoroughly. As highlighted, heterogeneity was an important issue. We carefully identified differences in study design, study population, setting and study methodology. Due to important influencing factors (i.e., differences in concepts of drug-related problems, detection methods and reference population), we had to conduct separate analyses and refrain from pooled estimates. Nevertheless, this avoided misleading syntheses. We cannot exclude the possibility that publications may have been missed because of publication policies in this area of research. Some study results were found to be available only in abstracts. However, after evaluating our search strategy and possible publication bias, we concluded that our approach was robust.

## Conclusion

Drug-related problems leading to hospitalisation in paediatrics are highly relevant and need further attention. Studies worldwide have reported a high proportion of drug-related hospital admissions. Due to heterogeneity and methodological challenges, it is not possible to summarise and quantify the exact magnitude of the problem in a pooled estimate. Compared to studies with intensive monitoring, studies with routine data reveal under-identification in clinical practice. We therefore need more and better documentation in studies to improve applicability and generalisability. This would also be a prerequisite for deriving more precise recommendations. In conclusion, we need more systematic research and more attention in clinical practice.

### Supplementary Information

**Additional file 1.** Documentation of the search strategy.

**Additional file 2.** Full text screening: In- and exclusion criteria.

**Additional file 3.** Critical appraisal criteria with description.

**Additional file 4.** Documentation: Results of full text screening.

**Additional file 5.** Study characteristics and descriptive results.

**Additional file 6.** Critical appraisal results details.

** Additional file 7.** Drugs implicated and clinical manifestations.

** Additional file 8. ** Details on influencing factors.

## Data Availability

No datasets were generated or analysed during the current study.

## Abbreviations

ADE: Adverse drug event
ADR: Adverse drug reaction
ATC: Anatomical Therapeutic Chemical (ATC) Classification
CI: Confidence interval
DRH: Drug-related hospitalisation
ED: Emergency department
IQR: Interquartile range
NR: Not reported
PCNE: Pharmaceutical Care Network Europe
PRISMA: Preferred Reporting Items for Systematic Reviews and Meta-Analysis
