# Supplementary material for: The epidemiology of drug-related hospital admissions in paediatrics – a systematic review
Source: Arch Public Health. 2024 Jun 4;82:81. doi: 10.1186/s13690-024-01295-4 (PMC11149243; doi:10.1186/s13690-024-01295-4)
Supplement: Supplementary file 1 — Additional file 1. Documentation of the search strategy. [file 13690_2024_1295_MOESM1_ESM.pdf]

The epidemiology of drug-related hospital admissions in paediatrics – a systematic review

Documentation of the search strategy

Research question:
What is the incidence and nature of drug-related hospital admissions in paediatrics?

- Which drugs are involved?
- What kind of problems lead to hospital admissions? – which of these problems would be preventable?
- Which are additional important influencing factors (determinants) leading to drug-related hospital admissions in paediatrics?

Occurrence relation:
Paediatric hospital admissions as a function of problems related to medication, conditional on extraneous determinants such as patient or health care characteristics.
Domain:
Children

Search terms

PICO:		Terms for …
**Patients**	children	children
**Intervention or exposure**	drug related problems	drug AND
		related problem
**Control**	no drug related problem	*(not relevant for the search)*
**Outcome**	hospital admission	hospital AND
		admission
**Type of study**	studies including quantitative research	quantitative research

Searches

Database Name	Platform	Date Coverage	Date of Search	# of results
EMBASE	ELSEVIER	2000 – present	2021-11-03	1594
MEDLINE	OVID	2000 – present	2021-11-03	1051
Web of Science	Clarivate Analytics	2000 – present	2021-11-02	562
EMBASE	ELSEVIER	2021-11-04 – present	2022-05-12	162
MEDLINE	OVID	2000 – present → deduplication with initial search	2022-05-12	113
Web of Science	Clarivate Analytics	2021-11-03 to present	2022-05-12	78
EMBASE	ELSEVIER	2022-05-01 – present	2024-01-29	369
MEDLINE	OVID	2022-01-01 – present	2024-01-22	268
Web of Science	Clarivate Analytics	2022-05-01 – present	2024-01-22	93

Search terms

Embase (via Elsevier)

ADVANCED search	
#1 „CHILDREN“	(infan* OR newborn* OR 'new born*' OR perinat* OR neonat* OR baby* OR babies OR toddler* OR child* OR schoolchild* OR adolescen* OR juvenil* OR youth* OR teen* OR pubescen* OR pediatric* OR paediatric* OR prematur* OR preterm*<br>OR<br>'pediatrics'/exp/mj OR 'adolescent'/exp/mj OR 'child'/exp/mj OR 'hospitalized adolescent'/exp/mj OR 'hospitalized child'/exp/mj OR 'hospitalized infant'/exp/mj)
#2<br><br>“DRUG RELATED PROBLEM”<br><br>NEAR/50<br><br>#HOSPITAL ADMISSION”	(((drug* OR medication* OR medicine* OR pharmacotherap* OR 'drug therap*') NEAR/50 (morbidity OR toxicity OR 'adverse event*' OR 'side effect*' OR 'adverse effect*' OR 'toxic effect*' OR 'adverse outcome*' OR 'adverse reaction*') NEAR/50 ('hospital admission*' OR 'inpatient admission*' OR 'hospital referral*' OR 'inpatient referral*' OR 'hospitalisation*' OR 'hospitalization*' OR 'hospitalised*' OR 'hospitalized*' OR 'hospital stay'))<br>OR<br>((drug* OR medication* OR medicine* OR pharmacotherap* OR 'drug therap*') NEAR/50 (morbidity OR toxicity OR 'adverse event*' OR 'side effect*' OR 'adverse effect*' OR 'toxic effect*' OR 'adverse outcome*' OR 'adverse reaction*') NEAR/50 (hospital* OR inpatient*) NEAR/50 ('admitted to' OR 'referred to'))<br>OR<br>(('drug related problem*' OR 'medication related problem*' OR 'adverse drug event*' OR 'adverse drug effect*' OR 'adverse drug reaction*' OR 'medication error*' OR 'prescribing error*' OR 'prescription error*' OR 'drug error*') NEAR/50 ('hospital admission*' OR 'inpatient admission*' OR 'hospital referral*' OR 'inpatient referral*' OR 'hospitalisation*' OR 'hospitalization*' OR 'hospitalised*' OR 'hospitalized*' OR 'hospital stay'))<br>OR<br>(('drug related problem*' OR 'medication related problem*' OR 'adverse drug event*' OR 'adverse drug effect*' OR 'adverse drug reaction*' OR 'medication error*' OR 'prescribing error*' OR 'prescription error*' OR 'drug error*') NEAR/50 (hospital* OR inpatient*) NEAR/50 ('admitted to' OR 'referred to')))
#3<br><br>“DRUG RELATED PROBLEM (Abbreviations)”<br><br>NEAR/50<br><br>#HOSPITAL ADMISSION”	(('adr' OR 'ade' OR 'me' OR 'adrs' OR 'ades' OR 'mes') NEAR/50 ('hospital admission*' OR 'inpatient admission*' OR 'hospital referral*' OR 'inpatient referral*' OR 'hospitalisation*' OR 'hospitalization*' OR 'hospitalised*' OR 'hospitalized*' OR 'hospital stay'))<br>OR<br>(('adr' OR 'ade' OR 'me' OR 'adrs' OR 'ades' OR 'mes') NEAR/50 (hospital* OR inpatient*) NEAR/50 ('admitted to' OR 'referred to'))
#4<br>precise comprehensive terms for “drug related hospital admissions”	'drug related hospital admission*' OR 'drug related hospitalisation*' OR 'drug related hospitalization*' OR 'medication related hospital admission*' OR 'medication related hospitalisation*' OR 'medication related hospitalization*' OR (('adverse drug reaction'/exp/mj OR 'side effect'/exp/mj OR 'side effect assessment'/exp/mj) AND ('hospital admission'/exp/mj OR 'hospitalization'/exp/mj OR 'hospital readmission'/exp/mj OR 'child hospitalization'/exp/mj))
#5<br>„studies including quantitative research“	incidence*:ti,ab,kw OR prevalence*:ti,ab,kw OR occurence:ti,ab,kw OR risk*:ti,ab,kw OR frequenc*:ti,ab,kw OR epidemiolog*:ti,ab,kw OR 'incidence'/exp/mj OR 'prevalence'/exp/mj OR 'epidemiological data'/exp/mj OR 'epidemiology'/exp/mj
#1 AND (#2 OR #3 OR #4) AND #5 AND [2000-2022]/py	

Medline (via Ovid)

Resource: Ovid MEDLINE(R) and Epub Ahead of Print, In-Process, In-Data-Review & Other Non-Indexed Citations and Daily<br>“Search fields”: All Fields	
#1 „CHILDREN“	(infan* or newborn* or new-born* or perinat* or neonat* or baby* or babies or toddler* or child* or schoolchild* or adolescen* or juvenil* or youth* or teen* or pubescen* or pediatric* or paediatric* or prematur* or preterm*).af.<br>or<br>Pediatrics/ or Adolescent/ or Child/ or Infant/
#2<br><br>“DRUG RELATED PROBLEM”<br><br>NEAR/50<br><br>#HOSPITAL ADMISSION”	(((drug* or medication* or medicine* or pharmacotherap* or 'drug therap*') adj50 (morbidity or toxicity or 'adverse event*' or 'side effect*' or 'adverse effect*' or 'toxic effect*' or 'adverse outcome*' or 'adverse reaction*') adj50 ('hospital admission*' or 'inpatient admission*' or 'hospital referral*' or 'inpatient referral*' or 'hospitalisation*' or 'hospitalization*' or 'hospitalised*' or 'hospitalized*' or 'hospital stay'))<br>or<br>((drug* or medication* or medicine* or pharmacotherap* or 'drug therap*') adj50 (morbidity or toxicity or 'adverse event*' or 'side effect*' or 'adverse effect*' or 'toxic effect*' or 'adverse outcome*' or 'adverse reaction*') adj50 (hospital* or inpatient*) adj50 ('admitted to' or 'referred to'))<br>or<br>(('drug related problem*' or 'medication related problem*' or 'adverse drug event*' or 'adverse drug effect*' or 'adverse drug reaction*' or 'medication error*' or 'prescribing error*' or 'prescription error*' or 'drug error*') adj50 ('hospital admission*' or 'inpatient admission*' or 'hospital referral*' or 'inpatient referral*' or 'hospitalisation*' or 'hospitalization*' or 'hospitalised*' or 'hospitalized*' or 'hospital stay'))<br>or<br>(('drug related problem*' or 'medication related problem*' or 'adverse drug event*' or 'adverse drug effect*' or 'adverse drug reaction*' or 'medication error*' or 'prescribing error*' or 'prescription error*' or 'drug error*') adj50 (hospital* or inpatient*) adj50 ('admitted to' or 'referred to'))).af.
#3<br><br>“DRUG RELATED PROBLEM (Abbreviations)”<br><br>NEAR/50<br><br>#HOSPITAL ADMISSION”	((('ADR' or 'ADE' or 'ME' or 'ADRs' or 'ADEs' or 'MEs') adj50 (hospital* or inpatient*) adj50 ('admitted to' or 'referred to'))<br>or<br>(('ADR' or 'ADE' or 'ME' or 'ADRs' or 'ADEs' or 'MEs') adj50 ('hospital admission*' or 'inpatient admission*' or 'hospital referral*' or 'inpatient referral*' or 'hospitalisation*' or 'hospitalization*' or 'hospitalised*' or 'hospitalized*' or 'hospital stay'))).af.
#4<br>precise comprehensive terms for “drug related hospital admissions”	('drug related hospital admission*' or 'drug related hospitalisation*' or 'drug related hospitalization*' or 'medication related hospital admission*' or 'medication related hospitalisation*' or 'medication related hospitalization*').af. or ((*Patient Admission/ or *Patient Readmission/) and (*"Drug-Related Side Effects and Adverse Reactions"/ or *Medication Errors/))
#5<br>„studies including quantitative research“	(incidence* or prevalence* or occurence or risk* or frequenc* or epidemiolog*).ab,kf,ti. or *Incidence/ or *Prevalence/ or *Epidemiology/ or Epidemiology/
#6<br>1 and (2 or 3 or 4) and 5<br>#7 limitation of the period<br>6 and 2000:2022.(sa_year).	
((infan* or newborn* or new-born* or perinat* or neonat* or baby* or babies or toddler* or child* or schoolchild* or adolescen* or juvenil* or youth* or teen* or pubescen* or pediatric* or paediatric* or prematur* or preterm* or Pediatrics or Adolescent or Child or Infant) and (((drug* or medication* or medicine* or pharmacotherap* or 'drug therap*') adj50 (morbidity or toxicity or 'adverse event*' or 'side effect*' or 'adverse effect*' or 'toxic effect*' or 'adverse outcome*' or 'adverse reaction*') adj50 ('hospital admission*' or 'inpatient admission*' or 'hospital referral*' or 'inpatient referral*' or 'hospitalisation*' or 'hospitalization*' or 'hospitalised*' or 'hospitalized*' or 'hospital stay')) or ((drug* or medication* or medicine* or pharmacotherap* or 'drug therap*') adj50 (morbidity or toxicity or 'adverse event*' or 'side effect*' or 'adverse effect*' or 'toxic effect*' or 'adverse outcome*' or 'adverse reaction*') adj50 (hospital* or inpatient*) adj50 ('admitted to' or 'referred to')) or (('drug related problem*' or 'medication related problem*' or 'adverse drug event*' or 'adverse drug effect*' or 'adverse drug reaction*' or 'medication error*' or 'prescribing error*' or 'prescription error*' or 'drug error*') adj50 ('hospital admission*' or 'inpatient admission*' or 'hospital referral*' or 'inpatient referral*' or 'hospitalisation*' or 'hospitalization*' or 'hospitalised*' or 'hospitalized*' or 'hospital stay')) or (('drug related problem*' or 'medication related problem*' or 'adverse drug event*' or 'adverse drug effect*' or 'adverse drug reaction*' or 'medication error*' or 'prescribing error*' or 'prescription error*' or 'drug error*') adj50 (hospital* or inpatient*) adj50 ('admitted to' or 'referred to')) or ((('ADR' or 'ADE' or 'ME' or 'ADRs' or 'ADEs' or 'MEs') adj50 (hospital* or inpatient*) adj50 ('admitted to' or 'referred to')) or (('ADR' or 'ADE' or 'ME' or 'ADRs' or 'ADEs' or 'MEs') adj50 ('hospital admission*' or 'inpatient admission*' or 'hospital referral*' or 'inpatient referral*' or 'hospitalisation*' or 'hospitalization*' or 'hospitalised*' or 'hospitalized*' or 'hospital stay'))) or ('drug related hospital admission*' or 'drug related hospitalisation*' or 'drug related hospitalization*' or 'medication related hospital admission*' or 'medication related hospitalisation*' or 'medication related hospitalization*' or ((Patient Admission or Patient Readmission) and ("Drug-Related Side Effects and Adverse Reactions" or Medication Errors)))) and (incidence* or prevalence* or occurence or risk* or frequenc* or epidemiolog* or Incidence or Prevalence or Epidemiology or Epidemiology)).af.	

Web of Science (Clarivate Analystics)

Web of Science Core Collection		
#1 „CHILDREN“		Basic search:<br>All Fields, date: 2000-01-01 – 2022-12-31<br><br>(infan* OR newborn* OR new-born* OR perinat* OR neonat* OR baby* OR babies OR toddler* OR child* OR schoolchild* OR adolescen* OR juvenil* OR youth* OR teen* OR pubescen* OR pediatric* OR paediatric* OR prematur* OR preterm*)
#6<br><br>“DRUG RELATED PROBLEM”<br><br>NEAR/50<br><br>#HOSPITAL ADMISSION”		Basic search:<br>Topic, date: 2000-01-01 – 2022-12-31
	#2	((drug* OR medication* OR medicine* OR pharmacotherap* OR "drug therap*") NEAR/50 (morbidity OR toxicity OR "adverse event*" OR "side effect*" OR "adverse effect*" OR "toxic effect*" OR "adverse outcome*" OR "adverse reaction*") NEAR/50 ("hospital admission*" OR "inpatient admission*" OR "hospital referral*" OR "inpatient referral*" OR "hospitalisation*" OR "hospitalization*" OR "hospitalised*" OR "hospitalized*" OR "hospital stay"))
	#3	((drug* OR medication* OR medicine* OR pharmacotherap* OR "drug therap*") NEAR/50 (morbidity OR toxicity OR "adverse event*" OR "side effect*" OR "adverse effect*" OR "toxic effect*" OR "adverse outcome*" OR "adverse reaction*") NEAR/50 (hospital* OR inpatient*) NEAR/50 ("admitted to" OR "referred to"))
	#4	(("drug related problem*" OR "medication related problem*" OR "adverse drug event*" OR "adverse drug effect*" OR "adverse drug reaction*" OR "medication error*" OR "prescribing error*" OR "prescription error*" OR "drug error*") NEAR/50 ("hospital admission*" OR "inpatient admission*" OR "hospital referral*" OR "inpatient referral*" OR "hospitalisation*" OR "hospitalization*" OR "hospitalised*" OR "hospitalized*" OR "hospital stay"))
	#5	(("drug related problem*" OR "medication related problem*" OR "adverse drug event*" OR "adverse drug effect*" OR "adverse drug reaction*" OR "medication error*" OR "prescribing error*" OR "prescription error*" OR "drug error*") NEAR/50 (hospital* OR inpatient*) NEAR/50 ("admitted to" OR "referred to"))
		#2 OR #3 OR #4 OR #5
#9<br><br>“DRUG RELATED PROBLEM (Abbreviations)”<br><br>NEAR/50<br><br>#HOSPITAL ADMISSION”		Basic search:<br>Topic, date: 2000-01-01 – 2023-12-31
	#7	(("ADRs" OR "ADEs" OR "MEs" OR "ADR" OR "ADE" OR "ME") NEAR/50 ("hospital admission*" OR "inpatient admission*" OR "hospital referral*" OR "inpatient referral*" OR "hospitalisation*" OR "hospitalization*" OR "hospitalised*" OR "hospitalized*" OR "hospital stay"))
	#8	(("ADRs" OR "ADEs" OR "MEs" OR "ADR" OR "ADE" OR "ME") NEAR/50 (hospital* OR inpatient*) NEAR/50 ("admitted to" OR "referred to"))
		#7 OR #8
#10<br>precise comprehensive terms for “drug related hospital admissions”		Basic search:<br>All Fields, date: 2000-01-01 – 2022-12-31<br><br>("drug related hospital admission*" OR "drug related hospitalisation*" OR "drug related hospitalization*" OR "medication related hospital admission*" OR "medication related hospitalisation*" OR "medication related hospitalization*")
#11<br>„studies including quantitative research“		Basic search:<br>Topic, date: 2000-01-01 – 2022-12-31<br><br>(incidence* OR prevalence* OR occurence OR risk* OR frequency OR epidemiolog*)
#1 AND (#6 OR #9 OR #10) AND #11		
