# Supplementary material for: The epidemiology of drug-related hospital admissions in paediatrics – a systematic review
Source: Arch Public Health. 2024 Jun 4;82:81. doi: 10.1186/s13690-024-01295-4 (PMC11149243; doi:10.1186/s13690-024-01295-4)
Supplement: Supplementary file 2 — Additional file 2. Full text screening: In- and exclusion criteria. [file 13690_2024_1295_MOESM2_ESM.pdf]

The epidemiology of drug-related hospital admissions in paediatrics – a systematic review

Screening - full texts: Inclusion / exclusion criteria

Inclusion criteria, description:
Topic: Studies with information on the epidemiology of drug-related hospitalisation of children (frequency, distribution, patterns, and determinants of paediatric hospitalisation associated with drug related problems)

- Exposure: "Drug related problems"
- Population: Studies including paediatric patients < 18 years
- Outcome: Hospitalisation
- Types of study: Systematic quantitative studies → retrospective or prospective studies, observational or experimental studies, studies with longitudinal design or cross-sectional design (e.g., cohort studies, case-control studies, or before-after studies)
- Context: Different study sites are possible: (paediatric) hospitals, emergency departments or other hospital referrers

Exclusion criteria, description:

- Exposure / Outcome: Drug related problems during hospital stay
- Population: Studies on other age groups or that do not report separate paediatric data
- Types of studies: Studies without systematic examination of the relationship between hospital admission and drug-related problems, (e.g. only spontaneous or voluntary reporting methods, case reports or case series).

Screen on full texts: Codes

	Code	Criteria
"Include"	INCLUDE	• Study population:<br>○ patients < 18 years included<br>○ separate paediatric data are available<br>• Study examines drug-related hospital admissions. (exposure: "Drug-related problem": In the context of drug therapy, exposure to a systemic or topical drug caused any clinical adverse event or therapy problem.)<br>• Study informs about drug-related hospital admissions<br>○ "Hospitalisation as a consequence": Hospital admissions with a causal relationship to drug-related problems were measured.<br>○ includes information on incidence or nature.<br>• Study type:<br>○ systematic quantitative study<br>○ retrospective or prospective studies / observational or experimental studies / studies with longitudinal design or cross-sectional design (e.g., cohort studies, case-control studies, or before-after studies).<br>○ studies without a peer-review process (e.g., conference abstracts, letters to editors, correspondence, editorials, discussion papers): If the necessary original data are presented include, but mark --> exclusion from analysis will depend on quality assessment<br>• Study site: (paediatric) hospitals, emergency departments or other hospital referrers, or health care facilities
"Exclude": (hierarchical decisions):	EXCLUDE on study population<br>(Topic would match (or is related), but study population does not match	• Not a paediatric study population (under 18 years)<br>• No paediatric study population included.<br>• Paediatric data not separately reported<br>• Study explicitly excludes paediatric patients below 18 years of age (i.e. study that looks at other age groups).<br>• Study on general population does not report separate paediatric data.<br>(<--> Eligible study population: patients < 18 years (at least separate paediatric data must be available)).
	Exclude: Exclude on topic: Exposition<br>(Topic / study population would match, but exposition does not match)	• Study does not examine drug-related problems as cause of hospital admissions (e.g., drug-related problems during hospitalisation).<br>• No standardised monitoring of drug-related problems leading to hospitalisation<br>• No causal relationship between drug and problem
	Exclude: Exclude on topic: Outcome<br>(Topic / study population / exposition would match, but study outcome does not match)	• Study does not inform about hospital admissions due to drug related problems, i.e., it does not include information on incidence or nature of drug related hospital admissions.<br>• there is no defined study population at risk ("no reference")
	EXCLUDE on study type<br>(Topic / study population / exposition /outcome would match, but type of study does not match)	• Study does not systematically examine the relationship between hospital admission and drug-related problems, e.g.,<br>○ only spontaneous or voluntary reporting methods<br>○ case reports or case series.<br>• It is another type of study/ publication<br>○ systematic review<br>○ literature / scoping review<br>○ special citation e.g. an editorial without sufficient information.<br>• (The research question of the review could be answered by retrospective or prospective studies, observational or experimental studies, studies with longitudinal design or cross-sectional design (e.g., cohort studies, case-control studies, or before-after studies). Therefore, provided they are systematic quantitative studies, there are no restrictions on the types of study design eligible for inclusion.)
	"Special cases"	• Inclusion criteria fulfilled, but – just special population / conditions / special manifestations → no generalisable data → flag these studies, studies should only be included in special analyses if possible.
