# Supplementary material for: The epidemiology of drug-related hospital admissions in paediatrics – a systematic review
Source: Arch Public Health. 2024 Jun 4;82:81. doi: 10.1186/s13690-024-01295-4 (PMC11149243; doi:10.1186/s13690-024-01295-4)
Supplement: Supplementary file 3 — Additional file 3. Critical appraisal criteria with description. [file 13690_2024_1295_MOESM3_ESM.pdf]

The epidemiology of drug-related hospital admissions in paediatrics – a systematic review

Critical appraisal – checklist

The checklist of JBI CRITICAL APPRAISAL CHECKLIST FOR STUDIES REPORTING PREVALENCE DATA[1] and useful criteria of Smyth 2012[2] and Ghaleb 2006[3] were adopted. The risk of bias categories are according to Faillie 2017[4] / Cochrane Collaboration's tool for assessing risk of bias[5,6].

(See colors for the origin of the criteria (in black further clarifications).)

[1]Checklist_for_Prevalence_Studies.pdf. Accessed May 31, 2021. https://jbi.global/critical-appraisal-tools

[2]Smyth, Rebecca Mary Diane, Elizabeth Gargon, Jamie Kirkham, Lynne Cresswell, Su Golder, Rosalind Smyth, and Paula Williamson. "Adverse Drug Reactions in Children—A Systematic Review." Edited by Joseph S. Ross. *PLoS ONE* 7, no. 3 (March 5, 2012): e24061. https://doi.org/10.1371/journal.pone.0024061.

[3]Ghaleb, Maisoon Abdullah, Nick Barber, Bryony D Franklin, Vincent WS Yeung, Zahra F Khaki, and Ian CK Wong. "Systematic Review of Medication Errors in Pediatric Patients." *Annals of Pharmacotherapy* 40, no. 10 (October 2006): 1766–76. https://doi.org/10.1345/aph.1G717.

[4]Faillie, Jean-Luc, Pili Ferrer, Amandine Gouverneur, Damien Driot, Shoma Berkemeyer, Xavier Vidal, Maria José Martínez-Zapata, et al. "A New Risk of Bias Checklist Applicable to Randomized Trials, Observational Studies, and Systematic Reviews Was Developed and Validated to Be Used for Systematic Reviews Focusing on Drug Adverse Events." *Journal of Clinical Epidemiology* 86 (June 2017): 168–75. https://doi.org/10.1016/j.jclinepi.2017.04.023.

[5]Higgins, J. P. T., D. G. Altman, P. C. Gotzsche, P. Juni, D. Moher, A. D. Oxman, J. Savovic, et al. "The Cochrane Collaboration's Tool for Assessing Risk of Bias in Randomised Trials." *BMJ* 343, no. oct18 2 (October 18, 2011): d5928–d5928. https://doi.org/10.1136/bmj.d5928.

[6]Higgins JPT, Thomas J, Chandler J, Cumpston M, Li T, Page MJ, Welch VA (editors). *Cochrane Handbook for Systematic Reviews of Interventions* version 6.3 (updated February 2022). Cochrane, 2022. Available from www.training.cochrane.org/handbook.

Possible answers:

**General applicability / Further general quality criteria:**	**Risk of bias criteria:**
Yes<br>Probably (mainly) yes<br>Probably (mainly) no<br>No<br>Unclear<br>Not applicable	Yes<br>Probably yes<br>Probably no<br>No<br>Unclear<br>Not applicable

	**Description where relevant**	**Overall appraisal** (select the highest applying category)
**G1. General applicability:** study design and objectives		
Were the aims/ objectives of the study clearly stated and appropriate?	Appropriate: Are the objectives matching the research question of the review --> appropriateness to answer the research question of the review?<br>*(primary research question → yes, secondary aim → probably yes)*	• 1x yes + 1x (probably) yes = low risk<br>• ≥2x probably yes = some concerns<br>• 1x yes + 1x probably no = some concerns<br>• 1x probably yes + 1x probably no = high risk<br>• >1x (probably) no = high risk<br>• 1x (probably) yes + 1x no = high risk
Was the study design clearly specified and appropriate? (Smyth / Faillie)	In an appropriate study design, patients with drug-related-hospitalisations must be identified from a baseline population (--> frequency data) and - if additionally available - characterised (--> data on the nature of DRH)).<br>*(no information on the design or it was completely inappropriate- → no*<br>*combination of different study designs → probably no*<br>*proportion of DRH was not clearly reported or could not be clearly calculated, or outcome is rarely informative → probably no*<br>*little information, but the design is still recognisable, and if it is plausible that the design is appropriate → probably yes)*	
**G2, 3 Further general quality criteria:**		
**G2 Reporting issues:** Problems of reporting.<br>→ Not necessarily a sign of a biased study / not necessarily related to the validity of the study - but missing information can lead to wrong conclusions in this review --> not reliable		
Were the study subjects and the setting described in detail?	4. Certain diseases or conditions vary in prevalence across different geographic regions and populations (e.g. Women vs. Men, sociodemographic variables between countries). The study sample should be described in sufficient detail so that other researchers can determine if it is comparable to the population of interest to them.<br>*(important information: age, gender, length of hospital stay, information on medication / underlying diseases, information on in- or exclusion of repeated admissions)*	• just (mainly) yes = low risk<br>• ≤ 1x (mainly) no = some concerns<br>• ≥2x (mainly) no = high risk
Is the information on the proportion planned vs. unplanned admissions available?		
Is information available on the proportion of patients with medication vs. patients without medication in the study population?		
Availability of definitions: Was there a definition of what constitutes a		

"Medication related problem"?		
Availability of definitions: Were (categories of) preventability defined?	*(if applicable)*	
**G3 Issues in precision**<br>→ characteristic: Indication of random error / precicion (not „bias", an inadequate sample size might have an error by chance, so there will be an issue of imprecision – but it does not necessary have to be a wrong estimate, „for this small sample it is true")<br>→ This aspect will probably show up in wide confidence intervals of the study results		
Was the sample size adequate?	3. The larger the sample, the narrower will be the confidence interval around the prevalence estimate, making the results more precise. An adequate sample size is important to ensure good precision of the final estimate. Ideally we are looking for evidence that the authors conducted a sample size calculation to determine an adequate sample size. This will estimate how many subjects are needed to produce a reliable estimate of the measure(s) of interest. For conditions with a low prevalence, a larger sample size is needed. Also consider sample sizes for subgroup (or characteristics) analyses, and whether these are appropriate. Sometimes, the study will be large enough (as in large national surveys) whereby a sample size calculation is not required. In these cases, sample size can be considered adequate. When there is no sample size calculation and it is not a large national survey, the reviewers may consider conducting their own sample size analysis using the following formula: (Naing et al. 2006, Daniel 1999)<br>n= Z2P(1-P)/d^{2}; Where: n= sample size; Z = Z statistic for a level of confidence; P = Expected prevalence or proportion (in proportion of one; if 20%, P = 0.2); d = precision (in proportion of one; if 5%, d=0.05)<br>*(→ for an assumed prevalence of 2% (DRH of hospitalised patients), a required precision of 1% -> d=0.01: around 770 patients are needed)*<br>*(→ for an assumed prevalence of 0.2% (DRH of emergency patients), a required precision of 0.2% -> d=0.02: around 2000 patients are needed)*	• enough participants = low risk<br>• slightly too few participants = some concerns<br>• too few participants = high risk
**R. Risk of bias criteria**		
**R1 Selection bias**		
**R.1 Bias in selection of participants into the study**		
Was the sample frame appropriate to address the target population?	1. This question relies upon knowledge of the broader characteristics of the population of interest and the geographical area. If the study is of women with breast cancer, knowledge of at least the characteristics, demographics and medical history is needed. The term "target population" should not be taken to infer every individual from everywhere or with similar disease or exposure characteristics. Instead, give consideration to specific population characteristics in the study, including age range, gender, morbidities, medications, and other potentially influential factors. For example, a sample frame may not be appropriate to address the target population if a certain group has been used (such as those working for one organisation, or one profession) and the results then inferred to the target population (i.e. working adults). A sample frame may be appropriate when it includes almost all the members of the target population (i.e. a census, or a complete list of participants or complete registry data).<br>*(no selection bias when there are inclusion criteria for age categories or clinical conditions as these define the study population <--> for the following criteria, there is selection bias expected (as the results may not be valid for the study population:*<br>*1) study period < 10 month as seasonal effects may be present*<br>*2) if repeated admissions are excluded*<br>*3) if there is a minimum duration of stay required)*	• 1x yes + 1x (probably) yes = low risk<br>• ≥2x probably yes = some concerns<br>• 1x (probably) yes + 1x probably no = some concerns<br>• >1x (probably) no = high risk<br>• 1x (probably) yes + 1x no = high risk
Were study participants sampled in an appropriate way?	2. Studies may report random sampling from a population, and the methods section should report how sampling was performed. Random probabilistic sampling from a defined subset of the population (sample frame) should be employed in most cases, however, random probabilistic sampling is not needed when everyone in the sampling frame will be included/ analysed. For example, reporting on all the data from a good census is appropriate as a good census will identify everybody. When using cluster sampling, such as a random sample of villages within a region, the methods need to be clearly stated as the precision of the final prevalence estimate incorporates the clustering effect. Convenience samples, such as a street survey or interviewing lots of people at a public gatherings are not considered to provide a representative sample of the base population.<br>*(if "all" patients were evaluated → low risk of bias*<br>*If there is not entirely clearness about study population → selective exclusions? → risk of bias)*	
**R2, 3 Information bias**		
**R2 Information bias due to missing data (attrition and exclusions from the analysis)**		
Attrition bias:<br>Describe the completeness of outcome data for each main outcome (class), including attrition and exclusions from the analysis: Was the data analysis conducted with sufficient coverage of the identified sample? (→ response rate, dropouts)<br>Was the number of participants clear?	5. Coverage bias can occur when not all subgroups of the identified sample respond at the same rate. For instance, you may have a very high response rate overall for your study, but the response rate for a certain subgroup (i.e. older adults) may be quite low.<br>9. Was the response rate adequate, and if not, was the low response rate managed appropriately?<br>A large number of dropouts, refusals or "not founds" amongst selected subjects may diminish a study's validity, as can a low response rates for survey studies. The authors should clearly discuss the response rate and any reasons for non-response and compare persons in the study to those not in the study, particularly with regards to their socio-demographic characteristics. If reasons for non-response appear to be unrelated to the outcome measured and the characteristics of non-responders are comparable to those who do respond in the study (addressed in question 5, coverage bias), the researchers may be able to justify a more modest response rate.<br>*(if there are documented problems regarding unavailable records, missing patient files, exclusions due to "not documented" → some concerns of risk of bias)*	• yes = low risk<br>• 1x probably yes = some concerns<br>• ≥1x (probably) no = high risk

	**R3 Information bias regarding methods and measurements for classifications and outcomes**		
	Appraisal of the methods: Were valid methods used for the identification of the condition ("hospitalisations due to DRP" / "nature of DRH" --> e.g. preventability)? Were standard methods (validated tool) used in the assessment of causality and were they valid (for "hospitalisations due to DRP" / "nature of DRH" --> e.g. preventability)?	„CONDITION“ → ("hospitalisations due to DRP" / "nature of DRH" --> e.g. preventability)<br>6. Here we are looking for measurement or classification bias. Many health problems are not easily diagnosed or defined and some measures may not be capable of including or excluding appropriate levels or stages of the health problem. If the outcomes were assessed based on existing definitions or diagnostic criteria, then the answer to this question is likely to be yes. If the outcomes were assessed using observer reported, or self-reported scales, the risk of over- or under-reporting is increased, and objectivity is compromised. Importantly, determine if the measurement tools used were validated instruments as this has a significant impact on outcome assessment validity.	• 1x yes + 1x (probably) yes = low risk<br>• ≥2x probably yes = some concerns<br>• 1x (probably) yes + 1x probably no = some concerns<br>• >1x (probably) no = high risk<br>• 1x (probably) yes + 1x no = high risk
	Appraisal of the measurement: Was the condition measured in a standard, reliable way for all participants? Were the individuals (clinicians, self-reported, researchers) who identified determinants and outcomes?	7. Considerable judgment is required to determine the presence of some health outcomes. Having established the validity of the outcome measurement instrument (see item 6 of this scale), it is important to establish how the measurement was conducted. Were those involved in collecting data trained or educated in the use of the instrument/s? If there was more than one data collector, were they similar in terms of level of education, clinical or research experience, or level of responsibility in the piece of research being appraised? When there was more than one observer or collector, was there comparison of results from across the observers? Was the condition measured in the same way for all participants?	
	**R4 Information bias resulting from data analysis**		
	Frequency outcome: Was the denominator clearly defined?		• 1x yes + 1x (probably) yes = low risk<br>• ≥2x probably yes = some concerns<br>• 1x (probably) yes + 1x probably no = some concerns<br>• >1x (probably) no = high risk<br>• 1x (probably) yes + 1x no = high risk<br>If just one question could be answered:<br>• yes = low risk<br>• probably yes= some concerns<br>• ≥1 x (probably) no = high risk
	Was there appropriate statistical analysis?	8. Importantly, the numerator and denominator should be clearly reported, and percentages should be given with confidence intervals. The methods section should be detailed enough for reviewers to identify the analytical technique used and how specific variables were measured. Additionally, it is also important to assess the appropriateness of the analytical strategy in terms of the assumptions associated with the approach as differing methods of analysis are based on differing assumptions about the data and how it will respond.	
**R5 For analytical investigations on influencing factors:**<br>• **Issues regarding subgroups and adjusted analyses → covariates (for descriptive evaluations) or**<br>• **Issues regarding control for confounding factors (where causal relationships are derived)**			
	Are all important confounding factors / covariates / subgroups identified and accounted for?	JBL critical appraisal checklist for analytical cross-sectional studies:<br>5. Were confounding factors identified?<br>Confounding has occurred where the estimated intervention exposure effect is biased by the presence of some difference between the comparison groups (apart from the exposure investigated/of interest). Typical confounders include baseline characteristics, prognostic factors, or concomitant exposures (e.g. smoking). A confounder is a difference between the comparison groups and it influences the direction of the study results. A high-quality study at the level of cohort design will identify the potential confounders and measure them (where possible). This is difficult for studies where behavioral, attitudinal or lifestyle factors may impact on the results.<br>6. Were strategies to deal with confounding factors stated?<br>Strategies to deal with effects of confounding factors may be dealt within the study design or in data analysis. By matching or stratifying sampling of participants, effects of confounding factors can be adjusted for. When dealing with adjustment in data analysis, assess the statistics used in the study. Most will be some form of multivariate regression analysis to account for the confounding factors measured. *(Relevant factors: 1) Age; 2) gender; 3) number of comedication; 4) clinical condition of the patients: 4.1) cancer patients, 4.2) other patient groups with complex chronical diseases, or at least one measure of a patient characteristic related to the severity of the underlying disease (e.g. lenght of hospital stay, number of presence of comorbidities, organ (renal) insufficiency, severity of pre-existing disease), 5) other optional factors (if they were missing, this was not considered an increased risk of bias: e.g. off-label/unlicensed use, adiposity, socio-economic status, physician prescribing practices, ...))*	narrative reporting
	Were subpopulations, covariates and confounding factors identified using objective criteria?		
