# Supplementary material for: The epidemiology of drug-related hospital admissions in paediatrics – a systematic review
Source: Arch Public Health. 2024 Jun 4;82:81. doi: 10.1186/s13690-024-01295-4 (PMC11149243; doi:10.1186/s13690-024-01295-4)
Supplement: Supplementary file 4 — Additional file 4. Documentation: Results of full text screening. [file 13690_2024_1295_MOESM4_ESM.pdf]

Documentation of the records included after title and abstract screening, n = 269 ($n_{\text{identified “via databases”}}$ = 242 + $n_{\text{identified “via other methods”}}$ = 27)

Included records (n = 50 records)

Inclusions on full-text screening “via databases”	Inclusions based on abstracts for records with full-text not available (conference abstract or no access) “via databases”	Inclusions on full-text screening “via other methods”	Inclusions based on abstracts for records with full-text not available (conference abstract or no access) “via other methods”
n = 31<br>(1-31)	n = 10<br>(32-39) (conference abstracts);<br>(40, 41) (no access)	n = 7<br>(42-48)	n = 2<br>(49) (conference abstract);<br>(50) (no access)

Excluded records with reasons (n = 219)

The decision was based on the following criteria (as soon as one criterion was fulfilled, the decision to exclude was made (irrespective of whether further criteria would apply subsequently)

Reasons for exclusion	Exclusions on full-text screening “via databases”	Exclusions based on abstracts for records with full-text not available (conference abstract or no access) “via databases”	Exclusions on full-text screening “via other methods”	Exclusions based on abstracts for records with full-text not available (conference abstract or no access) “via other methods”
No (separate) paediatric data	n = 85<br>(51-135)	n = 15<br>(136-150)	n = 9<br>(151-159)	n = 0
No (separate) data on drug related hospital admissions compared to an appropriate reference population	n = 63<br>(160-222)	n = 15<br>(223-237)	n = 5<br>(238-242)	n = 1<br>(243)
No systematic quantitative study	n = 7<br>(244-250)	n = 0	n = 2<br>(251, 252)	n = 0
No generalisable data	n = 11<br>(253-263)	n = 5<br>(264-268)	n = 0	n = 0
			Additional excluded record: n = 1 (One record (269) could not be found at all and had to be excluded after title screening.)	

Bibliography

1. Bellis JR, Kirkham JJ, Nunn AJ, Pirmohamed M. Adverse drug reactions and off-label and unlicensed medicines in children: a prospective cohort study of unplanned admissions to a paediatric hospital. Br J Clin Pharmacol. 2014;77(3):545-53.
2. Bénard-Laribière A, Miremont-Salamé G, Pérault-Pochat MC, Noize P, Haramburu F. Incidence of hospital admissions due to adverse drug reactions in France: the EMIR study. Fundam Clin Pharmacol. 2015;29(1):106-11.
3. Buajordet I, Wesenberg F, Brørs O, Langslet A. Adverse drug events in children during hospitalization and after discharge in a Norwegian university hospital. Acta Paediatr. 2002;91(1):88-94.
4. Easton KL, Chapman CB, Brien JA. Frequency and characteristics of hospital admissions associated with drug-related problems in paediatrics. Br J Clin Pharmacol. 2004;57(5):611-5.
5. Easton-Carter KL, Chapman CB, Brien JE. Emergency department attendances associated with drug-related problems in paediatrics. J Paediatr Child Health. 2003;39(2):124-9.
6. Fattahi F, Pourpak Z, Moin M, Kazemnejad A, Khotaei GT, Mamishi S, et al. Adverse drug reactions in hospitalized children in a department of infectious diseases. J Clin Pharmacol. 2005;45(11):1313-8.
7. Feinstein JA, Feudtner C, Kempe A. Adverse drug event-related emergency department visits associated with complex chronic conditions. Pediatrics. 2014;133(6):e1575-85.
8. Gallagher RM, Bird KA, Mason JR, Peak M, Williamson PR, Nunn AJ, et al. Adverse drug reactions causing admission to a paediatric hospital: a pilot study. J Clin Pharm Ther. 2011;36(2):194-9.
9. Gallagher RM, Mason JR, Bird KA, Kirkham JJ, Peak M, Williamson PR, et al. Adverse drug reactions causing admission to a paediatric hospital. PLoS One. 2012;7(12):e50127.
10. Gholami K, Babaie F, Shalviri G, Javadi MR, Faghihi T. Pediatric hospital admission due to adverse drug reactions: Report from a tertiary center. J Res Pharm Pract. 2015;4(4):212-5.
11. Gupta S, Zaki SA, Masavkar S, Shanbag P. Causality, Severity, and Avoidability of Adverse Drug Reactions in Hospitalized Children: A Prospective Cohort Study. Cureus. 2023;15(1):e33369.
12. Haffner S, von Laue N, Wirth S, Thürmann PA. Detecting adverse drug reactions on paediatric wards: intensified surveillance versus computerised screening of laboratory values. Drug Saf. 2005;28(5):453-64.
13. Jonville-Béra AP, Giraudeau B, Blanc P, Beau-Salinas F, Autret-Leca E. Frequency of adverse drug reactions in children: a prospective study. Br J Clin Pharmacol. 2002;53(2):207-10.
14. Kang M-G, Lee J-Y, Woo S-I, Kim K-S, Jung J-W, Lim TH, et al. Adverse drug events leading to emergency department visits: A multicenter observational study in Korea. PLoS One. 2022;17(9):e0272743.
15. Lamabadusuriya SP, Sathiadas G. Adverse drug reactions in children requiring hospital admission. Ceylon Med J. 2003;48(3):86-7.
16. Langerová P, Vrtal J, Urbánek K. Adverse drug reactions causing hospital admissions in childhood: a prospective, observational, single-centre study. Basic Clin Pharmacol Toxicol. 2014;115(6):560-4.
17. Le J, Nguyen T, Law AV, Hodding J. Adverse drug reactions among children over a 10-year period. Pediatrics. 2006;118(2):555-62.
18. Lombardi N, Crescioli G, Bettiol A, Marconi E, Vitiello A, Bonaiuti R, et al. Characterization of serious adverse drug reactions as cause of emergency department visit in children: a 5-years active pharmacovigilance study. BMC Pharmacol Toxicol. 2018;19(1):16.
19. Morales-Ríos O, Cicero-Oneto C, García-Ruiz C, Villanueva-García D, Hernández-Hernández M, Olivar-López V, et al. Descriptive study of adverse drug reactions in a tertiary care pediatric hospital in México from 2014 to 2017. PLoS One. 2020;15(3):e0230576.
20. Mouton JP, Fortuin-de Smidt MC, Jobanputra N, Mehta U, Stewart A, de Waal R, et al. Serious adverse drug reactions at two children's hospitals in South Africa. BMC Pediatr. 2020;20(1):3.
21. Nasso C, Mecchio A, Rottura M, Valenzise M, Menniti-Ippolito F, Cutroneo PM, et al. A 7-Years Active Pharmacovigilance Study of Adverse Drug Reactions Causing Children Admission to a Pediatric Emergency Department in Sicily. Front Pharmacol. 2020;11:1090.
22. Neubert A, Toni I, Konig J, Malonga Makosi D, Mildenberger P, Romanos M, et al. A Complex Intervention to Prevent Medication-Related Hospital Admissions. Deutsches Arzteblatt international. 2023;120(25):425-31.
23. Oshikoya KA, Chukwura H, Njokanma OF, Senbanjo IO, Ojo I. Incidence and cost estimate of treating pediatric adverse drug reactions in Lagos, Nigeria. Sao Paulo Med J. 2011;129(3):153-64.
24. Patel PP, Makrani MM, Gandhi AM, Desai MK, Desai CK. An intensive monitoring of adverse drug reactions in pediatric hospitalized patients of a tertiary care hospital. International Journal of Basic & Clinical Pharmacology. 2021;10(6):704.

25. Posthumus AA, Alingh CC, Zwaan CC, van Grootheest KK, Hanff LL, Witjes BB, et al. Adverse drug reaction-related admissions in paediatrics, a prospective single-centre study. BMJ Open. 2012;2(4).

26. Pouyanne P, Haramburu F, Imbs JL, Bégaud B. Admissions to hospital caused by adverse drug reactions: cross sectional incidence study. French Pharmacovigilance Centres. Bmj. 2000;320(7241):1036.

27. Silva YDOM, Guimarães Lima M. Incidência de internações por eventos adversos a medicamentos em Minas Gerais. Sci Med. 2017;27(1):24936.

28. Tundia NL, Heaton PC, Kelton CM. The national burden of E-code-identified adverse drug events among hospitalized children using a national discharge database. Pharmacoepidemiol Drug Saf. 2011;20(8):866-78.

29. van der Hooft CS, Dieleman JP, Siemes C, Aarnoudse AJ, Verhamme KM, Stricker BH, et al. Adverse drug reaction-related hospitalisations: a population-based cohort study. Pharmacoepidemiol Drug Saf. 2008;17(4):365-71.

30. Weiss J, Krebs S, Hoffmann C, Werner U, Neubert A, Brune K, et al. Survey of adverse drug reactions on a pediatric ward: a strategy for early and detailed detection. Pediatrics. 2002;110(2 Pt 1):254-7.

31. Zed PJ, Black KJ, Fitzpatrick EA, Ackroyd-Stolarz S, Murphy NG, Curran JA, et al. Medication-related emergency department visits in pediatrics: a prospective observational study. Pediatrics. 2015;135(3):435-43.

32. Langerova P, Vrtal J, Urbánek K. Adverse drug reactions as cause of hospital admissions in childhood. International Journal of Clinical Pharmacy. 2013;35(6):1265.

33. Matalová P, Vrtal J, Urbánek K. Adverse drug reactions in children. Clin Ther. 2017;39(8):e105.

34. Mendoza Otero F, Iniesta Navalon C, García Molina O, Fernandez De Palencia Espinosa M, Galindo Rueda M, De La Rubia Nieto A. Adverse drug reactions causing admission over 11 years in a paediatric hospital. Eur J Hosp Pharm. 2015;22(Suppl 1):A184.3-A5.

35. Moschini M, Lombardi N, Pugi A, Maggini V, Gallo E, Lenti MC, et al. Monitoring program of adverse drug reactions in a pediatric emergency department. Drug Saf. 2013;36(9):913.

36. Mouton JP, Jobanputra N, Mehta U, Technau K, Scott C, Cohen K. Adverse drug reactions among Hospitalised Children in South Africa. Pharmacoepidemiology and Drug Safety. 2016;25(427).

37. Oshikoya KA, Senbanjo IO, Njokanma OF, Soipe AI. Economic burden of managing adverse drug reactions in children in Lagos, Nigeria. Pharmacoepidemiol Drug Saf. 2009;18(S1):S228-S9.

38. Romano-Lieber NS, Ribeiro E. Adverse drug events leading children to emergency department, São Paulo, Brazil. Pharmacoepidemiology and Drug Safety. 2011;20((Romano-Lieber N.S.) School of Public Health, University of São Paulo, São Paulo, SP, Brazil):S320-S1.

39. Toni I, Wimmer S, Trollmann R, Rascher W, Neubert A. Drug-related hospital admissions in paediatrics-what is preventable? Archives of Disease in Childhood. 2019;104(6).

40. Impicciatore P, Mohn A, Chiarelli F, Pandolfini C, Bonati M. Adverse drug reactions to off-label drugs on a paediatric ward: An Italian prospective pilot study. Paediatric and Perinatal Drug Therapy. 2002;5(1):19-24.

41. Oshikoya KA, Njokanma OF, Chukwura HA, Ojo IO. Adverse drug reactions in Nigerian children. Paediatric and Perinatal Drug Therapy. 2007;8(2):81-8.

42. Bourgeois FT, Mandl KD, Valim C, Shannon MW. Pediatric adverse drug events in the outpatient setting: an 11-year national analysis. Pediatrics. 2009;124(4):e744-50.

43. Cohen AL, Budnitz DS, Weidenbach KN, Jernigan DB, Schroeder TJ, Shehab N, et al. National surveillance of emergency department visits for outpatient adverse drug events in children and adolescents. J Pediatr. 2008;152(3):416-21.

44. McDonnell PJ, Jacobs MR. Hospital admissions resulting from preventable adverse drug reactions. Ann Pharmacother. 2002;36(9):1331-6.

45. Rosafio C, Paioli S, Del Giovane C, Cenciarelli V, Viani N, Bertolani P, et al. Medication-related visits in a pediatric emergency department: an 8-years retrospective analysis. Ital J Pediatr. 2017;43(1):55.

46. Russom M, Gebrehiwet S, Afendi A, Mekonnen E, Tedla K, Tesfai D. Failed Lactation Associated with Medroxyprogesterone Acetate: Cases Series Assessment. IJP. 2017;2(1):1-5.

47. Sikdar KC, Alaghehbandan R, Macdonald D, Barrett B, Collins KD, Gadag V. Adverse drug events among children presenting to a hospital emergency department in Newfoundland and Labrador, Canada. Pharmacoepidemiol Drug Saf. 2010;19(2):132-40.

48. Temple ME, Robinson RF, Miller JC, Hayes JR, Nahata MC. Frequency and preventability of adverse drug reactions in paediatric patients. Drug Saf. 2004;27(11):819-29.

49. Speranza N, Lucas L, Telechea H, Santurio A, Giachetto G, Nanni L. Adverse Drugs Reactions in Hospitalized Children: A Public Health Problem. Drug Saf. 2008;31(10):885.

50. Duczmal E, Bręborowicz A. Adverse drug reactions as a cause of hospital admission. Prz Pediatr. 2006;36(1):14-8.

51. Ahmad A, Parimalakrishnan S, Mohanta GP, Manna PK, Manavalan R. Incidence of adverse drug reactions with commonly prescribed drugs in tertiary care teaching hospital in India. International Journal of Research in Pharmaceutical Sciences. 2012;3(1):79-83.
52. Al-Olah YH, Al Thiab KM. Admissions through the emergency department due to drug-related problems. Annals of Saudi medicine. 2008;28(6):426-9.
53. Alayed N, Alkhalifah B, Alharbi M, Alwohaibi N, Farooqui M. Adverse drug reaction (ADR) as a cause of hospitalization at a government hospital in saudi arabia: A prospective observational study. Current Drug Safety. 2019;14(3):192-8.
54. Alexopoulou A, Dourakis SP, Mantzoukis D, Pitsariotis T, Kandyli A, Deutsch M, et al. Adverse drug reactions as a cause of hospital admissions: a 6-month experience in a single center in Greece. Eur J Intern Med. 2008;19(7):505-10.
55. Banholzer S, Dunkelmann L, Haschke M, Derungs A, Exadaktylos A, Krähenbühl S, et al. Retrospective analysis of adverse drug reactions leading to short-term emergency hospital readmission. Swiss Med Wkly. 2021;151:w20400.
56. Bartu A, Freeman NC, Gawthorne GS, Codde JP, Holman CD. Mortality in a cohort of opiate and amphetamine users in Perth, Western Australia. Addiction. 2004;99(1):53-60.
57. Barvaliya M, Sanmukhani J, Patel T, Paliwal N, Shah H, Tripathi C. Drug-induced Stevens-Johnson syndrome (SJS), toxic epidermal necrolysis (TEN), and SJS-TEN overlap: a multicentric retrospective study. J Postgrad Med. 2011;57(2):115-9.
58. Beena JS, Rakesh Praveen Raj MR, Reeja R, Bindulatha Nair R, Pavithran RL. Adverse drug reactions among patients in department of medicine in a tertiary care teaching hospital, Kollam. Asian Journal of Pharmaceutical and Clinical Research. 2023;16(12):65-8.
59. Borch JE, Andersen KE, Bindslev-Jensen C. Cutaneous adverse drug reactions seen at a university hospital department of dermatology. Acta Derm Venereol. 2006;86(6):523-7.
60. Bourgeois FT, Shannon MW, Valim C, Mandl KD. Adverse drug events in the outpatient setting: an 11-year national analysis. Pharmacoepidemiol Drug Saf. 2010;19(9):901-10.
61. Brito-Zerón P, Soria N, Muñoz S, Bové A, Akasbi M, Belenguer R, et al. Prevalence and clinical relevance of autoimmune neutropenia in patients with primary Sjögren's syndrome. Semin Arthritis Rheum. 2009;38(5):389-95.
62. Buajordet I, Naess AC, Jacobsen D, Brørs O. Adverse events after naloxone treatment of episodes of suspected acute opioid overdose. Eur J Emerg Med. 2004;11(1):19-23.
63. Budnitz DS, Pollock DA, Mendelsohn AB, Weidenbach KN, McDonald AK, Annest JL. Emergency department visits for outpatient adverse drug events: demonstration for a national surveillance system. Ann Emerg Med. 2005;45(2):197-206.
64. Budnitz DS, Pollock DA, Weidenbach KN, Mendelsohn AB, Schroeder TJ, Annest JL. National surveillance of emergency department visits for outpatient adverse drug events. Jama. 2006;296(15):1858-66.
65. Calvo-Salazar RA, David M, Zapata-Mesa MI, Rodríguez-Naranjo CM, Valencia-Acosta NY. Problemas relacionados con medicamentos que causan ingresos por urgencias en un hospital de alta complejidad. Farm Hosp. 2018;42(6):228-33.
66. Camargo AL, Cardoso Ferreira MB, Heineck I. Adverse drug reactions: a cohort study in internal medicine units at a university hospital. Eur J Clin Pharmacol. 2006;62(2):143-9.
67. Capuano A, Motola G, Russo F, Avolio A, Filippelli A, Rossi F, et al. Adverse drug events in two emergency departments in Naples, Italy: an observational study. Pharmacol Res. 2004;50(6):631-6.
68. Carrasco-Garrido P, de Andrés LA, Barrera VH, de Miguel GA, Jiménez-García R. Trends of adverse drug reactions related-hospitalizations in Spain (2001-2006). BMC Health Serv Res. 2010;10:287.
69. Cazacu I, Stroe R, Dondera R, Mogosan C, Haramburu F, Fourrier-Réglat A, et al. Adverse drug reactions of analgesic medicines: analysis of the Romanian pharmacovigilance database. Fundam Clin Pharmacol. 2018;32(3):330-6.
70. Chaio S, Toibaro J, Valicenti P, Saidon P. [Adverse drug reactions and prescription errors: morbi-mortality]. Reacciones adversas medicamentosas y errores de prescripcion: morbi-mortalidad. 2013;73(2):111-8.
71. Chandra S, Jaybhaye DL, Kaur S, Ubale A. Pharmacovigilance: Study of pattern of adverse drug reactions at a tertiary care teaching hospital, Aurangabad, and Maharashtra, India: A prospective study and forthcoming challenge for the future. National Journal of Physiology, Pharmacy and Pharmacology. 2023;13(9):1872-8.
72. Chatterjee S, Verma VK, Hazra A, Pal J. An observational study on acute poisoning in a tertiary care hospital in West Bengal, India. Perspect Clin Res. 2020;11(2):75-80.
73. Chung SJ, Ahn KM, Oh JH, Shim JS, Park HW. Incidence rates of severe cutaneous adverse reactions due to antiseizure medication: A nationwide study using health claims data in Korea. Epilepsia. 2021;62(1):250-7.
74. Collao Juan F, Favereau R, Miranda R, Aceiton C. [Drug related harm in Chilean hospitals: prevalence analysis 2010-2017]. Dano asociado al uso de medicamentos en hospitales chilenos: analisis de prevalencia 2010-2017. 2019;147(4):416-25.
75. de Almeida SM, Romualdo A, de Abreu Ferraresi A, Zelezoglo GR, Marra AR, Edmond MB. Use of a trigger tool to detect adverse drug reactions in an emergency department. BMC Pharmacol Toxicol. 2017;18(1):71.
76. Esteban Jiménez Ó, Navarro Pemán C, González Rubio F, Lanuza Giménez FJ, Montesa Lou C. [A study of incidence and clinical characteristics of adverse drug reactions in hospitalized patients.]. Rev Esp Salud Publica. 2017;91.

77. Farzaneh E, Amani F, Etemad F. A clinico-epidemiologic study on patients with opium toxicity treated at Ardabil Hospitals, Iran, 2014-2015. Asia Pacific Journal of Medical Toxicology. 2016;5(4):111-4.
78. Fernandes K, Martins D, Juurlink D, Mamdani M, Paterson JM, Spooner L, et al. High-Dose Opioid Prescribing and Opioid-Related Hospitalization: A Population-Based Study. PLoS One. 2016;11(12):e0167479.
79. Franceschi A, Tuccori M, Bocci G, Vannozzi F, Di Paolo A, Barbara C, et al. Drug therapeutic failures in emergency department patients. A university hospital experience. Pharmacol Res. 2004;49(1):85-91.
80. Gillooly I, Tan EC, Wojt IR, Patanwala AE, Cairns R. Changes in medication regimen complexity index following medication-related hospital admissions: A retrospective single-centre study. Research in social & administrative pharmacy : RSAP. 2023;19(6):969-72.
81. Green CF, Mottram DR, Rowe PH, Pirmohamed M. Adverse drug reactions as a cause of admission to an acute medical assessment unit: a pilot study. J Clin Pharm Ther. 2000;25(5):355-61.
82. Grira M, Larbi T, El Ouni A, Bouslama K, Abdallah M, Harmel A, et al. The incidence of serious adverse events in a tunisian hospital: a retrospective medical record review study. Tunis Med. 2015;93(12):795-9.
83. Hafner JW, Jr., Belknap SM, Squillante MD, Bucheit KA. Adverse drug events in emergency department patients. Ann Emerg Med. 2002;39(3):258-67.
84. Isik MN, Dalgic N, Okuyan B, Yildirmak ZY, Sancar M. Medication Review for Hospitalized Pediatric Patient: Clinical Pharmacist Interventions. J Pediatr Infect. 2020;14(4):237-43.
85. Jiang A, Smith J, Rajabali F, Zheng A, Purssell R, Pike I. Patterns in poisoning hospitalizations and deaths in British Columbia, 2008 to 2013. BRITISH COLUMBIA MEDICAL JOURNAL. 2018;60(10):495-502.
86. Jose J, Rao PG. Pattern of adverse drug reactions notified by spontaneous reporting in an Indian tertiary care teaching hospital. Pharmacol Res. 2006;54(3):226-33.
87. Juntti-Patinen L, Kuitunen T, Pere P, Neuvonen PJ. Drug-related visits to a district hospital emergency room. Basic Clin Pharmacol Toxicol. 2006;98(2):212-7.
88. Kareem SA, Sridhar SB, Shetty MS. Intensive monitoring of adverse drug reactions in nephrology unit of tertiary care teaching hospital. Saudi journal of kidney diseases and transplantation : an official publication of the Saudi Center for Organ Transplantation, Saudi Arabia. 2019;30(5):1075-83.
89. Karuppannan M, Nee TK, Ali SM, Thong WK, Boardman H. The prevalence of adverse drug event-related admissions at a local hospital in Malaysia. Archives of Pharmacy Practice. 2013;4(4):160-7.
90. Koh Y, Fatimah BM, Li SC. Therapy related hospital admission in patients on polypharmacy in Singapore: a pilot study. Pharm World Sci. 2003;25(4):135-7.
91. Kongkaew C, Hann M, Mandal J, Williams SD, Metcalfe D, Noyce PR, et al. Risk factors for hospital admissions associated with adverse drug events. Pharmacotherapy. 2013;33(8):827-37.
92. Labourel H, Ladwig M, Maurin C, Saviuc P, Danel V, Loizzo F, et al. [Epidemiological analysis of drug self-poisonings treated by a mobile intensive care unit]. Analyse epidemiologique des intoxications medicamenteuses volontaires aigues: prise en charge par un service mobile d'urgence et de reanimation. 2006;61(3):185-9.
93. Lacoste-Roussillon C, Pouyanne P, Haramburu F, Miremont G, Bégaud B. Incidence of serious adverse drug reactions in general practice: a prospective study. Clin Pharmacol Ther. 2001;69(6):458-62.
94. Laubscher C, Van Rooyen EEM. The profile of the overdose patient presenting at Paarl Hospital Emergency Department. South African Family Practice. 2007;49(2):16.
95. Lavan AH, O'Mahony D, Buckley M, O'Mahony D, Gallagher P. Adverse Drug Reactions in an Oncological Population: Prevalence, Predictability, and Preventability. Oncologist. 2019;24(9):e968-e77.
96. Li R, Curtis K, Zaidi STR, Van C, Thomson A, Castelino R. Prevalence, characteristics, and reporting of adverse drug reactions in an Australian hospital: a retrospective review of hospital admissions due to adverse drug reactions. Expert Opin Drug Saf. 2021;20(10):1267-74.
97. McLachlan CY, Yi M, Ling A, Jardine DL. Adverse drug events are a major cause of acute medical admission. Intern Med J. 2014;44(7):633-8.
98. Miguel A, Bernardo M, Freitas A, Lopes F, Azevedo L, Pereira AC. Detection of adverse drug reactions using hospital databases-a nationwide study in Portugal. Pharmacoepidemiol Drug Saf. 2013;22(8):907-13.
99. Miliszewski MA, Kirchhof MG, Sikora S, Papp A, Dutz JP. Stevens-Johnson Syndrome and Toxic Epidermal Necrolysis: An Analysis of Triggers and Implications for Improving Prevention. Am J Med. 2016;129(11):1221-5.
100. Miller GC, Britth HC, Valenti L. Adverse drug events in general practice patients in Australia. Med J Aust. 2006;184(7):321-4.
101. Miranda V, Fede A, Nobuo M, Ayres V, Giglio A, Miranda M, et al. Adverse drug reactions and drug interactions as causes of hospital admission in oncology. J Pain Symptom Manage. 2011;42(3):342-53.
102. Noblat AC, Noblat LA, Toledo LA, Santos PM, Oliveira MG, Tanajura GM, et al. [Prevalence of hospital admission due to adverse drug reaction in Salvador, Bahia]. Revista da Associação Médica Brasileira (1992). 2011;57(1):42-5.

103. Pedrós C, Quintana B, Rebolledo M, Porta N, Vallano A, Arnau JM. Prevalence, risk factors and main features of adverse drug reactions leading to hospital admission. Eur J Clin Pharmacol. 2014;70(3):361-7.

104. Pfaffenbach G, Carvalho OM, Bergsten-Mendes G. [Drug adverse reactions leading to hospital admission]. Rev Assoc Med Bras (1992). 2002;48(3):237-41.

105. Pirmohamed M, James S, Meakin S, Green C, Scott AK, Walley TJ, et al. Adverse drug reactions as cause of admission to hospital: prospective analysis of 18 820 patients. Bmj. 2004;329(7456):15-9.

106. Popova S, Rehm J, Patra J, Baliunas D, Taylor B. Illegal drug-attributable morbidity in Canada 2002. Drug Alcohol Rev. 2007;26(3):251-63.

107. Poudel DR, Acharya P, Ghimire S, Dhital R, Bharati R. Burden of hospitalizations related to adverse drug events in the USA: a retrospective analysis from large inpatient database. Pharmacoepidemiol Drug Saf. 2017;26(6):635-41.

108. Pourseyed S, Fattahi F, Pourpak Z, Gholami K, Shariatpanahi SS, Moin A, et al. Adverse drug reactions in patients in an Iranian department of internal medicine. Pharmacoepidemiol Drug Saf. 2009;18(2):104-10.

109. Rajapakse T, Griffiths KM, Christensen H, Cotton S. A comparison of non-fatal self-poisoning among males and females, in Sri Lanka. BMC Psychiatry. 2014;14(1).

110. Rojas-Velandia C, Ruiz-Garzón J, Moscoso-Alcina JC, Vallejos-Narvaéz Á, Castro-Canoa J, Bustos-Martínez Y, et al. Characterization of adverse drug reactions causing admission to an intensive care unit. Br J Clin Pharmacol. 2017;83(5):1134-40.

111. Saheb Sharif-Askari F, Saheb Sharif-Askari N, Javadi M, Gholami K. Adverse drug reactions reported to the drug and poison information center of Tehran, Iran. PLoS One. 2017;12(9):e0185450.

112. Sánchez Muñoz-Torrero JF, Barquilla P, Velasco R, Fernández Capitan Mdel C, Pacheco N, Vicente L, et al. Adverse drug reactions in internal medicine units and associated risk factors. Eur J Clin Pharmacol. 2010;66(12):1257-64.

113. Santos GAS, Boing AC. [Hospitalizations and deaths from drug poisoning and adverse reactions in Brazil: an analysis from 2000 to 2014]. Cad Saude Publica. 2018;34(6):e00100917.

114. Sarah B, Lea D, Manuel H, Adrian D, Aristomenis E, Stephan K, et al. Retrospective analysis of adverse drug reactions leading to short-term emergency hospital readmission. Swiss Medical Weekly. 2021;151(3-4).

115. Sauer BC, Hepler CD, Cherney B, Williamson J. Computerized indicators of potential drug-related emergency department and hospital admissions. Am J Manag Care. 2007;13(1):29-35.

116. Savitha RS, Kuruvilla AV, Manikanta N, Ashritha AS, Kurian J, Wilson D. A study on medication-related hospital admissions in a tertiary care hospital. International Journal of Research in Pharmaceutical Sciences. 2020;11(Special Issue 4):1432-9.

117. Schmiedl S, Rottenkolber M, Hasford J, Rottenkolber D, Farker K, Drewelow B, et al. Self-medication with over-the-counter and prescribed drugs causing adverse-drug-reaction-related hospital admissions: results of a prospective, long-term multi-centre study. Drug Saf. 2014;37(4):225-35.

118. Schneeweiss S, Hasford J, Göttler M, Hoffmann A, Riethling AK, Avorn J. Admissions caused by adverse drug events to internal medicine and emergency departments in hospitals: a longitudinal population-based study. Eur J Clin Pharmacol. 2002;58(4):285-91.

119. Schurig AM, Böhme M, Just KS, Scholl C, Dormann H, Plank-Kiegele B, et al. Adverse Drug Reactions (ADR) and Emergencies. Dtsch Arztebl Int. 2018;115(15):251-8.

120. Schwarz UI, Rüder S, Krappweis J, Israel M, Kirch W. [Epidemiology of attempted suicide using drugs. An inquiry from the Dresden University Clinic]. Dtsch Med Wochenschr. 2004;129(31-32):1669-73.

121. Seetoh T, Lye DC, Cook AR, Archuleta S, Chan M, Sulaiman Z, et al. An outcomes analysis of outpatient parenteral antibiotic therapy (OPAT) in a large Asian cohort. Int J Antimicrob Agents. 2013;41(6):569-73.

122. Sheinicharmian A, Nagaraju K. Patterns of Poisoning and the Treatment Outcome in patients admitted to the Department of Emergency Medicine, KIMS Hospital, Bangalore, India. Pakistan Journal of Medical and Health Sciences. 2019;13(2):531-4.

123. Stausberg J, Hasford J. Drug-related admissions and hospital-acquired adverse drug events in Germany: a longitudinal analysis from 2003 to 2007 of ICD-10-coded routine data. BMC Health Serv Res. 2011;11:134.

124. Sudha TYS, Vangoori Y, Varghese AM. A profile of adverse drug reactions in a teritiary care teaching hospital and associated factors. Biomedical and Pharmacology Journal. 2021;14(1):367-71.

125. Thuermann PA, Windecker R, Steffen J, Schaefer M, Tenter U, Reese E, et al. Detection of adverse drug reactions in a neurological department: comparison between intensified surveillance and a computer-assisted approach. Drug Saf. 2002;25(10):713-24.

126. Tomlin A, Woods DJ, Lambie A, Eskildsen L, Ng J, Tilyard M. Ethnic inequality in non-steroidal anti-inflammatory drug-associated harm in New Zealand: A national population-based cohort study. Pharmacoepidemiol Drug Saf. 2020;29(8):881-9.

127. Trifirò G, Calogero G, Ippolito FM, Cosentino M, Giuliani R, Conforti A, et al. Adverse drug events in emergency department population: a prospective Italian study. Pharmacoepidemiol Drug Saf. 2005;14(5):333-40.

128. Vaghar MI. An investigation into the prevalence and causes of drug and chemical poisoning in patients. Asian Journal of Pharmaceutical and Clinical Research. 2018;11(7):467-70.
129. Vivolo-Kantor A, Pasalic E, Liu S, Martinez PD, Gladden RM. Defining indicators for drug overdose emergency department visits and hospitalisations in ICD-10-CM coded discharge data. Inj Prev. 2021;27(S1):i56-i61.
130. Vonbach P, Dubied A, Krähenbühl S, Beer JH. Prevalence of drug-drug interactions at hospital entry and during hospital stay of patients in internal medicine. Eur J Intern Med. 2008;19(6):413-20.
131. Vonbach P, Reich R, Möll F, Krähenbühl S, Ballmer PE, Meier CR. Risk factors for gastrointestinal bleeding: a hospital-based case-control study. Swiss Med Wkly. 2007;137(49-50):705-10.
132. Wojt IR, Cairns R, Gillooly I, Patanwala AE, Tan ECK. Clinical factors associated with increased length of stay and readmission in patients with medication-related hospital admissions: a retrospective study. Res Social Adm Pharm. 2022;18(7):3184-90.
133. Woods DM, Thomas EJ, Holl JL, Weiss KB, Brennan TA. Ambulatory care adverse events and preventable adverse events leading to a hospital admission. Qual Saf Health Care. 2007;16(2):127-31.
134. Zhang H, Du W, Gnjidic D, Chong S, Glasgow N. Trends in adverse drug reaction-related hospitalisations over 13 years in New South Wales, Australia. Intern Med J. 2019;49(1):84-93.
135. Zhong H, Zhou Z, Wang H, Niu J, Chen W, Song Z, et al. Prevalence of cutaneous adverse drug reactions in Southwest China: an 11-year retrospective survey on in-patients of a dermatology ward. Dermatitis. 2012;23(2):81-5.
136. Moro PA, Assisi F, Bissoli M, Borghini R, Davanzo F, Della Puppa T, et al. Do people use over-the-counter drugs safely? An analysis of 409 cases of domestic medication errors with acetaminophen reported from the Poison Control Centre of Milan, Italy. Clinical Toxicology. 2010;48(3):263.
137. Barnard M, Banahan BF. Epidemiology of adolescent and young adult hospital utilization for alcohol and drug use, suicide, and poisoning in the United States. Value in Health. 2011;14(3):A112.
138. Stankova E BE, Hubenova A. A survey of adverse drug reactions based on data from a toxicology unit. Clinical Toxicology. 2012;50(4):327.
139. Brown JA, Johnston CI. Benzyl benzoate burns bad: 339 poison center adverse reaction reports and counting. Clinical Toxicology. 2014;52:326.
140. Ceschi A, Conen D, Herzig L, Staehelin A, Zoller M, Gnädinger M. Medication incidents in primary care medicine: Prospective observational pilot study with case-control analysis. Clinical Toxicology. 2014;52:325.
141. Du P, Mathibe L. Hospitalization of patients due to traditional medicine poisoning. Basic and Clinical Pharmacology and Toxicology. 2014;115:357.
142. Ruggiero F, Taurasi F, Aliberti FA, Micera D, Melillo A, Russo L. Serious adverse drug events: The role of the hospital pharmacist in Campania Region's emergency department. Eur J Hosp Pharm. 2014;21:A204.
143. Stabile S, Ruggiero F, Taurasi F, Vigano M, Borin F. Incidence and risk factors of adverse drug reactions in the general population observed through an active pharmacovigilance project. Eur J Hosp Pharm. 2014;21:A203-A4.
144. Lelièvre B, Peucelle D, Auffret M, Bene J, Gautier S, Mathieu M. Appropriate use of medicines: Interest of a collaboration between poison control center and regional pharmacovigilance center. Fundamental and Clinical Pharmacology. 2015;29:59.
145. Aljadhey H, Mahmoud MA, Hassali MA. Prescribing errors incidence in Four Hospitals in Saudi Arabia. Pharmacoepidemiology and Drug Safety. 2016;25:422.
146. Cairns R, Brown JA, Buckley NA. Eucalyptus oil poisoning in Australia: Do we need koala-proof packaging? Clinical Toxicology. 2016;54(4):376.
147. Kurian J, Savitha RS, Kuruvilla A, Nalla M, Ashritha A, Wilson D, et al. Drug-related hospital admission: Predictors and cost implicated. Pharmacoepidemiology and Drug Safety. 2017;26:144-5.
148. Li D, Li R, Leong D, Dewitt C. Practice points for prescribing and dispensing colchicine identified from a retrospective review of colchicine calls to a poison control centre. Clinical Toxicology. 2017;55(7):738-9.
149. Madhan R, Aishwarya P, James A, John GG, Mihir M, Harsha CS. Medication related hospital admissions to a tertiary care teaching hospital: A prospective cross sectional study. Pharmacoepidemiology and Drug Safety. 2019;28:355-6.
150. Buelow B, Alluri S. The Burden Of Self-Reported Adverse Drug Reactions in Patients Referred To An Outpatient Allergy Practice At A Tertiary Referral Center. Journal of Allergy and Clinical Immunology. 2021;147(2):AB14.
151. Bordet R, Gautier S, Le Louet H, Dupuis B, Caron J. Analysis of the direct cost of adverse drug reactions in hospitalised patients. Eur J Clin Pharmacol. 2001;56(12):935-41.
152. Mjörndal T, Boman MD, Hägg S, Bäckström M, Wiholm BE, Wahlin A, et al. Adverse drug reactions as a cause for admissions to a department of internal medicine. Pharmacoepidemiol Drug Saf. 2002;11(1):65-72.
153. Bannwarth B, Queneau P, Carpentier F, Guliana JM, Bouget J, Trombert B. Hospital visits caused by adverse drug reactions: incidence and preventability assessed in French primary care/emergency departments. Drug Saf. 2003;26(2):133-4.

154. Peyriere H, Cassan S, Floutard E, Riviere S, Blayac JP, Hillaire-Buys D, et al. Adverse drug events associated with hospital admission. Ann Pharmacother. 2003;37(1):5-11.

155. Ramesh M, Pandit J, Parthasarathi G. Adverse drug reactions in a south Indian hospital--their severity and cost involved. Pharmacoepidemiol Drug Saf. 2003;12(8):687-92.

156. Hopf Y, Watson M, Williams D. Adverse-drug-reaction related admissions to a hospital in Scotland. Pharm World Sci. 2008;30(6):854-62.

157. Leendertse AJ, Egberts AC, Stoker LJ, van den Bemt PM. Frequency of and risk factors for preventable medication-related hospital admissions in the Netherlands. Arch Intern Med. 2008;168(17):1890-6.

158. Zed PJ, Abu-Laban RB, Balen RM, Loewen PS, Hohl CM, Brubacher JR, et al. Incidence, severity and preventability of medication-related visits to the emergency department: a prospective study. Cmaj. 2008;178(12):1563-9.

159. Capuano A, Irpino A, Gallo M, Ferrante L, Illiano ML, Rinaldi B, et al. Regional surveillance of emergency-department visits for outpatient adverse drug events. Eur J Clin Pharmacol. 2009;65(7):721-8.

160. Akici N, Bayoğlu D, Gürbüz T, Önal E, Nuhoğlu Ç, Akici A. Evaluation of toxicities seen in young and older children. Marmara Pharmaceutical Journal. 2013;17(1):35-41.

161. Amsalu A, Baraki AG, Muche EA. Drug-Related Problems and associated factors among hospitalized pediatric patients at the University of Gondar Comprehensive and Specialized Hospital. 2022.

162. Anthony L, Pundarikaksha HP. Patterns, causality, severity, and preventability of adverse drug reactions: An observational study at a tertiary health-care center. National Journal of Physiology, Pharmacy and Pharmacology. 2022;12(12):2155-8.

163. Baby M, Rg A, Mathew AE, Shajahan J. AN ANALYSIS OF THE PATTERN AND THE RISK FACTORS OF ADVERSE DRUG REACTIONS AT A TERTIARY CARE HOSPITAL. Asian Journal of Pharmaceutical and Clinical Research. 2022;15(9):41-6.

164. Bartlett AW, Mohamed TJ, Sudjaritruk T, Kurniati N, Nallusamy R, Hansudewechakul R, et al. Disease- and Treatment-related Morbidity in Adolescents With Perinatal HIV Infection in Asia. Pediatr Infect Dis J. 2019;38(3):287-92.

165. Baxter R, Toback SL, Sifakis F, Hansen J, Bartlett J, Aukes L, et al. A postmarketing evaluation of the safety of Ann Arbor strain live attenuated influenza vaccine in children 5 through 17 years of age. Vaccine. 2012;30(19):2989-98.

166. Beeler PE, Stammschulte T, Dressel H. Hospitalisations Related to Adverse Drug Reactions in Switzerland in 2012-2019: Characteristics, In-Hospital Mortality, and Spontaneous Reporting Rate. Drug Saf. 2023;46(8):753-63.

167. Bekele F, Bereda G, Tamirat L, Geleta BA, Jabessa D. "Childrens are not just "little adults". The rate of medication related problems and its predictors among patients admitted to pediatric ward of southwestern Ethiopian hospital: A prospective observational study. Ann Med Surg (Lond). 2021;70:102827.

168. Bentur Y, Obchinikov ND, Cahana A, Kovler N, Bloom-Krasik A, Lavon O, et al. Pediatric poisonings in Israel: National Poison Center data. Isr Med Assoc J. 2010;12(9):554-9.

169. Bizuneh GK, Adamu BA, Bizuayehu GT, Adane SD. A Prospective Observational Study of Drug Therapy Problems in Pediatric Ward of a Referral Hospital, Northeastern Ethiopia. International Journal of Pediatrics (United Kingdom). 2020;2020.

170. Caetano SCRC, da Silva LFE, Guaraldo L, Giordani F. Identifying adverse drug events in patients at a pediatric ward in a Brazilian hospital: application and performance of the triggers. Revista Brasileira de Saude Materno Infantil. 2021;21(4):1075-82.

171. Can ZY, Bayraktar S, Sancar M, Büyükkayhan D, Apikoğlu S. Identifying drug-related problems in the pediatric intensive care unit and evaluating clinical pharmacist interventions. International Journal of Clinical Pharmacy. 2022;44(6):1475-6.

172. Carvalho IV, de Sousa VM, Visacri MB, Quintanilha JCF, de Souza CM, Ambrósio RFL, et al. Adverse Drug Event-Related Admissions to a Pediatric Emergency Unit. Pediatr Emerg Care. 2021;37(4):e152-e8.

173. Cheraghali F, Taymori M. Epidemiological study of drug intoxication in children. Acta Medica Iranica. 2006;44(1):37-40.

174. Dittrich ATM, Draaisma JMT, van Puijenbroek EP, Loo D. Analysis of Reporting Adverse Drug Reactions in Paediatric Patients in a University Hospital in the Netherlands. Paediatr Drugs. 2020;22(4):425-32.

175. Dos S, Martinbiancho JK, Silva MM, Da Silva RG. Adverse drug reactions in general pediatrics units of a University Hospital. Latin American Journal of Pharmacy. 2009;28(5):695-9.

176. Fekadu G, Abdisa E, Fanta K. Medication prescribing errors among hospitalized pediatric patients at Nekemte Referral Hospital, western Ethiopia: cross-sectional study. BMC Res Notes. 2019;12(1):421.

177. Fujiwara T, Teshima R, Sugiyama I, Tsuchida K, Ohtsuka Y. Oxcarbazepine adjunctive therapy for partial seizures in japanese pediatric patients: A randomized double-blind placebo-controlled study and open-label extension study. Epilepsy and Seizure. 2019;11(1):30-45.

178. Gheshlaghi F, Piri-Ardakani MR, Yaraghi M, Shafiei F, Behjati M. Acute poisoning in children; a population study in isfahan, iran, 2008-2010. Iran J Pediatr. 2013;23(2):189-93.

179. Gholami N, Alwasabi F, Farnaghi F. Drug-Induced Apnea in Children Admitted to Loghman Hakim Hospital, Tehran, Iran. Iran J Child Neurol. 2017;11(3):15-8.

180. Gnanamanickam ES, Nguyen H, Armfield JM, Doidge JC, Brown DS, Preen DB, et al. Hospitalizations among children involved in the child protection system: A long-term birth cohort study from infancy to adulthood using administrative data. Child Abuse Negl. 2020;107:104518.

181. Jadda S, Abidli Z, Hami H, Rhalem N, Sefiani H, Soulaymani A, et al. Epidemiology and risk factors related to medication errors in moroccan children. Research Journal of Pharmacy and Technology. 2020;13(11):5355-9.

182. Kane-Gill SL, Van Den Bos J, Handler SM. Adverse drug reactions in hospital and ambulatory care settings identified using a large administrative database. Ann Pharmacother. 2010;44(6):983-93.

183. Kobya Bulut H, Canan Demirbağ B, Kahriman İ. The Investigation of Unplanned Hospital Visits and Admissions in the Children Receiving Chemotherapy. Cancer Invest. 2019;37(4-5):209-15.

184. Koliou M, Ioannou C, Andreou K, Petridou A, Soteriades ES. The epidemiology of childhood poisonings in Cyprus. Eur J Pediatr. 2010;169(7):833-8.

185. Konuru V, Naveena B, Sneha Reddy E, Vivek BC, Shravani G. A Prospective Study on Hospitalization due to Drug-related Problems in a Tertiary Care Hospital. J Pharm Bioallied Sci. 2019;11(4):328-32.

186. Kumar M, Sharan S, Roy SS, Kumari N, Hameed S, Mishra H, et al. Drug-Related Problems and Their Preventability among Admitted Patients in Paediatrics Department of a Tertiary Care Institute from Eastern India-A Prospective Study. International Journal of Pharmaceutical Sciences Review and Research. 2022;76(1):89-95.

187. Kumhar JN, Acharya RP, Chhimpa VK. Prevalence of Adverse Drug Reactions in Patients of Internal Medicine Department in a Tertiary Care Hospital in West Rajasthan: A Prospective Study. International Journal of Pharmaceutical and Clinical Research. 2023;15(3):697-700.

188. Kunac DL, Kennedy J, Austin N, Reith D. Incidence, preventability, and impact of Adverse Drug Events (ADEs) and potential ADEs in hospitalized children in New Zealand: a prospective observational cohort study. Paediatr Drugs. 2009;11(2):153-60.

189. Lim M, Shulman DS, Roberts H, Li A, Clymer J, Bona K, et al. Off-label prescribing of targeted anticancer therapy at a large pediatric cancer center. Cancer Med. 2020;9(18):6658-66.

190. Lombardi N, Crescioli G, Bettiol A, Tuccori M, Capuano A, Bonaiuti R, et al. Italian Emergency Department Visits and Hospitalizations for Outpatients' Adverse Drug Events: 12-Year Active Pharmacovigilance Surveillance (The MEREAFaPS Study). Front Pharmacol. 2020;11:412.

191. Lovegrove MC, Geller AI, Fleming-Dutra KE, Shehab N, Sapiano MRP, Budnitz DS. US Emergency Department Visits for Adverse Drug Events From Antibiotics in Children, 2011-2015. J Pediatric Infect Dis Soc. 2019;8(5):384-91.

192. Lovegrove MC, Mathew J, Hampp C, Governale L, Wysowski DK, Budnitz DS. Emergency hospitalizations for unsupervised prescription medication ingestions by young children. Pediatrics. 2014;134(4):e1009-16.

193. Lovegrove MC, Weidle NJ, Budnitz DS. Ingestion of Over-the-Counter Liquid Medications: Emergency Department Visits by Children Aged Less Than 6 Years, 2012-2015. Am J Prev Med. 2019;56(2):288-92.

194. Maior M, Osorio-de-Castro CGS, Andrade CLT. Demographics, deaths and severity indicators in hospitalizations due to drug poisoning among children under age five in Brazil. Rev Bras Epidemiol. 2020;23:e200016.

195. Matalová P, Poruba M, Wawruch M, Ondra P, Urbánek K. Acute medication poisoning causing hospital admissions in childhood: a 3-year prospective observational single-center study. Physiol Res. 2019;68(Suppl 1):S31-s8.

196. Mishra S, Ramkumar TV, Biswas AK, Panigrahi S. Childhood poisoning, a rising epidemic in developing nations: Large single centre study. Journal of Nepal Paediatric Society. 2017;37(2):117-21.

197. Morales-Ríos O, Jasso-Gutiérrez L, Reyes-López A, Garduño-Espinosa J, Muñoz-Hernández O. Potential drug-drug interactions and their risk factors in pediatric patients admitted to the emergency department of a tertiary care hospital in Mexico. PLoS One. 2018;13(1):e0190882.

198. Neininger MP, Wehr R, Kiesel LM, Neubert A, Kiess W, Bertsche A, et al. Adverse Drug Reactions at Nonelective Hospital Admission in Children and Adolescents: Comparison of 4 Causality Assessment Methods. J Patient Saf. 2022;18(4):318-24.

199. Oumar AA, Diallo K, Dembélé JP, Samaké L, Sidibé I, Togo B, et al. Adverse drug reactions to antiretroviral therapy: prospective study in children in sikasso (mali). J Pediatr Pharmacol Ther. 2012;17(4):382-8.

200. Peter JV, Varghese GH, Alexander H, Tom NR, Swethalekshmi V, Truman C, et al. Patterns of Adverse Drug Reaction in the Medical Wards of a Teaching Hospital: A Prospective Observational Cohort Study. Curr Drug Saf. 2016;11(2):164-71.

201. Post S, Spiller HA, Casavant MJ, Chounthirath T, Smith GA. Buprenorphine Exposures Among Children and Adolescents Reported to US Poison Control Centers. Pediatrics. 2018;142(1).

202. Remesh A, Balan A, Gnanadurai A. A cross-sectional study of surveillance of adverse drug reactions in inpatient departments of a tertiary care hospital. J Basic Clin Physiol Pharmacol. 2014;25(1):125-30.

203. Robert S, Ménétré S, Schweitzer C, Demoré B. Observational study of drug-related problems and clinical pharmacists' interventions in a French paediatric hospital. Eur J Hosp Pharm. 2021;28(e1):E85-E91.

204. Sadeghi-Bojd S, Khajeh A. Chronological variations of children poisoning causes in zahedan, South of iran. Int J High Risk Behav Addict. 2014;3(3):e19223.

205. Satyanarayana VVV, Purushothaman S, Chandipriya B. Clinical Spectrum of Cutaneous Adverse Drug Reactions in Pediatric Population in East Coast of Andhra Pradesh: An Observational Study. INDIAN JOURNAL OF PAEDIATRIC DERMATOLOGY. 2021;22(1):37-42.
206. Seden K, Kirkham JJ, Kennedy T, Lloyd M, James S, McManus A, et al. Cross-sectional study of prescribing errors in patients admitted to nine hospitals across North West England. BMJ Open. 2013;3(1).
207. Shehab N, Lovegrove MC, Geller AI, Rose KO, Weidle NJ, Budnitz DS. US Emergency Department Visits for Outpatient Adverse Drug Events, 2013-2014. Jama. 2016;316(20):2115-25.
208. Silva D, Colvin L, Hagemann E, Stanley F, Bower C. Children diagnosed with attention deficit disorder and their hospitalisations: population data linkage study. Eur Child Adolesc Psychiatry. 2014;23(11):1043-50.
209. Silva LT, Modesto ACF, de Oliveira RA, Amara RG, Lopes FM. Hospitalizations and adverse drug events in the Brazilian unified health system: a ten-year retrospective analysis of routine data. Revista de Saude Publica. 2022;56.
210. Smith MD, Spiller HA, Casavant MJ, Chounthirath T, Brophy TJ, Xiang H. Out-of-hospital medication errors among young children in the United States, 2002-2012. Pediatrics. 2014;134(5):867-76.
211. Souza A, Jr., Santos D, Fonseca S, Medeiros M, Batista L, Turner M, et al. Toxic excipients in medications for neonates in Brazil. Eur J Pediatr. 2014;173(7):935-45.
212. Srivastava VK, Singh D, Kumari S, Kumari S, Kumar M, Pandey BL, et al. STUDY OF ADVERSE DRUG REACTIONS AT TERTIARY HEALTH CARE CENTRE OF SOUTHWEST BIHAR. International Journal of Academic Medicine and Pharmacy. 2023;5(3):1854-9.
213. Tartof SY, Tseng HF, Liu IL, Qian L, Sy LS, Hechter RC, et al. Inpatient admission for febrile seizure and subsequent outcomes do not differ in children with vaccine-associated versus non-vaccine associated febrile seizures. Vaccine. 2014;32(48):6408-14.
214. Tolska HK, Takala AJ, Jero J. Peritonsillar infiltration of lidocaine with adrenaline is associated with increased risk of secondary post-tonsillectomy haemorrhage. J Laryngol Otol. 2018;132(10):911-22.
215. Tripathy R, Das S, Das P, Mohakud NK, Das M. Adverse Drug Reactions in the Pediatric Population: Findings From the Adverse Drug Reaction Monitoring Center of a Teaching Hospital in Odisha (2015-2020). Cureus. 2021;13(11):e19424.
216. Tse Y, Tuthill D. Incidence of paediatric 10-fold medication errors in Wales. Arch Dis Child. 2021;106(7):656-61.
217. Tsilimingras D, Natarajan G, Bajaj M, Agarwal P, Lua J, Deriemacker A, et al. Postdischarge Adverse Events Among Neonates Admitted to the Neonatal Intensive Care Unit. J Patient Saf. 2022;18(5):462-9.
218. van der Hooft CS, Sturkenboom MC, van Grootheest K, Kingma HJ, Stricker BH. Adverse drug reaction-related hospitalisations: a nationwide study in The Netherlands. Drug Saf. 2006;29(2):161-8.
219. Vora MB, Trivedi HR, Shah BK, Tripathi CB. Adverse drug reactions in inpatients of internal medicine wards at a tertiary care hospital: A prospective cohort study. J Pharmacol Pharmacother. 2011;2(1):21-5.
220. Wilson K, Ducharme R, Hawken S. Association between socioeconomic status and adverse events following immunization at 2, 4, 6 and 12 months. Hum Vaccin Immunother. 2013;9(5):1153-7.
221. Wilson K, Hawken S. Incidence of adverse events in premature children following 2-month vaccination. Hum Vaccin Immunother. 2012;8(5):592-5.
222. Yismaw MB, Adam H, Engidawork E. Identification and Resolution of Drug-Related Problems among Childhood Cancer Patients in Ethiopia. J Oncol. 2020;2020:6785835.
223. Oumar AA, Katile D, Toure A, Ba S, Sylla M, Dao S. Adverse drug reactions in children medical inpatients in Mali: Prospective observational study. Drug Saf. 2010;33(10):909.
224. Oumar A, Diallo F, Samake L, Sidibé I, Maiga B, Sylla M, et al. Adverse drug reactions in children medical in patients in Mali: Prospective observational study. Fundamental and Clinical Pharmacology. 2011;25:9.
225. Carvalho IV, Visacri MB, Santi D, Reis MC, Queiroz RA, Moriel P, et al. Drug-related problems cause many admissions to a Brazilian hospital paediatric emergency unit: A prospective and observational study. European Journal of Hospital Pharmacy: Science and Practice. 2012;19(2):97.
226. Thornton SL, Pchelnikova J, Cantrell FL. A review of pediatric exposures to anti-dementia drugs reported to a state wide poison control system. Clinical Toxicology. 2012;50(7):599.
227. Roy A, Lee MK, Nalliah RP, Rampa S, Allareddy V, Allareddy V. Adverse drug events in hospitalized children: Estimates from the nationwide inpatient sample 2003-2010. Pediatric Critical Care Medicine. 2013;14(9):924.
228. Vighi G, Prestini L, Rotondo F, Di Sessa F. ADRs in emergency department: Incidence and characteristics of serious reactions. Drug Saf. 2013;36(9):865.
229. Lee MH, Chung EH, Lim YS. The clinical characteristics of children and adolescents with anaphylaxis who visited 139 ERS in Korea in 2012. Journal of Allergy and Clinical Immunology. 2014;133(2):AB23.
230. Lombardi N, Moschini M, Bonaiuti R, Calvani AM, Parrilli M, Innocenti L, et al. Children adverse drug reactions in emergency department: An analysis of mereafaps study national database. Drug Saf. 2015;38(10):1035-6.

231. Zeribi B DE, Bounes V, Claudet I, Durrieu G, Rousseau V, et al. Incidence of Adverse Drug Reactions (ADRs) for children in an emergency medical dispatching centre in a pediatric university hospital. Fundamental and Clinical Pharmacology. 2015:29:68.

232. Chung CP, Callahan ST, Cooper W, Dupont W, Murray K, Hall K, et al. Opioid prescriptions and adverse reactions in children and adolescents without serious diseases. Arthritis and Rheumatology. 2016;68:471-2.

233. Awizoba A, Potchoo Y, Nyansa A, Aoun O, Lahaye FM, Rapp C. Self-medication in hospitalized children aged less than 15 years in Lome, Togo. Tropical Medicine and International Health. 2017;22:315-6.

234. Balme K, Stephen C. Morbidity and mortality of paediatric poisonings at a children's hospital in South Africa. Clinical Toxicology. 2019;57(6):544-5.

235. Bio L, Schwenk HT, Wise R, Yeung J, Qureshi L, Sutherland S. Identification of acute kidney injury in pediatric cystic fibrosis patients. Pediatr Pulmonol. 2019;54:284.

236. Troiano A, Bondone C, Carmellino V, Castagno E, Vermena A, Urbino AF. Out-of-hospital medication errors in children: A 6-year retrospective study. Clinical Toxicology. 2019;57(6):545-6.

237. Tse Y, Tuthill D. Tenfold medication errors in children - Welsh paediatric surveillance unit study 2017-9. Archives of Disease in Childhood. 2020;105(9).

238. Al-Tajir GK, Kelly WN. Epidemiology, comparative methods of detection, and preventability of adverse drug events. Ann Pharmacother. 2005;39(7-8):1169-74.

239. Patel H, Bell D, Molokhia M, Srishanmuganathan J, Patel M, Car J, et al. Trends in hospital admissions for adverse drug reactions in England: analysis of national hospital episode statistics 1998-2005. BMC Clin Pharmacol. 2007;7:9.

240. Schillie SF, Shehab N, Thomas KE, Budnitz DS. Medication overdoses leading to emergency department visits among children. Am J Prev Med. 2009;37(3):181-7.

241. Phan H, Leder M, Fishley M, Moeller M, Nahata M. Off-label and unlicensed medication use and associated adverse drug events in a pediatric emergency department. Pediatr Emerg Care. 2010;26(6):424-30.

242. Morales Ríos O, Jasso Gutiérrez L, Talavera JO, Téllez-Rojo MM, Olivar López V, Garduño Espinosa J, et al. A comprehensive intervention for adverse drug reactions identification and reporting in a Pediatric Emergency Department. Int J Clin Pharm. 2016;38(1):80-7.

243. Le J NT, Law A, Hodding, J. Retrospective analysis of adverse drug reactions in pediatrics over a 10-year period. Pharmacotherapy. 2005;25(10):1432.

244. Sharek PJ, Classen D. The incidence of adverse events and medical error in pediatrics. Pediatr Clin North Am. 2006;53(6):1067-77.

245. Cope JU, Morrison AE, Samuels-Reid J. Adolescent use of insulin and patient-controlled analgesia pump technology: a 10-year Food and Drug Administration retrospective study of adverse events. Pediatrics. 2008;121(5):e1133-8.

246. Cope JU, Samuels-Reid JH, Morrison AE. Pediatric use of insulin pump technology: a retrospective study of adverse events in children ages 1-12 years. J Diabetes Sci Technol. 2012;6(5):1053-9.

247. Asturias EJ, Contreras-Roldan IL, Ram M, Garcia-Melgar AJ, Morales-Oquendo V, Hartman K, et al. Post-authorization safety surveillance of a liquid pentavalent vaccine in Guatemalan children. Vaccine. 2013;31(49):5909-14.

248. Smyth RL, Peak M, Turner MA, Nunn AJ, Williamson PR, Young B, et al. ADRIC: Adverse Drug Reactions In Children – a programme of research using mixed methods. ADRIC: Adverse Drug Reactions In Children – a programme of research using mixed methods. Southampton (UK): NIHR Journals Library; 2014.

249. Alqadheeb E, Alblowi F, Alolayet R, AlSadaawi A, Alharbi M, Mobarak A. A Retrospective Analysis of Adverse Drug Events Leading to Hospitalization or Prolonged Hospitalization in Saudi Arabia. Drug Saf. 2022;45(10):1227-8.

250. AlQadheeb EK, Alblowi F, Alolayet RI, Al-Sadaawi A, Alharbi M, Al Shahrani M. A retrospective analysis of adverse drug events leading to hospitalization or prolonged hospitalization in Saudi Arabia using the Saudi food and drug authority (SFDA) spontaneous reporting system. Pharmacoepidemiology and Drug Safety. 2022;31:459.

251. Schirm E, Tobi H, van Puijenbroek EP, Monster-Simons MH, de Jong-van den Berg LT. Reported adverse drug reactions and their determinants in Dutch children outside the hospital. Pharmacoepidemiol Drug Saf. 2004;13(3):159-65.

252. Rosli R, Dali AF, Aziz NA, Ming LC, Manan MM. Reported Adverse Drug Reactions in Infants: A Nationwide Analysis in Malaysia. Front Pharmacol. 2017;8:30.

253. Botzenhardt S, Sing CW, Wong IC, Chan GC, Wong LY, Felisi M, et al. Safety Profile of Oral Iron Chelator Deferiprone in Chinese Children with Transfusion-Dependent Thalassaemia. Curr Drug Saf. 2016;11(2):137-44.

254. Del Pozzo-Magaña BR, Lazo-Langner A, Rieder MJ. Health-related quality of life in children with cutaneous adverse drug reactions. Pediatr Dermatol. 2017;34(6):e341-e2.

255. Karimzadeh P, Bakrani V. Antiepileptic drug-related adverse reactions and factors influencing these reactions. Iran J Child Neurol. 2013;7(3):25-9.

256. Leroy S, Marc E, Bavoux F, Tréluyer JM, Gendrel D, Bréart G, et al. Hospitalization for severe bacterial infections in children after exposure to NSAIDs: a prospective adverse drug reaction reporting study. Clin Drug Investig. 2010;30(3):179-85.

257. Lombardi N, Bettiol A, Crescioli G, Ravaldi C, Bonaiuti R, Venegoni M, et al. Risk of hospitalisation associated with benzodiazepines and z-drugs in Italy: a nationwide multicentre study in emergency departments. Intern Emerg Med. 2020;15(7):1291-302.

258. Moffett BS, Kim S, Bomgaars LR. Readmissions for warfarin-related bleeding in pediatric patients after hospital discharge. Pediatr Blood Cancer. 2013;60(9):1503-6.

259. Mokhtari F, Nikyar Z, Naeini BA, Esfahani AA, Rahmani S. Adverse cutaneous drug reactions: Eight year assessment in hospitalized patients. J Res Med Sci. 2014;19(8):720-5.

260. Or F, Kim Y, Simms J, Austin SB. Taking Stock of Dietary Supplements' Harmful Effects on Children, Adolescents, and Young Adults. J Adolesc Health. 2019;65(4):455-61.

261. Singh GK, Mitra B, Arora S, Akhoon N, Verma R, Sharma P, et al. A retrospective, 5-year, clinicoepidemiological study of severe cutaneous adverse reactions (SCARs). Int J Dermatol. 2021;60(5):579-88.

262. Townsley E, Gillon J, Jimenez-Truque N, Katz S, Garguilo K, Banerjee R. Risk factors for adverse events in children receiving outpatient parenteral antibiotic therapy. Hospital Pediatrics. 2021;11(2):153-9.

263. West Z, Allen K, Monroe C, Castellino SM, Miller TP. Adverse events during induction therapy in pediatric Acute Lymphoblastic Lymphoma (ALL). Blood. 2019;134.

264. Capelle H, Marchetti J, Castets S, Marquant E, Reynaud R, Tsimaratos M, et al. Implementing clinical pharmacy practices into the management of pediatric patients with pituitary or adrenal pathology to improve medication safety and adherence. International Journal of Clinical Pharmacy. 2020;42(1):248-9.

265. Daoudi M, Ahid S, Filali H, Tazi A, Dehbi F, Hakkou F. Neurological side effects associated with métoclopramide in pediatric population. Fundamental and Clinical Pharmacology. 2012;26:103.

266. Espitia S, O M, Ramos-Guevara JD, Herrera MD, Zuñiga YC, Penagos NE, et al. Severe adverse effects of antiepileptic drugs: Description of a pediatric population in colombia. Epilepsy Currents. 2013;13:269-70.

267. Menniti I, Rossi R, Traversa G, Da Cas R, Rafaniello C, Mores N, et al. Stevens-Johnson syndrome and drugs and vaccines: A case-control study in children. Drug Saf. 2011;34(10):965.

268. Santuccio C, Menniti I, Tartaglia L, Da Cas R, Trotta F, Traversa G. Adverse events of special interest and pneumococcal vaccination. Drug Saf. 2011;34(10):889.

269. Santos RP BG P-VE. Drug-related hospitalization among pediatric patients in a tertiary hospital Santo Tomas. Journal of Medicine (United States). 2000;49:141-52.
