# Supplementary material for: The epidemiology of drug-related hospital admissions in paediatrics – a systematic review
Source: Arch Public Health. 2024 Jun 4;82:81. doi: 10.1186/s13690-024-01295-4 (PMC11149243; doi:10.1186/s13690-024-01295-4)
Supplement: Supplementary file 5 — Additional file 5. Study characteristics and descriptive results. [file 13690_2024_1295_MOESM5_ESM.pdf]

Table 1a. Study characteristics and descriptive results of STUDIES WITH A BROADER CONCEPT OF 'ADVERSE DRUG EVENTS', 'WITH INTENSIVE MONITORING'

Studies; End of data collection [year]; Country (SDI)	General information on the study design			Study population: Inclusion criteria			Methods of data collection			Descriptive outcomes on the paediatric patients	
	**Setting**<br>*1) Inpatient hospital departments*<br>*2) Emergency departments*<br>(multicentre if applicable)	**Temporal perspective:**<br>*1) Prospective during clinical treatment*<br>*2) Retrospective (chart review)*<br>*3) Retrospective (database);*<br>Study period [months]	**Definition of 'drug-related problems'**	**Selection by …**<br>**clinical condition**	**admissions**<br>a) Circumstances of admission<br>○ *Admission via ED*<br>○ *Only acutely admitted inpatients;*<br>b) Minimum duration of stay;<br>c) Repeated ad-missions: Explicitly<br>○ *Included*<br>○ *Excluded*	**age or defined age categories**	**Methods of …**<br>**monitoring DRP:**<br>*Intensively monitored (chart review / assessment) by study staff vs. routinely monitored (origin of collected data from routine healthcare processes (→ patient charts / discharge diagnosis / E-codes))*	**causality assessment (between drug and problem)**<br>**Level of causality**<br>*1) ≥ probable / certain*<br>*2) ≥ possible / likely*<br>*3) ≥ conditional / doubtful*<br>*4) else*	**prevent-ability assessment**	**a) Total number of evaluated paediatric admissions or visits**<br>**b) Age of the total paediatric study population [years]**<br>**c) Gender % male**	**Information on clinical characteristics**<br>**a) Length of hospital stay (LOS) [days]**<br>**b) Number of medications per admissions**<br>**c) Main drug (classes) used (in more than 10% of admissions)**
Buajordet 2002[1]; [1996]; Norway (0.837)	1) General paediatric wards	1) Prospective; [5 months]	ADE, defined by study author	Exclusion: Patients of the neonatal unit	a) NR;<br>b) NR;<br>c) Explicitly included	≤ 15 years (neonates excluded)	Intensively monitored + spontaneous reporting	Naranjo algorithm[56];<br>2) ≥ possible / likely	NA	a) 919<br>b) Mean age (range): 2.7 (0 - 16)<br>c) 55%	a) NR<br>b) NR<br>c) Paracetamol (53%), Salbutamol (50%), Racemic adrenaline (42%), Phenoxymethylpenicillin (19%), Ampicillin (17%), Diazepam (13%), Thiopentone (12%), Amoxicillin (117%), Midazolam (11%), Nasal decongestants (11%)
Easton-Carter 2003[2]; [1999]; Australia (0.774)	2) Paediatric emergency departments (3 centres)	1) Prospective; [4.2 months]	ADE, (Strand et al[47])	Exclusions: Oncology + trauma / surgical patients	a) via ED;<br>b) NR;<br>c) NR	≤ 17 years	Intensively monitored	Expert judgement, see Dartnell et al[58];<br>2) ≥ possible / likely	NA	a) 8 601<br>b) Mean age (SD): 4.1 (2) median: 2.0<br>c) 56.8%	a) NR<br>b) NR<br>c) NR
Easton 2004[3]; [1999]; Australia (0.774)	1) Paediatric wards (2 centres)	1) Prospective; [5.1 months]	ADE, (Strand et al[47])	Exclusions: Oncology + trauma / surgical patients	a) Acutely admitted;<br>b) NR;<br>c) NR	≤ 17 years	Intensively monitored	Expert judgement, see Dartnell et al[58];<br>2) ≥ possible / likely	Algorithm (according Schumock et al[64])	a) 2 933<br>b) NR<br>c) NR	a) NR<br>b) Week prior to admission: Mean: 2 drugs; median 2.0 drugs, ± SD 1.4)<br>c) NR
Sikdar 2010[4 a]; [2007]; Canada (0.843)	2) Paediatric emergency department	2) Retrospective; [12 months]	ADE, (Nebeker et al[48])	Exclusions: Trauma / surgical patients + admitted due to alcohol or drug abuse, a suicide attempt	a) via ED;<br>b) NR;<br>c) Explicitly included	≤ 17 years	Screening / trigger tool - subsequently: Intensively monitored	Expert judgement, criteria defined by study authors;<br>2) ≥ possible / likely	NA	a) 65 864<br>b) Mean age (SD): 6.8 (5.3)<br>c) 51.2%	a) NR<br>b) NR<br>c) NR
Zed 2015[5]; [2012]; Canada (0.856)	2) Paediatric emergency department	1) Prospective; [12 months]	ADE, (Hepler and Strand[49])	NR	a) via ED;<br>b) NR;<br>c) Explicitly included	≤ 18 years	Intensively monitored	Naranjo algorithm[56] + WHO classification[57];<br>1) ≥ probable / certain	NA	a) 2 028<br>b) Mean age (SD): 6.1 (5.0)<br>c) 52.6%	a) Median LOS (IQR): 2.0<br>b) Mean: 1.1 drugs (1.3 SD)<br>c) NR
Kang 2022[6]; [2014]; Korea (0.859)	1) Emergency departments (3 centres)	3) Retrospective (database: "NEDIS"); [6 months]	ADE, defined by study author	Exclusions: Patients with intentional overdose, drug abuse, and/or suicidal attempts	a) via ED;<br>b) NR;<br>c) NR	≤ 17 years	Screening - subsequently: Intensively monitored	Expert judgement (WHO classification[57]);<br>2) ≥ possible / likely	NA	a) 13 312<br>b) NR<br>c) NR	a) NR<br>b) NR<br>c) NR
Toni 2019[7 b]; [≤ 2019]; Germany (0.898)	1) General paediatric ward	1) Prospective; [10 months]	'ADR + ME'	NR	a) NR;<br>b) min 24h;<br>c) NR	≤ 17 years	Intensively monitored	NR;<br>"detected medication error"	NR	a) 741<br>b) Median age (IQR): 7 (1 - 13)<br>c) NR	a) Median LOS (IQR) = 2 (2 - 4)<br>b) NR<br>c) NR
Neubert 2023[8]; [2020]; Germany (0.898)	1) Paediatric wards (12 centres)	1) Prospective; [24 months]	ADE, (Möller and Aly[50])	Exclusions: Oncology patients	a) Acutely admitted;<br>b) NR;<br>c) NR	≤ 17 years	Trigger tool - subsequently: Intensively monitored	Expert judgement (WHO classification[57]) + consensus of 2 judgements;<br>2) ≥ possible / likely	NA	a) 2 497<br>b) NR<br>c) NR	a) NR<br>b) NR<br>c) NR

Table 1b. Study characteristics and descriptive results of STUDIES WITH A BROADER CONCEPT OF 'ADVERSE DRUG EVENTS', 'BASED ON ROUTINE MONITORING'

Studies;<br>End of data collection [year];<br>Country (SDI)	General information on the study design			Study population: Inclusion criteria			Methods of data collection			Descriptive outcomes on the paediatric patients	
	Setting<br>*1) Inpatient hospital departments*<br>*2) Emergency departments*<br><br>(multicentre if applicable)	Temporal perspective:<br>*1) Prospective during clinical treatment*<br>*2) Retrospective (chart review)*<br>*3) Retrospective (database);*<br>Study period [months]	Definition of 'drug-related problems'	Selection by …<br>clinical condition	admissions<br>a) Circumstances of admission<br>○ *Admission via ED*<br>○ *Only acutely admitted inpatients;*<br>b) Minimum duration of stay;<br>c) Repeated ad-missions: Explicitly<br>○ *Included*<br>○ *Excluded*	age or defined age categories	Methods of …<br>monitoring DRP:<br>*Intensively monitored (chart review / assessment) by study staff vs. routinely monitored (origin of collected data from routine healthcare processes (→ patient charts / discharge diagnosis / E-codes))*	causality assessment (between drug and problem)<br>Level of causality<br>1) *≥ probable / certain*<br>2) *≥ possible / likely*<br>3) *≥ conditional / doubtful*<br>4) *else*	prevent-ability assessment	a) Total number of evaluated paediatric admissions or visits<br>b) Age of the total paediatric study population [years]<br>c) Gender<br>% male	Information on clinical characteristics<br>a) Length of hospital stay (LOS) [days]<br>b) Number of medications per admissions<br>c) Main drug (classes) used (in more than 10% of admissions)
Temple 2004[9] [a];<br>[1999];<br>US (0.794)	1) Inpatient hospital departments	3) Retrospective (database "hospital´s surveillance programme");<br>[72 months]	ADR + (un-) intentional overdosage (including prescribing errors)	NR	a) NR;<br>b) NR;<br>c) NR	'Paediatric'	Voluntary ADR report/ routinely detected by quality improvement staff/ hospital pharmacy (medical records, discharge coding); subsequently chart review	Naranjo algorithm[56];<br>2) ≥ possible / likely	NA	a) 65 864<br>b) NR<br>c) NR	a) NR<br>b) NR<br>c) NR
Bourgeois 2009[10] [a];<br>[2005];<br>US (0.812)	2) Emergency departments and outpatient clinics (multicentre)	3) Retrospective (database: "NAMCS" and "NHAMCS");<br>[132 months]	ADE, (Bates et al[51])	NR	a) via ED;<br>b) NR;<br>c) NR	≤ 17 years	Routine diagnostic in clinical practice	NR	NA	a) NR<br>b) NR<br>c) NR	a) NR<br>b) NR<br>c) NR
Cohen 2008[11] [a];<br>[2005];<br>US (0.812)	2) Emergency departments (multicentre)	3) Retrospective (database: "NEISS-CADES)";<br>[24 months]	ADE, defined by study author	NR	a) via ED;<br>b) NR;<br>c) NR	≤ 18 years	Identification and classification using routine data	NR	NA	a) NR<br>b) NR<br>c) NR	a) NR<br>b) NR<br>c) NR
Tundia 2011[12];<br>[2006];<br>US (0.811)	1) Inpatient hospital departments (multicentre)	3) Retrospective (database: "2006 KID");<br>[12 months]	ADE, (Elixhauser et al[52])	NR	a) NR;<br>b) NR;<br>c) NR	≤ 20 years	Routine diagnostic in clinical practice	NR	NA	a) 7 558 812<br>b) Mean age (SE): 4.6 (0.04)<br>c) 46.8%	a) Mean LOS: 3.7<br>b) NR<br>c) NR
Feinstein 2014[13];<br>[2010];<br>US (0.832)	2) Emergency departments (multicentre)	3) Retrospective (database: "National Emergency Department Sample");<br>[60 months]	"ADE, without wrong sub-stance ad-ministration, poisoning, intentional self-harm, or use of illicit substances"	Exclusion: Oncology patients	a) via ED;<br>b) NR;<br>c) Explicitly excluded	≤ 17 years	Routine diagnostic in clinical practice	NR	NA	a) NR<br>b) Median age 5 - 9 years (age categories distribution: <1 (12.1%), 1 - 4 (29.0%), 5 - 9 (18,5%), 10 - 18 (40.4%))<br>c) 51.8%	a) NR<br>b) NR<br>c) NR
Romano-Lieber 2011[14] [b];<br>[≤ 2011];<br>Brazil (0.597)	2) Paediatric emergency department	2) Retrospective;<br>[NR]	ADE, defined by study author	NR	a) via ED;<br>b) NR;<br>c) NR	'Paediatric'	Routine diagnostic in clinical practice	NR	NA	a) 23 286<br>b) NR<br>c) NR	a) NR<br>b) NR<br>c) NR
Silva 2017[16];<br>[2014];<br>Brazil (0.616)	1) Inpatient hospital departments (Paediatrics as subgroup) (multicentre)	3) Retrospective (database: "Hospital Information System database, DATASUS");<br>[36 months]	ADE, defined by study author	NR	a) NR;<br>b) NR;<br>c) NR	≤ 19 years	Routine diagnostic in clinical practice	NR	NA	a) 706 092<br>b) NR<br>c) NR	a) NR<br>b) NR<br>c) NR
Rosafio 2017[15] [a];<br>[2014];<br>Italy (0.789)	2) Emergency department	2) Retrospective;<br>[96 months]	ADE, (Hepler and Strand[49])	NR	a) via ED;<br>b) NR;<br>c) NR	≤ 13 years	Identification and classification using routine data	Naranjo algorithm[56] + WHO classification[57];<br>1) ≥ probable / certain	NA	a) 147 643<br>b) NR<br>c) NR	a) NR<br>b) NR<br>c) NR

Table 1c. Study characteristics and descriptive results of STUDIES WITH A NARROWER CONCEPT OF 'ADVERSE DRUG REACTIONS', 'WITH INTENSIVE MONITORING'

**Studies;<br>End of data collection [year];<br>Country (SDI)**	**General information on the study design**			**Study population: Inclusion criteria**			**Methods of data collection**			**Descriptive outcomes on the paediatric patients**	
	**Setting**<br>***1) Inpatient hospital departments***<br>***2) Emergency departments***<br><br>**(multicentre if applicable)**	**Temporal perspective:**<br>***1) Prospective during clinical treatment***<br>***2) Retrospective (chart review)***<br>***3) Retrospective (database);***<br>**Study period [months]**	**Definition of 'drug-related problems'**	**Selection by …**<br>**clinical condition**	**admissions**<br>**a) Circumstances of admission**<br>○ ***Admission via ED***<br>○ ***Only acutely admitted inpatients;***<br>**b) Minimum duration of stay;**<br>**c) Repeated ad-missions: Explicitly**<br>○ ***Included***<br>○ ***Excluded***	**age or defined age categories**	**Methods of …**<br>**monitoring DRP:**<br>***Intensively monitored (chart review / assessment) by study staff vs. routinely monitored (origin of collected data from routine healthcare processes (→ patient charts / discharge diagnosis / E-codes))***	**causality assessment (between drug and problem)**<br>**Level of causality**<br>**1)** ***≥ probable / certain***<br>**2)** ***≥ possible / likely***<br>**3)** ***≥ conditional / doubtful***<br>**4)** ***else***	**prevent-ability assessment**	**a) Total number of evaluated paediatric admissions or visits**<br>**b) Age of the total paediatric study population [years]**<br>**c) Gender % male**	**Information on clinical characteristics**<br>**a) Length of hospital stay (LOS) [days]**<br>**b) Number of medications per admissions**<br>**c) Main drug (classes) used (in more than 10% of admissions)**
Pouyanne 2000[17];<br>[1998];<br>France (0.773)	1) Inpatient hospital departments (Paediatrics as subgroup) (multicentre)	1) Prospective;<br>[0.5 months]	ADR, WHO definition[53]	NR	a) NR;<br>b) NR;<br>c) NR	≤ 15 years	Intensively monitored	NR	NA	a) 525<br>b) NR<br>c) NR	a) NR<br>b) NR<br>c) NR
Jonville-Béra 2002[18];<br>[1998];<br>France (0.773)	1) Various inpatient paediatric hospital departments	1) Prospective;<br>[0.2 months]	'ADR'	Exclusion:<br>Accident and emergency department patients	a) NR;<br>b) min 24h;<br>c) NR	'Paediatric'	Intensively monitored	Algorithm, see Bégaud et al[59];<br>"suspected relationship"	NA	a) 260<br>b) Mean age (SD): 6.3 (5.1)<br>c) 60.4%	a) Median LOS (range): 3 (1 - 70)<br>b) Before admission:<br>Median: 2 drugs (range 1 – 11)<br>During admission:<br>Median: 3 drugs (range 1 – 14)<br>c) NR
Haffner 2005[19];<br>[2001];<br>Germany (0.853)	1) General paediatric wards, PICU	1) Prospective;<br>[1.5 months]	ADR, WHO definition[53]	Exclusion:<br>Oncology patients<br>Inclusion:<br>General ward + ICU patients	a) NR;<br>b) NR;<br>c) Explicitly included	'Paediatric'	Two methods:<br>1) Intensively monitored (incl. interviews)<br>2) Trigger tool + subsequently intensive assessment	Expert judgement, WHO classification[57];<br>2) ≥ possible / likely	NA	a) 411<br>b) No pooled data<br>Cohort 1:<br>Mean age (SD): 4.6 (5.1)<br>Median age: 2.2<br>Cohort 2:<br>Mean age (SD): 5.0 (5.3)<br>Median age 2.4<br>c) 58.9%	a) No pooled data<br>Cohort 1:<br>Mean LOS (SD): 7.2 (13.2)<br>Median LOS: 5<br>Cohort 2:<br>Mean LOS (SD): 7.0 (13.6)<br>Median LOS 4.0<br>b) NR<br>c) NR
Weiss 2002[20];<br>[≤ 2002];<br>Germany (0.856)	1) Paediatric infectious diseases department	1) Prospective;<br>[8 months]	ADR, WHO definition[53]	Inclusion:<br>Patients admitted to paediatric isolation ward	a) NR;<br>b) NR;<br>c) NR	'Paediatric'	Computerised automatically generated laboratory signals + Intensively monitored	Algorithm, see Evans et al[60] = adapted Naranjo algorithm[56];<br>2) ≥ possible / likely	NA	a) 214<br>b) Median age (range):<br>8.6 (0.1 - 35)<br>c) NR	a) Median LOS (range): 6.9 (1 - 187)<br>b) NR<br>c) NR
Lamabadusuriya 2003[21];<br>[2002];<br>Sri Lanka (0.582)	1) All medical inpatient hospital departments	1) Prospective;<br>[11 months]	ADR, WHO definition[53]	Inclusion:<br>Medical ward patients	a) NR;<br>b) NR;<br>c) NR	'Paediatric'	Intensively monitored	Naranjo algorithm[56];<br>2) ≥ possible / likely	NA	a) 39 625<br>b) NR<br>c) NR	a) NR<br>b) NR<br>c) NR
Fattahi 2005[22];<br>[2004];<br>Iran (0.562)	1) Paediatric department of Infectious Diseases	1) Prospective;<br>[5 months]	ADR, WHO definition[53]	Inclusion:<br>Infectious diseases department patients	a) NR;<br>b) min 24h;<br>c) Explicitly excluded	≤ 14 years	Intensively monitored	Expert judgement (WHO classification[57]);<br>3) ≥ conditional / doubtful	NA	a) 404<br>b) NR<br>c) NR	a) Mean LOS: 6.5<br>(2611 patient-days / 404 patients)<br>b) Mean: 3.4 drugs<br>(SD 2.95; range: 0 – 20)<br>c) NR
Oshikoya 2007[23 b];<br>[2006];<br>Nigeria (0.402)	1) Inpatient department for paediatric medical conditions	2) Retrospective;<br>30 months] +<br>1) Prospective;<br>[6 months]	'ADR'	Inclusion:<br>Various forms of paediatric medical conditions	a) NR;<br>b) NR;<br>c) NR	'Paediatric'	Two datasets:<br>1) Routine diagnostic in clinical practice<br>2) Intensively monitored	NR	NA	a) 3 821<br>b) NR<br>c) NR	a) NR<br>b) NR<br>c) NR
Bénard-Laribière 2015[24];<br>[2007];<br>France (0.799)	1) Inpatient medical wards (Paediatrics as subgroup) (multicentre)	1) Prospective;<br>[0.5 months]	ADR, WHO definition[53]	NR	a) NR;<br>b) min 24h;<br>c) NR	≤ 15 years	Intensively monitored	Expert judgement: criteria defined by study authors;<br>≥ 'possible', according to the study authors (not formally hierarchical)	NA	a) 518<br>b) NR<br>c) NR	a) NR<br>b) NR<br>c) NR
Oshikoya 2011[25];<br>[2007];<br>Nigeria (0.412)	1) Inpatient department for paediatric medical conditions	1) Prospective;<br>[18 months]	ADR, (Edwards and Aronson[53])	NR	a) NR;<br>b) min 24h;<br>c) Explicitly excluded	'Paediatric'	Trigger tool + Intensively monitored	Algorithm, see Jones et al[61];<br>2) ≥ possible / likely	NA	a) 2 004<br>b) NR<br>c) 61.0%	a) NR<br>b) Mean: 4.2 drugs (range 2 – 10)<br>c) NR

Table 1c. Study characteristics and descriptive results of STUDIES WITH A NARROWER CONCEPT OF 'ADVERSE DRUG REACTIONS', 'WITH INTENSIVE MONITORING'

Studies;<br>End of data collection [year];<br>Country (SDI)	General information on the study design			Study population: Inclusion criteria			Methods of data collection			Descriptive outcomes on the paediatric patients	
	Setting<br>*1) Inpatient hospital departments*<br>*2) Emergency departments*<br><br>(multicentre if applicable)	Temporal perspective:<br>*1) Prospective during clinical treatment*<br>*2) Retrospective (chart review)*<br>*3) Retrospective (database);*<br>Study period [months]	Definition of 'drug-related problems'	Selection by …<br>clinical condition	admissions<br>a) Circumstances of admission<br>○ *Admission via ED*<br>○ *Only acutely admitted inpatients;*<br>b) Minimum duration of stay;<br>c) Repeated ad-missions: Explicitly<br>○ *Included*<br>○ *Excluded*	age or defined age categories	Methods of …<br>monitoring DRP:<br>*Intensively monitored (chart review / assessment) by study staff vs. routinely monitored (origin of collected data from routine healthcare processes (→ patient charts / discharge diagnosis / E-codes))*	causality assessment (between drug and problem)<br>Level of causality<br>*1) ≥ probable / certain*<br>*2) ≥ possible / likely*<br>*3) ≥ conditional / doubtful*<br>*4) else*	prevent-ability assessment	a) Total number of evaluated paediatric admissions or visits<br>b) Age of the total paediatric study population [years]<br>c) Gender % male	Information on clinical characteristics<br>a) Length of hospital stay (LOS) [days]<br>b) Number of medications per admissions<br>c) Main drug (classes) used (in more than 10% of admissions)
Gallagher 2011[26];<br>[2008];<br>UK (0.811)	1) Paediatric main hospital wards, observation ward	1) Prospective;<br>[0.5 months]	ADR,<br>(Edwards and Aronson[53])	Exclusion:<br>Patients with accidental or intentional overdose	a) Acutely admitted;<br>b) Subgroup: main ward: min 4h; observation ward: max 4h;<br>c) Explicitly included	'Paediatric'	Intensively monitored	Naranjo algorithm[56];<br>2) ≥ possible / likely	Definition (Hallas et al[65])	a) 822<br>b) NR<br>c) NR	a) NR<br>b) NR<br>c) NR
Posthumus 2012[27];<br>[2008];<br>The Netherlands (0.856)	2) Emergency departments and outpatient clinics, admitting patients to paediatric wards and ICU	1) Prospective;<br>[5 months]	ADR,<br>(Krähenbühl-Melcher et al[54])	Exclusion:<br>Patients admitted due to alcohol or drug abuse, a suicide attempt	a) via ED;<br>b) NR;<br>c) Explicitly included	≤ 18 years	Trigger tool + Intensively monitored	Naranjo algorithm[56];<br>2) ≥ possible / likely	Algorithm (Schumock et al[64])	a) 683<br>b) Median age (range): 3.2 (0 - 17.9)<br>c) 59.0%	a) NR<br>b) NR<br>c) Main drug classes: drugs for the alimentary tract and metabolism, the nervous system, and anti-infectives for systemic use
Gallagher 2012[29] / Bellis 2014[28];<br>[2009];<br>UK (0.813)	1) Paediatric main hospital wards (general and specialty care)	1) Prospective;<br>[12 months]	ADR,<br>(Edwards and Aronson[53])	Exclusion:<br>Patients with accidental or intentional overdose, misuse of medicines, elective admissions	a) Acutely admitted;<br>b) min 4h (main ward patients, exclusion of observation ward patients)<br>c) Explicitly included	'Paediatric'	Intensively monitored	Algorithm, see Liverpool ADR Causality Assessment Tool, Gallagher et al[62];<br>2) ≥ possible / likely	Definition (Hallas et al[65])	a) 8 345<br>b) Median age (IQR): 3.1 (0.8 - 9) (calculated for patients exposed to any medication in the 2 weeks prior hospitalisation on their first admission)<br>c) 58.0%	a) NR<br>b) Median 2 drugs (IQR: 1; 3) (calculated for patients exposed to any medication in the 2 weeks prior hospitalisation)<br>c) NR
Langerová 2014[30];<br>[2012];<br>Czech Republic (0.818)	1) Inpatient department of paediatrics	1) Prospective;<br>[9 months]	ADR,<br>(Edwards and Aronson[53])	NR	a) NR;<br>b) NR;<br>c) Explicitly included	≤ 19 years	Intensively monitored	Naranjo algorithm[56] + Liver-pool ADR Causality Assess-ment Tool[62] + Edwards and Aronson[53] causality assessment method;<br>2) ≥ possible / likely	NA	a) 2 903<br>b) Mean age (SD): 7.1 (5.7)<br>c) 57.3%	a) NR<br>b) NR<br>c) NR
Russom 2017[31 a];<br>[2014];<br>Eritrea (0.360)	1) Eritrean inpatient hospital departments (Paediatrics as subgroup) (multicentre)	1) Prospective;<br>[5 months]	ADR, WHO definition[53]	Exclusion:<br>Neonates, mothers admitted for delivery reasons	a) NR;<br>b) NR;<br>c) NR	≤ 15 years (neonates excluded)	Intensively monitored	Naranjo algorithm[56];<br>2) ≥ possible / likely	NA	a) 2 433<br>b) NR<br>c) NR	a) NR<br>b) NR<br>c) NR
Gholami 2015[32];<br>[2015];<br>Iran (0.649)	2) Paediatric emergency department	1) Prospective;<br>[6 months]	ADR,<br>(Edwards and Aronson[53])	Exclusion:<br>Patients with overdoses, suicide attempt, medication error	a) via ED;<br>b) min 6h;<br>c) NR	≤ 18 years	Intensively monitored	Naranjo algorithm[56] + Expert judgement (WHO classification[57]);<br>2) ≥ possible / likely	Algorithm (Schumock et al[64])	a) 658<br>b) Mean age (SD): 3.4 (3)<br>c) NR	a) NR<br>b) NR<br>c) NR
Mouton 2020[33];<br>[2015];<br>South Africa (0.664)	1) general and specialist medical, wards, high-care, intensive care unit + medical wards, high-care, intensive care unit (2 centres)	1) Prospective;<br>[1 month] +<br>2) Retrospective;<br>[1 month]	ADR,<br>(Aronson and Ferner[55])	Exclusion:<br>Patients of surgical and oncology wards, limited neonatal service<br>Inclusion:<br>Various wards + ICU patients	a) Subgroup of acutely admitted patients;<br>b) NR<br>c) Explicitly included	≤ 18 years	Trigger tool + Intensively monitored	Expert judgement (WHO classification[57]);<br>2) ≥ possible / likely	Algorithm (Schumock et al[64])	a) 1 106<br>b) Median age (IQR): 0.9 (0.1 - 3.4)<br>c) 56.0%	a) Median LOS (IQR) = 3 (2 - 6) (range: 1 - 34 days); 2)<br>b) Before admission: Median: 2 drugs (IQR 1 - 4, range 0 – 47) During admission: Median: 4 drugs (IQR 3 - 9, range 0 – 46)<br>c) Beta-adrenergic inhalants (25.41%), antipyretics (25.05%), penicillins (19.08%), other beta-lactam antibiotics (13.74%), other drugs for obstructive airway disease (11.30%)

Table 1c. Study characteristics and descriptive results of STUDIES WITH A NARROWER CONCEPT OF 'ADVERSE DRUG REACTIONS', 'WITH INTENSIVE MONITORING'

Studies;<br>End of data collection [year];<br>Country (SDI)	General information on the study design			Study population: Inclusion criteria			Methods of data collection			Descriptive outcomes on the paediatric patients	
	Setting<br>*1) Inpatient hospital departments*<br>*2) Emergency departments*<br><br>(multicentre if applicable)	Temporal perspective:<br>*1) Prospective during clinical treatment*<br>*2) Retrospective (chart review)*<br>*3) Retrospective (database);*<br>Study period [months]	Definition of 'drug-related problems'	Selection by …<br>clinical condition	admissions<br>a) Circumstances of admission<br>○ *Admission via ED*<br>○ *Only acutely admitted inpatients;*<br>b) Minimum duration of stay;<br>c) Repeated ad-missions: Explicitly<br>○ *Included*<br>○ *Excluded*	age or defined age categories	Methods of …<br>monitoring DRP:<br>*Intensively monitored (chart review / assessment) by study staff vs. routinely monitored (origin of collected data from routine healthcare processes (→ patient charts / discharge diagnosis / E-codes))*	causality assessment (between drug and problem)<br>Level of causality<br>1) *≥ probable / certain*<br>2) *≥ possible / likely*<br>3) *≥ conditional / doubtful*<br>4) *else*	prevent-ability assessment	a) Total number of evaluated paediatric admissions or visits<br>b) Age of the total paediatric study population [years]<br>c) Gender % male	Information on clinical characteristics<br>a) Length of hospital stay (LOS) [days]<br>b) Number of medications per admissions<br>c) Main drug (classes) used (in more than 10% of admissions)
Nasso 2020[34];<br>[2018];<br>Italy (0.798)	2) Paediatric Emergency Department	1) Prospective;<br>[84 months]	ADR, WHO definition[53]	NR	a) via ED;<br>b) NR;<br>c) NR	≤ 15 years	Intensively monitored	Naranjo algorithm[56];<br>2) ≥ possible / likely	Algorithm (Schumock et al[64])	a) 75 935<br>b) NR<br>c) NR	a) NR<br>b) NR<br>c) NR
Patel 2021[35];<br>[≤ 2021];<br>India (0.609)	1) Paediatric wards	1) Prospective;<br>[22 months]	ADR, WHO definition[53]	NR	a) NR;<br>b) NR;<br>c) NR	≤ 12 years	Intensively monitored	Naranjo algorithm[56] + Expert judgement (WHO classification[57]);<br>2) ≥ possible / likely	NA	a) 700<br>b) age distribution:<br>0y - 3y: 57.0%,<br>4y - 6y: 18.4%,<br>7y - 9y: 13.9%<br>10y - 12y: 10.7%<br>c) 56.9%	a) NR<br>b) NR<br>c) NR
Gupta 2023[36];<br>[2013];<br>India (0.566)	1) Paediatric ward + paediatric intensive care unit	1) Prospective;<br>[12 months]	ADR, (Edwards and Aronson[53])	Exclusion: Patients with intentional or accidental poisoning, medication errors, and drug abuse<br>Inclusion: Paediatric ward + ICU patients (without neonates, surgical patients)	a) NR;<br>b) NR;<br>c) NR	≤ 12 years (neonates excluded)	Intensively monitored	Expert judgement (WHO classification[57]);<br>1) ≥ probable / certain	NA	a) 6 026<br>b) age distribution:<br>1m - 1y: 24.8%,<br>1y - 5y: 36.1%,<br>>5y: 39.0%<br>c) NR	a) NR<br>b) NR<br>c) NR

Table 1d. Study characteristics and descriptive results of STUDIES WITH A NARROWER CONCEPT OF 'ADVERSE DRUG REACTIONS', 'BASED ON ROUTINE MONITORING'

Studies;<br>End of data collection [year];<br>Country (SDI)	General information on the study design			Study population: Inclusion criteria			Methods of data collection			Descriptive outcomes on the paediatric patients	
	Setting<br>*1) Inpatient hospital departments*<br>*2) Emergency departments*<br><br>(multicentre if applicable)	Temporal perspective:<br>*1) Prospective during clinical treatment*<br>*2) Retrospective (chart review)*<br>*3) Retrospective (database);*<br>Study period [months]	Definition of 'drug-related problems'	Selection by …<br>clinical condition	admissions<br>a) Circumstances of admission<br>○ *Admission via ED*<br>○ *Only acutely admitted inpatients;*<br>b) Minimum duration of stay;<br>c) Repeated ad-missions: Explicitly<br>○ *Included*<br>○ *Excluded*	age or defined age categories	Methods of …<br>monitoring DRP:<br>*Intensively monitored (chart review / assessment) by study staff vs. routinely monitored (origin of collected data from routine healthcare processes (→ patient charts / discharge diagnosis / E-codes))*	causality assessment (between drug and problem)<br>Level of causality<br>1) *≥ probable / certain*<br>2) *≥ possible / likely*<br>3) *≥ conditional / doubtful*<br>4) *else*	prevent-ability assessment	a) Total number of evaluated paediatric admissions or visits<br>b) Age of the total paediatric study population [years]<br>c) Gender % male	Information on clinical characteristics<br>a) Length of hospital stay (LOS) [days]<br>b) Number of medications per admissions<br>c) Main drug (classes) used (in more than 10% of admissions)
McDonnell 2002[37 a];<br>[1999];<br>US (0.794)	1) Inpatient hospital departments (Paediatrics as subgroup)	2) Retrospective;<br>[11 months]	ADR, WHO definition[53]	NR	a) NR;<br>b) NR;<br>c) NR	≤ 15 years	Identification using routine data + routine voluntary reporting using triggers	Naranjo algorithm[56];<br>1) ≥ probable / certain	NA	a) 2 046<br>b) NR<br>c) 50.9%	a) NR<br>b) NR<br>c) NR
Duczmal 2006[38 a,b];<br>[1999];<br>Poland (0.701)	1) Inpatient paediatric hospital department	2) Retrospective;<br>[84 months]	'ADR'	NR	a) NR;<br>b) NR;<br>c) NR	'Paediatric'	No clear information, probably: Routine diagnostic in clinical practice	NR	NA	a) 4 996<br>b) NR<br>c) NR	a) NR<br>b) NR<br>c) NR
Impicciatore 2002[39 b];<br>[≤ 2002];<br>Italy (0.761)	1) Inpatient paediatric hospital department	1) Prospective;<br>[9 months]	'ADR'	NR	a) NR;<br>b) NR;<br>c) NR	'Paediatric'	NR	NR	NA	a) 1 619<br>b) NR<br>c) NR	a) NR<br>b) NR<br>c) NR

Table 1d. Study characteristics and descriptive results of STUDIES WITH A NARROWER CONCEPT OF ‘ADVERSE DRUG REACTIONS’, ‘BASED ON ROUTINE MONITORING’

Studies; End of data collection [year]; Country (SDI)	General information on the study design			Study population: Inclusion criteria			Methods of data collection			Descriptive outcomes on the paediatric patients	
	Setting<br>***1) Inpatient hospital departments***<br>***2) Emergency departments***<br><br>(multicentre if applicable)	Temporal perspective:<br>***1) Prospective during clinical treatment***<br>***2) Retrospective (chart review)***<br>***3) Retrospective (database);***<br>Study period [months]	Definition of ‘drug-related problems’	Selection by …<br>clinical condition	admissions<br>a) Circumstances of admission<br>○ *Admission via ED*<br>○ *Only acutely admitted inpatients;*<br>b) Minimum duration of stay;<br>c) Repeated ad-missions: Explicitly<br>○ *Included*<br>○ *Excluded*	age or defined age categories	Methods of …<br>monitoring DRP: *Intensively monitored (chart review / assessment) by study staff vs. routinely monitored (origin of collected data from routine healthcare processes (→ patient charts / discharge diagnosis / E-codes))*	causality assessment (between drug and problem)<br>Level of causality<br>*1) ≥ probable / certain*<br>*2) ≥ possible / likely*<br>*3) ≥ conditional / doubtful*<br>*4) else*	prevent-ability assessment	a) Total number of evaluated paediatric admissions or visits<br>b) Age of the total paediatric study population [years]<br>c) Gender % male	Information on clinical characteristics<br>a) Length of hospital stay (LOS) [days]<br>b) Number of medications per admissions<br>c) Main drug (classes) used (in more than 10% of admissions)
Van der Hooft 2008[40];<br>[2003];<br>The Netherlands (0.842)	○ Information on hospitalisations from digital medical patient records of general practitioners (Paediatrics as subgroup)	3) Retrospective (database: IPCI project, general practice research database);<br>[12 months]	ADR, defined by study author	NR	a) Acutely admitted;<br>b) NR;<br>c) NR	≤ 16 years	Identification and classification using routine data	Expert judgement (WHO classification[57]);<br>1) ≥ probable / certain	NA	a) 301<br>b) NR<br>c) NR	a) NR<br>b) NR<br>c) NR
Le 2006[41];<br>[2004];<br>US (0.811)	1) Inpatient paediatric hospital departments (NICU, PICU, general paediatrics unit, haematology/ oncology)	2) Retrospective;<br>[120 months]	ADR, WHO definition[53]	NR<br><br>Inclusion: Various wards + ICU patients	a) NR;<br>b) NR;<br>c) NR	‘Paediatric’	Identification and classification using routine data	Expert judgement: criteria defined by study authors;<br>≥ conditional / doubtful	NA	a) NR<br>b) NR<br>c) NR	a) NR<br>b) NR<br>c) NR
Speranza 2008[42 a,b];<br>[2007];<br>Uruguay (0.642)	1) Inpatient paediatric hospital departments	○ NR;<br>[0.2 months]	‘ADR’	NR	a) NR;<br>b) NR;<br>c) NR	‘Paediatric’	NR	Algorithm, see Karch et al[63];<br>≥ conditional / doubtful	NA	a) 173<br>b) NR<br>c) NR	a) NR<br>b) NR<br>c) NR
Moschini 2013[43 b];<br>[2013];<br>Italy (0.787)	2) Paediatric emergency department	1) Prospective;<br>[30 months]	‘ADR’	NR	a) via ED;<br>b) NR;<br>c) NR	‘Paediatric’	Identification and classification using routine data	NR	NA	a) NR<br>b) NR<br>c) NR	a) NR<br>b) NR<br>c) NR
Mendoza 2015[44 b];<br>[≤ 2015];<br>Spain (0.756)	1) Inpatient paediatric hospital departments	2) Retrospective;<br>[132 months]	‘ADR’	NR	a) NR;<br>b) NR;<br>c) NR	≤ 17 years	Routine diagnostic in clinical practice	NR	NA	a) 73 864<br>b) NR<br>c) NR	a) NR<br>b) NR<br>c) NR
Lombardi 2018[45];<br>[2016];<br>Italy (0.794)	2) Paediatric emergency department	2) Retrospective;<br>[60 months]	‘ADR’	NR	a) via ED;<br>b) NR;<br>c) NR	‘Paediatric’	Identification and classification using routine data	Naranjo algorithm[56];<br>3) ≥ conditional / doubtful (except for vaccines: “Associable, undefined, not associable”)	NA	a) 221 528<br>b) NR<br>c) NR	a) NR<br>b) NR<br>c) NR
Morales-Ríos 2020[46];<br>[2017];<br>Mexico (0.640)	1) Inpatient paediatric hospital departments	2) Retrospective;<br>[48 months]	ADR, WHO definition[53]	NR	a) NR;<br>b) NR;<br>c) NR	‘Paediatric’	Voluntary documentation during pharmacovigilance program	Naranjo algorithm[56];<br>3) ≥ conditional / doubtful	NA	a) NR<br>b) NR<br>c) NR	a) NR<br>b) NR<br>c) NR

Table 1a. Study characteristics and descriptive results of STUDIES WITH A BROADER CONCEPT OF ‘ADVERSE DRUG EVENTS’, ‘WITH INTENSIVE MONITORING’ (yellow background)
Table 1b. Study characteristics and descriptive results of STUDIES WITH A BROADER CONCEPT OF ‘ADVERSE DRUG EVENTS’, ‘BASED ON ROUTINE MONITORING’ (orange background)
Table 1c. Study characteristics and descriptive results of STUDIES WITH A NARROWER CONCEPT OF ‘ADVERSE DRUG REACTIONS’, ‘WITH INTENSIVE MONITORING’ (green background)
Table 1d. Study characteristics and descriptive results of STUDIES WITH A NARROWER CONCEPT OF ‘ADVERSE DRUG REACTIONS’, ‘BASED ON ROUTINE MONITORING’ (blue background)

[a] study identified via citation search; [b] just abstract of the study available
Abbreviations: NR Not reported, *NA* Not applicable, *SDI* Socio-Demographic Index, considered year = End of data collection)[66], *ED* Emergency department, *DRP* Drug-related problem, *DRH* Drug-related hospitalisation, *ADE* Adverse drug event, *‘ADE’* ADE was not defined, *ADR* Adverse drug reaction, *‘ADR’* ADR was not defined, *ME* Medication error, *LOS* Length of hospital stay, *min* = Minimum, *max* Maximum, *CI* Confidence interval

Bibliography

1. Buajordet I, Wesenberg F, Brørs O, Langslet A. Adverse drug events in children during hospitalization and after discharge in a Norwegian University Hospital. *Acta Paediatr*. 2002;91(1):88-94. doi:10.1080/080352502753458021
2. Easton-Carter K, Chapman C, Brien J. Emergency department attendances associated with drug-related problems in paediatrics. *J Paediatr Child Health*. 2003;39(2):124-129. doi:10.1046/j.1440-1754.2003.00103.x
3. Easton KL, Chapman CB, Brien J anne E. Frequency and characteristics of hospital admissions associated with drug-related problems in paediatrics. *Br J Clin Pharmacol*. 2004;57(5):611-615. doi:10.1111/j.1365-2125.2003.02052.x
4. Sikdar KC, Alaghehbandan R, MacDonald D, Barrett B, Collins KD, Gadag V. Adverse drug events among children presenting to a hospital emergency department in Newfoundland and Labrador, Canada: ADVERSE DRUG EVENTS AMONG CHILDREN. *Pharmacoepidemiol Drug Saf*. 2010;19(2):132-140. doi:10.1002/pds.1900
5. Zed PJ, Black KJL, Fitzpatrick EA, et al. Medication-Related Emergency Department Visits in Pediatrics: a Prospective Observational Study. *Pediatrics*. 2015;135(3):435-443. doi:10.1542/peds.2014-1827
6. Kang MG, Lee JY, Woo SI, et al. Adverse drug events leading to emergency department visits: A multicenter observational study in Korea. Mogi M, ed. *PLOS ONE*. 2022;17(9):e0272743. doi:10.1371/journal.pone.0272743
7. Toni I, Wimmer S, Trollmann R, Rascher W, Neubert A. Drug-related hospital admissions in paediatrics-what is preventable? *Arch Dis Child*. 2019;104(6). doi:10.1136/archdischild-2019-esdppp.1
8. Neubert A, Toni I, König J, et al. A complex intervention to prevent medication-related hospital admissions—results of the stepped-wedge cluster randomized trial KiDSafe in pediatrics. *Dtsch Ärztebl Int*. Published online June 23, 2023. doi:10.3238/arztebl.m2023.0123
9. Temple ME, Robinson RF, Miller JC, Hayes JR, Nahata MC. Frequency and Preventability of Adverse Drug Reactions in Paediatric Patients: *Drug Saf*. 2004;27(11):819-829. doi:10.2165/00002018-200427110-00005
10. Bourgeois FT, Mandl KD, Valim C, Shannon MW. Pediatric Adverse Drug Events in the Outpatient Setting: An 11-Year National Analysis. *Pediatrics*. 2009;124(4):e744-e750. doi:10.1542/peds.2008-3505
11. Cohen AL, Budnitz DS, Weidenbach KN, et al. National Surveillance of Emergency Department Visits for Outpatient Adverse Drug Events in Children and Adolescents. *J Pediatr*. 2008;152(3):416-421.e2. doi:10.1016/j.jpeds.2007.07.041
12. Tundia NL, Heaton PC, Kelton CML. The national burden of E-code-identified adverse drug events among hospitalized children using a national discharge database: NATIONAL BURDEN OF PEDIATRIC ADVERSE DRUG EVENTS. *Pharmacoepidemiol Drug Saf*. 2011;20(8):866-878. doi:10.1002/pds.2150
13. Feinstein JA, Feudtner C, Kempe A. Adverse Drug Event-Related Emergency Department Visits Associated With Complex Chronic Conditions. *Pediatrics*. 2014;133(6):e1575-e1585. doi:10.1542/peds.2013-3060
14. Romano-Lieber NS, Ribeiro E. Adverse drug events leading children to emergency department, São Paulo, Brazil. *Pharmacoepidemiol Drug Saf*. 2011;20((Romano-Lieber N.S.) School of Public Health, University of São Paulo, São Paulo, SP, Brazil):S320-S321. doi:10.1002/pds.2206
15. Rosafio C, Paioli S, Del Giovane C, et al. Medication-related visits in a pediatric emergency department: an 8-years retrospective analysis. *Ital J Pediatr*. 2017;43(1):55. doi:10.1186/s13052-017-0375-7
16. Silva YDOM, Guimarães Lima M. Incidência de internações por eventos adversos a medicamentos em Minas Gerais. *Sci Medica*. 2017;27(1):24936. doi:10.15448/1980-6108.2017.1.24936
17. Pouyanne P. Admissions to hospital caused by adverse drug reactions: cross sectional incidence study. *BMJ*. 2000;320(7241):1036-1036. doi:10.1136/bmj.320.7241.1036
18. Jonville-Béra AP, Giraudeau B, Blanc P, Beau-Salinas F, Autret-Leca E. Frequency of adverse drug reactions in children: A prospective study: *Short report*. *Br J Clin Pharmacol*. 2002;53(2):207-210. doi:10.1046/j.0306-5251.2001.01535.x
19. Haffner S, von Laue N, Wirth S, Thürmann PA. Detecting Adverse Drug Reactions on Paediatric Wards: Intensified Surveillance Versus Computerised Screening of Laboratory Values. *Drug Saf*. 2005;28(5):453-464. doi:10.2165/00002018-200528050-00008
20. Weiss J, Krebs S, Hoffmann C, et al. Survey of Adverse Drug Reactions on a Pediatric Ward: A Strategy for Early and Detailed Detection. *Pediatrics*. 2002;110(2):254-257. doi:10.1542/peds.110.2.254
21. Lamabadusuriya SP, Sathiadas G. Adverse drug reactions in children requiring hospital admission. *Ceylon Med J*. 2003;48(3):86-87.
22. Fattahi F, Pourpak Z, Moin M, et al. Adverse Drug Reactions in Hospitalized Children in a Department of Infectious Diseases. *J Clin Pharmacol*. 2005;45(11):1313-1318. doi:10.1177/0091270005281205
23. Oshikoya KA, Njokanma OF, Chukwura HA, Ojo IO. Adverse drug reactions in Nigerian children. *Paediatr Perinat Drug Ther*. 2007;8(2):81-88. doi:10.1185/146300907X199858
24. Bénard-Laribière A, Miremont-Salamé G, Pérault-Pochat MC, Noize P, Haramburu F, the EMIR Study Group on behalf of the French network of pharmacovigilance centres. Incidence of hospital admissions due to adverse drug reactions in France: the EMIR study. *Fundam Clin Pharmacol*. 2015;29(1):106-111. doi:10.1111/fcp.12088
25. Oshikoya KA, Chukwura H, Njokanma OF, Senbanjo IO, Ojo I. Incidence and cost estimate of treating pediatric adverse drug reactions in Lagos, Nigeria. *Sao Paulo Med J*. 2011;129(3):153-164. doi:10.1590/S1516-31802011000300006
26. Gallagher RM, Bird KA, Mason JR, et al. Adverse drug reactions causing admission to a paediatric hospital: a pilot study: Adverse drug reactions. *J Clin Pharm Ther*. 2011;36(2):194-199. doi:10.1111/j.1365-2710.2010.01194.x
27. Posthumus AAG, Alingh CCW, Zwaan CCM, et al. Adverse drug reaction-related admissions in paediatrics, a prospective single-centre study. *BMJ Open*. 2012;2(4):e000934. doi:10.1136/bmjopen-2012-000934
28. Bellis JR, Kirkham JJ, Nunn AJ, Pirmohamed M. Adverse drug reactions and off-label and unlicensed medicines in children: a prospective cohort study of unplanned admissions to a paediatric hospital: Adverse drug reactions and off-label and unlicensed medicines in children. *Br J Clin Pharmacol*. 2014;77(3):545-553. doi:10.1111/bcp.12222
29. Gallagher RM, Mason JR, Bird KA, et al. Adverse Drug Reactions Causing Admission to a Paediatric Hospital. Choonara I, ed. *PLoS ONE*. 2012;7(12):e50127. doi:10.1371/journal.pone.0050127
30. Langerová P, Vrtal J, Urbánek K. Adverse Drug Reactions Causing Hospital Admissions in Childhood: A Prospective, Observational, Single-Centre Study. *Basic Clin Pharmacol Toxicol*. 2014;115(6):560-564. doi:10.1111/bcpt.12264
31. Russom M, Tesfai D, Elias M, et al. Adverse Drug Reactions among Patients Admitted to Eritrean Hospitals: Prevalence Causes and Risk Factors a prospective analysis of 5848 patient. *Int J Pharmacovigil*. 2017;2(1):1-7. doi:10.15226/2476-2431/2/1/00113

32. Gholami K, Babaie F, Shalviri G, Javadi M, Faghihi T. Pediatric hospital admission due to adverse drug reactions: Report from a tertiary center. *J Res Pharm Pract*. 2015;4(4):212. doi:10.4103/2279-042X.167045
33. Mouton JP, Fortuin-de Smidt MC, Jobanputra N, et al. Serious adverse drug reactions at two children's hospitals in South Africa. *BMC Pediatr*. 2020;20(1):3. doi:10.1186/s12887-019-1892-x
34. Nasso C, Mecchio A, Rottura M, et al. A 7-Years Active Pharmacovigilance Study of Adverse Drug Reactions Causing Children Admission to a Pediatric Emergency Department in Sicily. *Front Pharmacol*. 2020;11:1090. doi:10.3389/fphar.2020.01090
35. Patel PP, Makrani MM, Gandhi AM, Desai MK, Desai CK. An intensive monitoring of adverse drug reactions in pediatric hospitalized patients of a tertiary care hospital. *Int J Basic Clin Pharmacol*. 2021;10(6):704. doi:10.18203/2319-2003.ijbcp20212082
36. Gupta S, Zaki SA, Masavkar S, Shanbag P. Causality, Severity, and Avoidability of Adverse Drug Reactions in Hospitalized Children: A Prospective Cohort Study. *Cureus*. Published online January 4, 2023. doi:10.7759/cureus.33369
37. McDonnell PJ, Jacobs MR, Monsanto HA, Kaiser JM. Hospital admissions resulting from preventable adverse drug reactions. *Ann Pharmacother*. 2002;36(9):1331-1336. doi:10.1345/aph.1A333
38. Duczmal E, Bręborowicz A. Adverse drug reactions as a cause of hospital admission. *Przeglad Pediatryczny*. 2006;36(1):14-18.
39. Impicciatore P, Mohn A, Chiarelli F, Pandolfini C, Bonati M. Adverse drug reactions to off-label drugs on a paediatric ward: An Italian prospective pilot study. *Paediatr Perinat Drug Ther*. 2002;5(1):19-24. doi:10.1185/146300902322125118
40. van der Hooft CS, Dieleman JP, Siemes C, et al. Adverse drug reaction-related hospitalisations: a population-based cohort study: ADR-RELATED HOSPITAL ADMISSIONS. *Pharmacoepidemiol Drug Saf*. 2008;17(4):365-371. doi:10.1002/pds.1565
41. Le J, Nguyen T, Law AV, Hodding J. Adverse Drug Reactions Among Children Over a 10-Year Period. *Pediatrics*. 2006;118(2):555-562. doi:10.1542/peds.2005-2429
42. Speranza N, Lucas L, Telechea H, Santurio A, Giachetto G, Nanni L. Adverse Drugs Reactions in Hospitalized Children: A Public Health Problem. *Drug Saf*. 2008;31(10):885. doi:10.2165/00002018-200831100-00130
43. Moschini M, Lombardi N, Pugi A, et al. Monitoring program of adverse drug reactions in a pediatric emergency department. *Drug Saf*. 2013;36(9):913. doi:10.1007/s40264-013-0087-x
44. Mendoza Otero F, Iniesta Navalon C, García Molina O, Fernandez De Palencia Espinosa M, Galindo Rueda M, De La Rubia Nieto A. Adverse drug reactions causing admission over 11 years in a paediatric hospital. *Eur J Hosp Pharm*. 2015;22(Suppl 1):A184.3-A185. doi:10.1136/ejhpharm-2015-000639.444
45. Lombardi N, Crescioli G, Bettiol A, et al. Characterization of serious adverse drug reactions as cause of emergency department visit in children: a 5-years active pharmacovigilance study. *BMC Pharmacol Toxicol*. 2018;19(1):16. doi:10.1186/s40360-018-0207-4
46. Morales-Ríos O, Cicero-Oneto C, García-Ruiz C, et al. Descriptive study of adverse drug reactions in a tertiary care pediatric hospital in México from 2014 to 2017. Yang JM, ed. *PLOS ONE*. 2020;15(3):e0230576. doi:10.1371/journal.pone.0230576
47. Strand LM, Morley PC, Cipolle RJ, Ramsey R, Lamsam GD. Drug-related problems: their structure and function. *DICP Ann Pharmacother*. 1990;24(11):1093-1097. doi:10.1177/106002809002401114
48. Nebeker JR, Barach P, Samore MH. Clarifying adverse drug events: a clinician's guide to terminology, documentation, and reporting. *Ann Intern Med*. 2004;140(10):795-801. doi:10.7326/0003-4819-140-10-200405180-00009
49. Hepler C, Strand L. Opportunities and responsibilities in pharmaceutical care. *Am J Hosp Pharm*. 1990;47(3):533-543.
50. Möller H, Aly AF. Definitionen zu Pharmakovigilanz und Arzneimitteltherapiesicherheit (AMTS). *Z Für Evidenz Fortbild Qual Im Gesundheitswesen*. 2012;106(10):709-711. doi:10.1016/j.zefq.2012.10.023
51. Bates DW, Cullen DJ, Laird N, et al. Incidence of adverse drug events and potential adverse drug events. Implications for prevention. ADE Prevention Study Group. *JAMA*. 1995;274(1):29-34.
52. Elixhauser A, Owens P. Adverse Drug Events in U.S. Hospitals, 2004. *HCUP Stat Brief 29*. 2007;Agency for Healthcare Research and Quality, Rockville, MD. Accessed July 1, 2022.
53. Edwards IR, Aronson JK. Adverse drug reactions: definitions, diagnosis, and management. *The Lancet*. 2000;356(9237):1255-1259. doi:10.1016/S0140-6736(00)02799-9
54. Krähenbühl-Melcher A, Schlienger R, Lampert M, Haschke M, Drewe J, Krähenbühl S. Drug-Related Problems in Hospitals: A Review of the Recent Literature. *Drug Saf*. 2007;30(5):379-407. doi:10.2165/00002018-200730050-00003
55. Aronson JK, Ferner RE. Clarification of Terminology in Drug Safety: *Drug Saf*. 2005;28(10):851-870. doi:10.2165/00002018-200528100-00003
56. Naranjo CA, Busto U, Sellers EM, et al. A method for estimating the probability of adverse drug reactions. *Clin Pharmacol Ther*. 1981;30(2):239-245. doi:10.1038/clpt.1981.154
57. World Health Organization. International drug monitoring. The role of the hospital. *Tech Rep Ser No 425*. 1969;425:1-24.
58. Dartnell JG, Anderson RP, Chohan V, et al. Hospitalisation for adverse events related to drug therapy: incidence, avoidability and costs. *Med J Aust*. 1996;164(11):659-662. doi:10.5694/j.1326-5377.1996.tb122235.x
59. Bégaud B, Evreux JC, Jouglard J, Lagier G. [Imputation of the unexpected or toxic effects of drugs. Actualization of the method used in France]. *Therapie*. 1985;40(2):111-118.
60. Evans RS, Pestotnik SL, Classen DC, et al. Development of a computerized adverse drug event monitor. *Proc Symp Comput Appl Med Care*. Published online 1991:23-27.
61. Jones JK. Adverse drug reactions in the community health setting: approaches to recognizing, counseling, and reporting. *Fam Community Health*. 1982;5(2):58-67. doi:10.1097/00003727-198208000-00009
62. Gallagher RM, Kirkham JJ, Mason JR, et al. Development and Inter-Rater Reliability of the Liverpool Adverse Drug Reaction Causality Assessment Tool. Timmer A, ed. *PLoS ONE*. 2011;6(12):e28096. doi:10.1371/journal.pone.0028096
63. Karch FE, Lasagna L. Toward the operational identification of adverse drug reactions. *Clin Pharmacol Ther*. 1977;21(3):247-254. doi:10.1002/cpt1977213247
64. Schumock G, Thornton J. Focusing on the preventability of adverse drug reactions. *Hosp Pharm*. 1992;27:538.
65. Hallas J, Harvald B, Gram L, Grodum E, Brosen K. Drug related hospital admissions: the role of definitions and intensity of data collection, and the possibility of prevention. *J Intern Med*. 1990;228:83-90.
66. Global Burden of Disease Collaborative Network. Global Burden of Disease Study 2019 (GBD 2019) Socio-Demographic Index (SDI) 1950–2019. Published online 2020. https://doi.org/10.6069/D8QB-JK35
