# Supplementary material for: The epidemiology of drug-related hospital admissions in paediatrics – a systematic review
Source: Arch Public Health. 2024 Jun 4;82:81. doi: 10.1186/s13690-024-01295-4 (PMC11149243; doi:10.1186/s13690-024-01295-4)
Supplement: Supplementary file 6 — Additional file 6. Critical appraisal results details. [file 13690_2024_1295_MOESM6_ESM.pdf]

Details of the critical appraisal

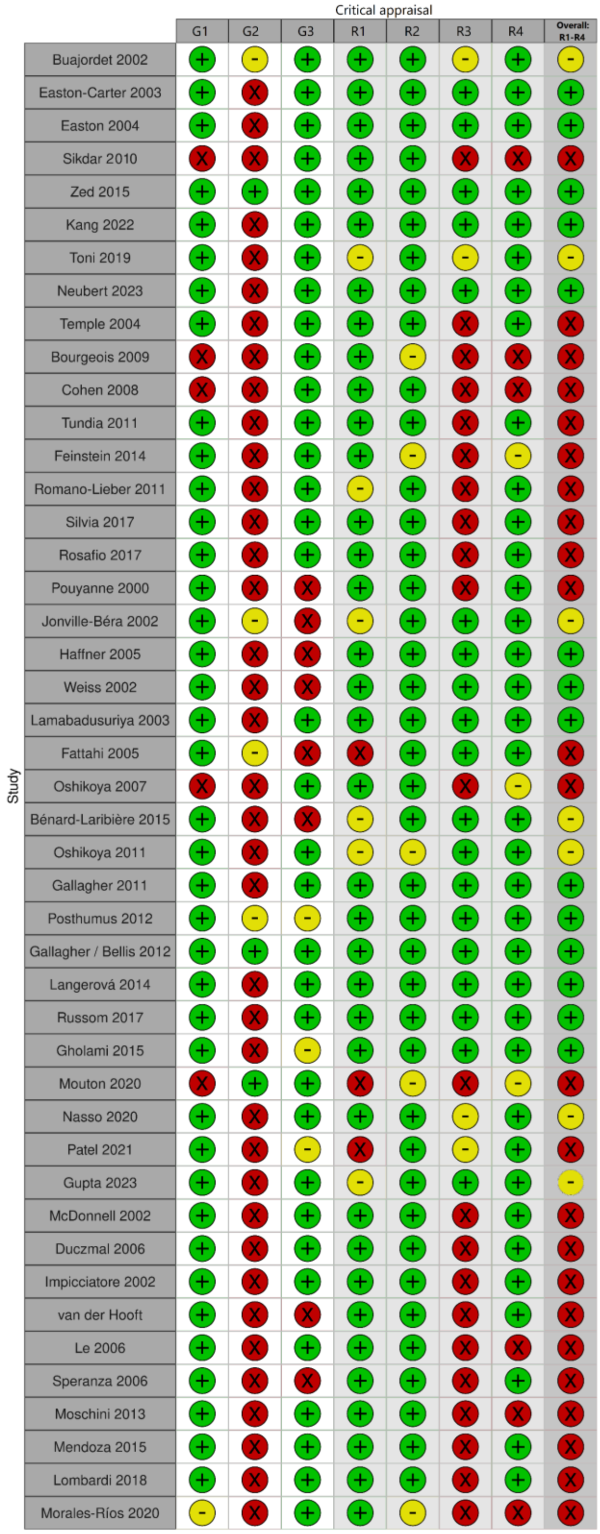

Figure 1 Traffic light plot – overview

Critical appraisal domains:

G1: General applicability
G2: General reporting issues
G3: General issues in precision

R1: Risk of selection bias
R2: Risk of information bias due to attrition
R3: Risk of information bias due to issues with methods and measurements
R4: Risk of information bias due to issues with data analysis
Overall: R1-4: Overall risk of bias appraisal.

Judgement: High risk; Some concerns; Low risk

Description of the results of the critical appraisal

In all studies, the research question was principally appropriate to provide data on drug-related hospitalisations, especially when 'drug-related hospitalisation' was one of the primary study objectives.

G1) General applicability: Limitations in the general applicability were observed especially in studies[23,33] that combined two different study approaches without taking this into account (e.g. the combination of different settings or both a retrospective and a prospective cohort). Further, in some studies[4,10,11], the frequency could only be derived indirectly (e.g. via the results of primary objectives) or the outcome was rarely informative.

G2) General reporting issues: In our evaluation we encountered serious reporting issues: All studies with 'routine monitoring'[9–17,37–46] lacked information on the characteristics of the study design, especially the description of the study population. This problem was found also in 20[2–4,6–8,17,19–21,23–26,30–32,34–36] of the remaining 27 studies.

G3) General issues in precision: In eight studies[17–20,22,24,40,42], the study authors had chosen a small sample size despite the predictable risk for

imprecision. These issues with precision were mostly confirmed by wide confidence intervals in the frequency evaluation.

R1) Risk of selection bias: Most studies included all patients within their setting during the observation period and did not select patients. However, there was one study with an indecisive inclusion process leading to a high risk of selection bias.[35] Additionally, there could be a risk of selection bias in some studies[22,33,(7,14,18,24,25,36)], e.g. with very short study periods due to seasonal effects or if repeated admissions or short stays were excluded.

R2) Risk of information bias due to attrition: Data of all patients were usually evaluated; thus, we hardly observed a risk of attrition bias (R2). Only a few studies[(10,13,25,33,46)] were inconclusive or if patients were excluded, e.g. due to missing documentation, this might have affected an above-average number of patients with problems.

R3) Risk of information bias due to issues with methods and measurements: All studies using 'routine monitoring' were considered at high risk of under-recording for the outcome 'drug-related hospitalisation. In these studies, the method and implementation of the measurement led to a high risk of information bias. Among the 27 studies with 'intensive monitoring', this problem occurred in four studies[4,17,23,33].

R4) Risk of information bias due to issues with data analysis: We identified a risk of bias in the data analysis, i.e. when studies[4,10,11,41,43,46] did not clearly report the number of cases and the size of the reference population.

Additional) Risk of confounding: In addition to descriptive assessments, some studies[25,28,30,33,37] also conducted analytical investigations on factors influencing the occurrence of drug-related hospitalisations. For these outcomes, we extended the critical appraisal: the selected subgroups were correctly identified, but potential influencing factors (such as age, gender, number of comedication, clinical condition of the patients) were not always addressed. So, there could be a high risk of confounding. Only Gallagher et al[28] accounted more comprehensively for variables in their regression analysis, leading to only some concerns.

Reference list

1. Buajordet I, Wesenberg F, Brørs O, Langslet A. Adverse drug events in children during hospitalization and after discharge in a Norwegian University Hospital. *Acta Paediatr*. 2002;91(1):88-94. doi:10.1080/080352502753458021
2. Easton-Carter K, Chapman C, Brien J. Emergency department attendances associated with drug-related problems in paediatrics. *J Paediatr Child Health*. 2003;39(2):124-129. doi:10.1046/j.1440-1754.2003.00103.x
3. Easton KL, Chapman CB, Brien J anne E. Frequency and characteristics of hospital admissions associated with drug-related problems in paediatrics. *Br J Clin Pharmacol*. 2004;57(5):611-615. doi:10.1111/j.1365-2125.2003.02052.x
4. Sikdar KC, Alaghehbandan R, MacDonald D, Barrett B, Collins KD, Gadag V. Adverse drug events among children presenting to a hospital emergency department in Newfoundland and Labrador, Canada: ADVERSE DRUG EVENTS AMONG CHILDREN. *Pharmacoepidemiol Drug Saf*. 2010;19(2):132-140. doi:10.1002/pds.1900
5. Zed PJ, Black KJL, Fitzpatrick EA, et al. Medication-Related Emergency Department Visits in Pediatrics: a Prospective Observational Study. *Pediatrics*. 2015;135(3):435-443. doi:10.1542/peds.2014-1827
6. Kang MG, Lee JY, Woo SI, et al. Adverse drug events leading to emergency department visits: A multicenter observational study in Korea. Mogi M, ed. *PLOS ONE*. 2022;17(9):e0272743. doi:10.1371/journal.pone.0272743
7. Toni I, Wimmer S, Trollmann R, Rascher W, Neubert A. Drug-related hospital admissions in paediatrics-what is preventable? *Arch Dis Child*. 2019;104(6). doi:10.1136/archdischild-2019-esdppp.1
8. Neubert A, Toni I, König J, et al. A complex intervention to prevent medication-related hospital admissions—results of the stepped-wedge cluster randomized trial KiDSafe in pediatrics. *Dtsch Ärztebl Int*. Published online June 23, 2023. doi:10.3238/arztebl.m2023.0123
9. Temple ME, Robinson RF, Miller JC, Hayes JR, Nahata MC. Frequency and Preventability of Adverse Drug Reactions in Paediatric Patients: *Drug Saf*. 2004;27(11):819-829. doi:10.2165/00002018-200427110-00005
10. Bourgeois FT, Mandl KD, Valim C, Shannon MW. Pediatric Adverse Drug Events in the Outpatient Setting: An 11-Year National Analysis. *Pediatrics*. 2009;124(4):e744-e750. doi:10.1542/peds.2008-3505
11. Cohen AL, Budnitz DS, Weidenbach KN, et al. National Surveillance of Emergency Department Visits for Outpatient Adverse Drug Events in Children and Adolescents. *J Pediatr*. 2008;152(3):416-421.e2. doi:10.1016/j.jpeds.2007.07.041
12. Tundia NL, Heaton PC, Kelton CML. The national burden of E-code-identified adverse drug events among hospitalized children using a national discharge database: NATIONAL BURDEN OF PEDIATRIC ADVERSE DRUG EVENTS. *Pharmacoepidemiol Drug Saf*. 2011;20(8):866-878. doi:10.1002/pds.2150
13. Feinstein JA, Feudtner C, Kempe A. Adverse Drug Event-Related Emergency Department Visits Associated With Complex Chronic Conditions. *Pediatrics*. 2014;133(6):e1575-e1585. doi:10.1542/peds.2013-3060
14. Romano-Lieber NS, Ribeiro E. Adverse drug events leading children to emergency department, São Paulo, Brazil. *Pharmacoepidemiol Drug Saf*. 2011;20((Romano-Lieber N.S.) School of Public Health, University of São Paulo, São Paulo, SP, Brazil):S320-S321. doi:10.1002/pds.2206
15. Silva YDOM, Guimarães Lima M. Incidência de internações por eventos adversos a medicamentos em Minas Gerais. *Sci Medica*. 2017;27(1):24936. doi:10.15448/1980-6108.2017.1.24936
16. Rosafio C, Paioli S, Del Giovane C, et al. Medication-related visits in a pediatric emergency department: an 8-years retrospective analysis. *Ital J Pediatr*. 2017;43(1):55. doi:10.1186/s13052-017-0375-7
17. Pouyanne P. Admissions to hospital caused by adverse drug reactions: cross sectional incidence study. *BMJ*. 2000;320(7241):1036-1036. doi:10.1136/bmj.320.7241.1036
18. Jonville-Béra AP, Giraudeau B, Blanc P, Beau-Salinas F, Autret-Leca E. Frequency of adverse drug reactions in children: A prospective study: *Short report*. *Br J Clin Pharmacol*. 2002;53(2):207-210. doi:10.1046/j.0306-5251.2001.01535.x
19. Haffner S, von Laue N, Wirth S, Thürmann PA. Detecting Adverse Drug Reactions on Paediatric Wards: Intensified Surveillance Versus Computerised Screening of Laboratory Values. *Drug Saf*. 2005;28(5):453-464. doi:10.2165/00002018-200528050-00008
20. Weiss J, Krebs S, Hoffmann C, et al. Survey of Adverse Drug Reactions on a Pediatric Ward: A Strategy for Early and Detailed Detection. *Pediatrics*. 2002;110(2):254-257. doi:10.1542/peds.110.2.254
21. Lamabadusuriya SP, Sathiadas G. Adverse drug reactions in children requiring hospital admission. *Ceylon Med J*. 2003;48(3):86-87.
22. Fattahi F, Pourpak Z, Moin M, et al. Adverse Drug Reactions in Hospitalized Children in a Department of Infectious Diseases. *J Clin Pharmacol*. 2005;45(11):1313-1318. doi:10.1177/0091270005281205
23. Oshikoya KA, Njokanma OF, Chukwura HA, Ojo IO. Adverse drug reactions in Nigerian children. *Paediatr Perinat Drug Ther*. 2007;8(2):81-88. doi:10.1185/146300907X199858
24. Bénard-Laribière A, Miremont-Salamé G, Pérault-Pochat MC, Noize P, Haramburu F, the EMIR Study Group on behalf of the French network of pharmacovigilance centres. Incidence of hospital admissions due to adverse drug reactions in France: the EMIR study. *Fundam Clin Pharmacol*. 2015;29(1):106-111. doi:10.1111/fcp.12088

25. Oshikoya KA, Chukwura H, Njokanma OF, Senbanjo IO, Ojo I. Incidence and cost estimate of treating pediatric adverse drug reactions in Lagos, Nigeria. *Sao Paulo Med J*. 2011;129(3):153-164. doi:10.1590/S1516-31802011000300006

26. Gallagher RM, Bird KA, Mason JR, et al. Adverse drug reactions causing admission to a paediatric hospital: a pilot study: Adverse drug reactions. *J Clin Pharm Ther*. 2011;36(2):194-199. doi:10.1111/j.1365-2710.2010.01194.x

27. Posthumus AAG, Alingh CCW, Zwaan CCM, et al. Adverse drug reaction-related admissions in paediatrics, a prospective single-centre study. *BMJ Open*. 2012;2(4):e000934. doi:10.1136/bmjopen-2012-000934

28. Gallagher RM, Mason JR, Bird KA, et al. Adverse Drug Reactions Causing Admission to a Paediatric Hospital. Choonara I, ed. *PLoS ONE*. 2012;7(12):e50127. doi:10.1371/journal.pone.0050127

29. Bellis JR, Kirkham JJ, Nunn AJ, Pirmohamed M. Adverse drug reactions and off-label and unlicensed medicines in children: a prospective cohort study of unplanned admissions to a paediatric hospital: Adverse drug reactions and off-label and unlicensed medicines in children. *Br J Clin Pharmacol*. 2014;77(3):545-553. doi:10.1111/bcp.12222

30. Langerová P, Vrtal J, Urbánek K. Adverse Drug Reactions Causing Hospital Admissions in Childhood: A Prospective, Observational, Single-Centre Study. *Basic Clin Pharmacol Toxicol*. 2014;115(6):560-564. doi:10.1111/bcpt.12264

31. Russom M, Tesfai D, Elias M, et al. Adverse Drug Reactions among Patients Admitted to Eritrean Hospitals: Prevalence Causes and Risk Factors a prospective analysis of 5848 patient. *Int J Pharmacovigil*. 2017;2(1):1-7. doi:10.15226/2476-2431/2/1/00113

32. Gholami K, Babaie F, Shalviri G, Javadi M, Faghihi T. Pediatric hospital admission due to adverse drug reactions: Report from a tertiary center. *J Res Pharm Pract*. 2015;4(4):212. doi:10.4103/2279-042X.167045

33. Mouton JP, Fortuin-de Smidt MC, Jobanputra N, et al. Serious adverse drug reactions at two children's hospitals in South Africa. *BMC Pediatr*. 2020;20(1):3. doi:10.1186/s12887-019-1892-x

34. Nasso C, Mecchio A, Rottura M, et al. A 7-Years Active Pharmacovigilance Study of Adverse Drug Reactions Causing Children Admission to a Pediatric Emergency Department in Sicily. *Front Pharmacol*. 2020;11:1090. doi:10.3389/fphar.2020.01090

35. Patel PP, Makrani MM, Gandhi AM, Desai MK, Desai CK. An intensive monitoring of adverse drug reactions in pediatric hospitalized patients of a tertiary care hospital. *Int J Basic Clin Pharmacol*. 2021;10(6):704. doi:10.18203/2319-2003.ijbcp20212082

36. Gupta S, Zaki SA, Masavkar S, Shanbag P. Causality, Severity, and Avoidability of Adverse Drug Reactions in Hospitalized Children: A Prospective Cohort Study. *Cureus*. Published online January 4, 2023. doi:10.7759/cureus.33369

37. McDonnell PJ, Jacobs MR, Monsanto HA, Kaiser JM. Hospital admissions resulting from preventable adverse drug reactions. *Ann Pharmacother*. 2002;36(9):1331-1336. doi:10.1345/aph.1A333

38. Duczmal E, Bręborowicz A. Adverse drug reactions as a cause of hospital admission. *Przeglad Pediatryczny*. 2006;36(1):14-18.

39. Impicciatore P, Mohn A, Chiarelli F, Pandolfini C, Bonati M. Adverse drug reactions to off-label drugs on a paediatric ward: An Italian prospective pilot study. *Paediatr Perinat Drug Ther*. 2002;5(1):19-24. doi:10.1185/146300902322125118

40. van der Hooft CS, Dieleman JP, Siemes C, et al. Adverse drug reaction-related hospitalisations: a population-based cohort study: ADR-RELATED HOSPITAL ADMISSIONS. *Pharmacoepidemiol Drug Saf*. 2008;17(4):365-371. doi:10.1002/pds.1565

41. Le J, Nguyen T, Law AV, Hodding J. Adverse Drug Reactions Among Children Over a 10-Year Period. *Pediatrics*. 2006;118(2):555-562. doi:10.1542/peds.2005-2429

42. Speranza N, Lucas L, Telechea H, Santurio A, Giachetto G, Nanni L. Adverse Drugs Reactions in Hospitalized Children: A Public Health Problem. *Drug Saf*. 2008;31(10):885. doi:10.2165/00002018-200831100-00130

43. Moschini M, Lombardi N, Pugi A, et al. Monitoring program of adverse drug reactions in a pediatric emergency department. *Drug Saf*. 2013;36(9):913. doi:10.1007/s40264-013-0087-x

44. Mendoza Otero F, Iniesta Navalon C, García Molina O, Fernandez De Palencia Espinosa M, Galindo Rueda M, De La Rubia Nieto A. Adverse drug reactions causing admission over 11 years in a paediatric hospital. *Eur J Hosp Pharm*. 2015;22(Suppl 1):A184.3-A185. doi:10.1136/ejhpharm-2015-000639.444

45. Lombardi N, Crescioli G, Bettiol A, et al. Characterization of serious adverse drug reactions as cause of emergency department visit in children: a 5-years active pharmacovigilance study. *BMC Pharmacol Toxicol*. 2018;19(1):16. doi:10.1186/s40360-018-0207-4

46. Morales-Ríos O, Cicero-Oneto C, García-Ruiz C, et al. Descriptive study of adverse drug reactions in a tertiary care pediatric hospital in México from 2014 to 2017. Yang JM, ed. *PLOS ONE*. 2020;15(3):e0230576. doi:10.1371/journal.pone.0230576
