# Supplementary material for: The epidemiology of drug-related hospital admissions in paediatrics – a systematic review
Source: Arch Public Health. 2024 Jun 4;82:81. doi: 10.1186/s13690-024-01295-4 (PMC11149243; doi:10.1186/s13690-024-01295-4)
Supplement: Supplementary file 7 — Additional file 7. Drugs implicated and clinical manifestations. [file 13690_2024_1295_MOESM7_ESM.pdf]

Available data on the nature of drug-related hospitalisations

		Most common drugs (or groups) involved in drug-related hospital admissions	Most common clinical manifestations
1a.	Buajordet 2002[1]	Cytotoxic drugs, vaccination	Haematological reactions after treatment with cytotoxic drugs (22 / 53); complications to vaccination (9 / 53)
	Easton 2004[2]	Beclomethasone; flucloxacillin; insulin-isophane; paracetamol; sodium valproate; insulin-neutral; budesonide; fluticasone; diazepam; theophylline; sodium cromoglycate; dothiepin; oxazepam; and triple antigen vaccine	NR
1b.	Jonville-Béra 2002[4]	Antiepileptic drug, analgesic drug, acetyl salicylic acid, methadone (total: 4 drugs involved in 4 ADRs)	1x convulsion, 1x myoclonia, 1x melaena, 1x neonatal withdrawal syndrome (total: 4 ADRs)
	Lamabadusuriya 2003[5]	Ampicillin and amoxicillin (20.6%, n=13), cotrimoxazole (11.1%, n=7), metoclopramide (11.1%, n=7), amoxicillin/clavulanic acid (3.2%, n=2), aspirin (3.2%, n=2), penicillin (9.5%, n=6), flucloxacillin (1.6%, n=1), erythromycin (4.8%, n=3), nalidixic acid (9.5%, n=6), diclofenac sodium suppository (4.8%, n=3), sodium valproate (3.2%, n=2) nalidixic acid (9.5%, n=6), diclofenac sodium suppository (4.8%, n=3), sodium valproate (3.2%, n=2), lamotrigine (1.6%, n=1), carbamazepine(1.6%, n=1), JE vaccine (9.5%, n=6), DPR vaccine (4.8%, n=3)	The nervous system was involved in 17 (27%), respiratory system in 6 (10%), gastrointestinal system in 8 (12%), and skin in 33 (52%). There were no renal or cardiovascular adverse reactions. ... When mechanism of action was examined 42 (67%) were pharmacological, 14 (22%) allergic manifestations, and 7 (11%) idiosyncratic.
	Oshikoya 2011[6]	Co-trimoxazole (6x), ampicillin (4x), sulfadoxine/pyrimethamine (3x), albendazole, amodiaquine / artesunate, cefixime, loperamide, phenobarbitone, prednisolone (total: 19 drugs involved in 12 ADRs)	Erythema multiforme (5x), Stevens-Johnson syndrome (2x), macular and morbilliform rash (2x), ileus (1x), dystonia + hypothermia (1x), electrolyte disturbance + hyperglycaemia(1x) (total 12 ADRs)
	Gallagher 2011[7]	Anti-neoplastic drugs, immunosuppressants, antibiotics and analgesics	Neutropenia, vomiting, diarrhoea, rash, immunosuppression, thrombocytopenia, irritability, altered conscious level, anaemia, rash, constipation, haematemesis, impaired renal function, anaphylaxis, fever, infection (cellulitis)
	Posthumus 2012[8]	Anti-neoplastic and immunomodulating agents, vaccinations, anti-infectives for systemic use, immunomodulating agents, drugs for the nervous system	Neutropenic fever, fever (+refusal of food or tachycardia + abdominal pain or vomiting), vomiting, (retinal) haemorrhage, ataxia and diarrhoea, constipation, thrombopenia / petechiae, anaemia, drowsiness, dyspnoea, leucopoenia, pancreatitis, sickness, syncope, urticaria
	Gallagher 2012[9] / Bellis 2014[10]	Cytotoxic drugs, corticosteroids, non-steroidal anti-inflammatory drugs (NSAIDs), vaccines, immunosuppressants	Oncology related including neutropenia, thrombocytopenia and anaemia; immunosuppression, occurring in both oncology and non-oncology patients; post-operative bleeding, linked to peri-operative corticosteroid administration and/or NSAIDs; vomiting, diarrhoea; rash; constipation; hypoglycaemia in diabetic patients treated with regular insulin; respiratory depression following treatment for status epilepticus
	Langerová 2014[11]	Anticancer chemotherapy 35%, antibiotics 18%, antipyretic drugs 6%, hormonal contraception 6%, vaccines 9%, immunosuppressants 9%, glucocorticoids 5%, biologic therapy 3%, others 9%	Febrile neutropenia and mucositis (anticancer chemotherapy); vomiting and allergic reactions (exanthema) (antibiotics)
	Gholami 2015[12]	Anti-infective agents (e.g. cefixime) (37%), central nervous system agents (e.g. phenobarbital) (22.2%) serums, toxoids, and vaccines (18.5%), antineoplastic agents (11.2%), miscellaneous therapeutic agents (7.4%) antihistamine drugs (3.7%)	Maculopapular rashes (12.5%), diarrhoea (12.5%), and vomiting (10.4%)
	Mouton 2020[13]	Remifentanil, fentanyl, benzathine benzylpenicillin, furosemide + spironolactone, phenoxymethylpenicillin, carvedilol, prednisone, metoclopramide, ceftriaxone, zidovudine, co-trimoxazole, isoniazid, amoxicillin, ferrous gluconate, amikacin + paracetamol, clozapine, prednisone, metoclopramide, amitriptyline, enalapril, tacrolimus, mycophenolic acid, erythropoietin	Neonatal respiratory depression, cardio-respiratory arrest, respiratory arrest, cardiogenic shock, urticaria, cardiac failure congestive, lower respiratory tract infection, dystonia, urticaria, agranulocytosis, convulsion, thrombocytopenia, constipation, metabolic acidosis, delirium, upper respiratory tract infection, dystonia, somnolence, hyperkalaemia, anaemia macrocytic
	Nasso 2020[14]	Vaccines (51.6%) antibacterials for systemic use (28.6%) anti-inflammatory and antirheumatic products (12.1%) analgesics (5.5%) drugs for obstructive airway diseases (2.2%) antiepileptics (4.4%) corticosteroids for systemic use (2.2%) antitussives (1.1%) antihistamines for systemic use (1.1%) psycholeptics (1.1%) agents acting on the renin-angiotensin system (1.1%) antineoplastic and immunomodulating agents (1.1%)	NR
1c.	Duczmal 2006[15 a,b]	Semisynthetic penicillin, cephalosporin and non-steroid anti-inflammatory drugs	Allergic reaction, intolerance of non-steroid anti-inflammatory drugs, photoallergic reactions, Hoigné syndrome
	Le 2006[16]	Anticonvulsants (valproic acid, phenobarbital, and phenytoin)	NR
	Mendoza 2015[17 b]	Antineoplastic drugs (65.0%), drugs active on the central nervous system (8.6%) and anti-infective agents (5.8%)	NR
	Morales-Ríos 2020[18]	NR	Febrile neutropenia, sepsis, pancytopenia, septic shock, mucositis

1a. Available data on the nature of drug-related hospitalisations – Studies with a broader concept of 'adverse drug events', 'with intensive monitoring' (yellow background)
1b. Available data on the nature of drug-related hospitalisations – Studies with a narrower concept of 'adverse drug reactions', 'with intensive monitoring' (green background
1c. Available data on the nature of drug-related hospitalisations – Studies with a narrower concept of 'adverse drug reactions', 'based on routine monitoring' (blue background)

[a] study identified via citation search; [b] just abstract of the study available
Abbreviations: NR Not reported (not reported at all or not reported separately for drug-related hospitalisations), *ADR* Adverse drug reaction

Bibliography

1. Buajordet I, Wesenberg F, Brørs O, Langslet A. Adverse drug events in children during hospitalization and after discharge in a Norwegian University Hospital. *Acta Paediatr*. 2002;91(1):88-94. doi:10.1080/080352502753458021
2. Easton KL, Chapman CB, Brien J anne E. Frequency and characteristics of hospital admissions associated with drug-related problems in paediatrics. *Br J Clin Pharmacol*. 2004;57(5):611-615. doi:10.1111/j.1365-2125.2003.02052.x
3. Toni I, Wimmer S, Trollmann R, Rascher W, Neubert A. Drug-related hospital admissions in paediatrics-what is preventable? *Arch Dis Child*. 2019;104(6). doi:10.1136/archdischild-2019-esdppp.1
4. Jonville-Béra AP, Giraudeau B, Blanc P, Beau-Salinas F, Autret-Leca E. Frequency of adverse drug reactions in children: A prospective study: *Short report*. *Br J Clin Pharmacol*. 2002;53(2):207-210. doi:10.1046/j.0306-5251.2001.01535.x
5. Lamabadusuriya SP, Sathiadas G. Adverse drug reactions in children requiring hospital admission. *Ceylon Med J*. 2003;48(3):86-87.
6. Oshikoya KA, Chukwura H, Njokanma OF, Senbanjo IO, Ojo I. Incidence and cost estimate of treating pediatric adverse drug reactions in Lagos, Nigeria. *Sao Paulo Med J*. 2011;129(3):153-164. doi:10.1590/S1516-31802011000300006
7. Gallagher RM, Bird KA, Mason JR, et al. Adverse drug reactions causing admission to a paediatric hospital: a pilot study: Adverse drug reactions. *J Clin Pharm Ther*. 2011;36(2):194-199. doi:10.1111/j.1365-2710.2010.01194.x
8. Posthumus AAG, Alingh CCW, Zwaan CCM, et al. Adverse drug reaction-related admissions in paediatrics, a prospective single-centre study. *BMJ Open*. 2012;2(4):e000934. doi:10.1136/bmjopen-2012-000934
9. Gallagher RM, Mason JR, Bird KA, et al. Adverse Drug Reactions Causing Admission to a Paediatric Hospital. Choonara I, ed. *PLoS ONE*. 2012;7(12):e50127. doi:10.1371/journal.pone.0050127
10. Bellis JR, Kirkham JJ, Nunn AJ, Pirmohamed M. Adverse drug reactions and off-label and unlicensed medicines in children: a prospective cohort study of unplanned admissions to a paediatric hospital: Adverse drug reactions and off-label and unlicensed medicines in children. *Br J Clin Pharmacol*. 2014;77(3):545-553. doi:10.1111/bcp.12222
11. Langerová P, Vrtal J, Urbánek K. Adverse Drug Reactions Causing Hospital Admissions in Childhood: A Prospective, Observational, Single-Centre Study. *Basic Clin Pharmacol Toxicol*. 2014;115(6):560-564. doi:10.1111/bcpt.12264
12. Gholami K, Babaie F, Shalviri G, Javadi M, Faghihi T. Pediatric hospital admission due to adverse drug reactions: Report from a tertiary center. *J Res Pharm Pract*. 2015;4(4):212. doi:10.4103/2279-042X.167045
13. Mouton JP, Fortuin-de Smidt MC, Jobanputra N, et al. Serious adverse drug reactions at two children's hospitals in South Africa. *BMC Pediatr*. 2020;20(1):3. doi:10.1186/s12887-019-1892-x
14. Nasso C, Mecchio A, Rottura M, et al. A 7-Years Active Pharmacovigilance Study of Adverse Drug Reactions Causing Children Admission to a Pediatric Emergency Department in Sicily. *Front Pharmacol*. 2020;11:1090. doi:10.3389/fphar.2020.01090
15. Duczmal E, Bręborowicz A. Adverse drug reactions as a cause of hospital admission. *Przeglad Pediatryczny*. 2006;36(1):14-18.
16. Le J, Nguyen T, Law AV, Hodding J. Adverse Drug Reactions Among Children Over a 10-Year Period. *Pediatrics*. 2006;118(2):555-562. doi:10.1542/peds.2005-2429
17. Mendoza Otero F, Iniesta Navalon C, García Molina O, Fernandez De Palencia Espinosa M, Galindo Rueda M, De La Rubia Nieto A. Adverse drug reactions causing admission over 11 years in a paediatric hospital. *Eur J Hosp Pharm*. 2015;22(Suppl 1):A184.3-A185. doi:10.1136/ejhpharm-2015-000639.444
18. Morales-Ríos O, Cicero-Oneto C, García-Ruiz C, et al. Descriptive study of adverse drug reactions in a tertiary care pediatric hospital in México from 2014 to 2017. Yang JM, ed. *PLOS ONE*. 2020;15(3):e0230576. doi:10.1371/journal.pone.0230576
