# Supplementary material for: The epidemiology of drug-related hospital admissions in paediatrics – a systematic review
Source: Arch Public Health. 2024 Jun 4;82:81. doi: 10.1186/s13690-024-01295-4 (PMC11149243; doi:10.1186/s13690-024-01295-4)
Supplement: Supplementary file 8 — Additional file 8. Details on influencing factors. [file 13690_2024_1295_MOESM8_ESM.pdf]

Details on important influencing factors

Informative subgroup analyses or regression analyses were rarely conducted[1–5]. These found an increased drug-related hospital admission risk for oncology patients. Langerová et al[4] and Gallagher et al[2] were able to show this using regression analyses following their subgroup analyses (Langerová: odds ratio = 9.8, 95% CI (5.77; 16.65), p-value < 0.0001, when controlled for age and gender; Gallagher: odds ratio = 29.7, 95% CI (17.4; 50.9), p-value < 0.0001, when controlled for age, gender and number of medicines). However, their regression analyses did not reveal a significant age effect. According to the subgroup analysis by Mouton et al[5], this could be due to a non-linear influence of age with a higher risk in preterm/neonates and in middle childhood and early adolescence. Regression analyses indicated that females might be affected more often. This was also shown by subgroup analyses[1,2,4,5]. However, this is not entirely clear[3,6] and other influences could not be ruled out (e.g. medical conditions, preference). Gallagher et al[2] also identified the number of medicines as an additional risk factor in his regression analysis. The second evaluation of this study by Bellis et al[7] additionally investigated whether the unlicensed or off-label use of drugs is a risk factor for ADR-related admissions. Although these drugs were more frequently involved than authorised drugs, the authors concluded from regression analyses that their high proportion among oncology medicines was the contributing factor. In other studies, the impact of off-label use was not systematically investigated. Only two studies[8,9] found a high proportion of off-label use among drug-related hospitalisations, but neither with adequate comparative data nor with analyses. Beyond that, data on other patient level factors were not available (such as clinical condition, complex chronic diseases, severity of the diseases, etc.). Additionally, other factors such as seasonal influences[6], socioeconomic influences, or differing organisational processes in medication still need to be further investigated.

Reference list

1. McDonnell PJ, Jacobs MR, Monsanto HA, Kaiser JM. Hospital admissions resulting from preventable adverse drug reactions. *Ann Pharmacother*. 2002;36(9):1331-1336. doi:10.1345/aph.1A333

2. Gallagher RM, Mason JR, Bird KA, et al. Adverse Drug Reactions Causing Admission to a Paediatric Hospital. Choonara I, ed. *PLoS ONE*. 2012;7(12):e50127. doi:10.1371/journal.pone.0050127

3. Oshikoya KA, Chukwura H, Njokanma OF, Senbanjo IO, Ojo I. Incidence and cost estimate of treating pediatric adverse drug reactions in Lagos, Nigeria. *Sao Paulo Med J*. 2011;129(3):153-164. doi:10.1590/S1516-31802011000300006

4. Langerová P, Vrtal J, Urbánek K. Adverse Drug Reactions Causing Hospital Admissions in Childhood: A Prospective, Observational, Single-Centre Study. *Basic Clin Pharmacol Toxicol*. 2014;115(6):560-564. doi:10.1111/bcpt.12264

5. Mouton JP, Fortuin-de Smidt MC, Jobanputra N, et al. Serious adverse drug reactions at two children's hospitals in South Africa. *BMC Pediatr*. 2020;20(1):3. doi:10.1186/s12887-019-1892-x

6. Duczmal E, Bręborowicz A. Adverse drug reactions as a cause of hospital admission. *Przeglad Pediatryczny*. 2006;36(1):14-18.

7. Bellis JR, Kirkham JJ, Nunn AJ, Pirmohamed M. Adverse drug reactions and off-label and unlicensed medicines in children: a prospective cohort study of unplanned admissions to a paediatric hospital: Adverse drug reactions and off-label and unlicensed medicines in children. *Br J Clin Pharmacol*. 2014;77(3):545-553. doi:10.1111/bcp.12222

8. Posthumus AAG, Alingh CCW, Zwaan CCM, et al. Adverse drug reaction-related admissions in paediatrics, a prospective single-centre study. *BMJ Open*. 2012;2(4):e000934. doi:10.1136/bmjopen-2012-000934

9. Impicciatore P, Mohn A, Chiarelli F, Pandolfini C, Bonati M. Adverse drug reactions to off-label drugs on a paediatric ward: An Italian prospective pilot study. *Paediatr Perinat Drug Ther*. 2002;5(1):19-24. doi:10.1185/146300902322125118
